# Distinct cell states define larval and adult body plans in a hemichordate

**DOI:** 10.1038/s41467-026-77191-y

**Published:** 2026-09-08

**Authors:** Paul Bump, Carolyn Brewster, Laurent Formery, Lauren Lubeck, Catherine Campbell, Maurizio Morri, Rene Sit, Daniel S. Rokhsar, Blair Benham-Pyle, Alejandro Sánchez Alvarado, Christopher J. Lowe

**Affiliations:** 1 Hopkins Marine Station, Department of Biology, Stanford University, Pacific Grove, CA USA; 2 Department of Biology, Pomona College, Claremont, CA USA; 3 Stowers Institute for Medical Research, Kansas City, MO USA; 4 Molecular and Cell Biology Department at the University of California, Berkeley, CA USA; 5 Unit on Developmental Signaling, Division of Developmental Biology, Eunice Kennedy Shriver National Institute of Child Health and Human Development, NIH, Bethesda, MD USA; 6 Department of Biology, Johns Hopkins University, Baltimore, MD USA; 7 CZ Biohub, San Francisco, CA USA; 8 Okinawa Institute of Science and Technology, Onna, Okinawa, Japan; 9 Stem Cell and Regenerative Medicine Center, Department of Molecular and Cellular Biology, Baylor College of Medicine, Houston, TX USA

**Keywords:** Evolutionary developmental biology, Gastrulation, Biological metamorphosis

## Abstract

A major gap in our understanding of animal development is how adult body plans arise in animals with indirect development, where adults emerge from the transformation of a distinct larval form during metamorphosis. We address this question by examining cellular changes in the enteropneust hemichordate *Schizocardium californicum*, a species with a complex lifecycle and dramatic metamorphosis. Employing whole-body single-cell RNA sequencing, we chart the cellular composition and transcriptional dynamics of larval, metamorphic, and adult stages. Our tissue-level atlas reveals that ectodermal and endodermal cell types in larvae and adults occupy distinct transcriptional spaces, showing greater similarity to other cell types within the same life stage than to their counterparts in the opposite stage. In contrast, mesodermal cell types from both larvae and adults cluster closely together, indicating conserved transcriptional profiles. These findings demonstrate that the extensive morphological reorganization during metamorphosis is accompanied by broad shifts in transcriptional identity and reveal life-history stage as a major organizing axis of cellular state during the larva-to-adult transition.

## Abstract (web summary)

Here they use scRNA-seq to show that, in the hemichordate Schizocardium californicum, ectoderm and endoderm shift transcriptional identity from larva to adult while mesoderm is transcriptionally conserved, making life stage a major axis of cellular state.

## Introduction

**Fig. 1:**
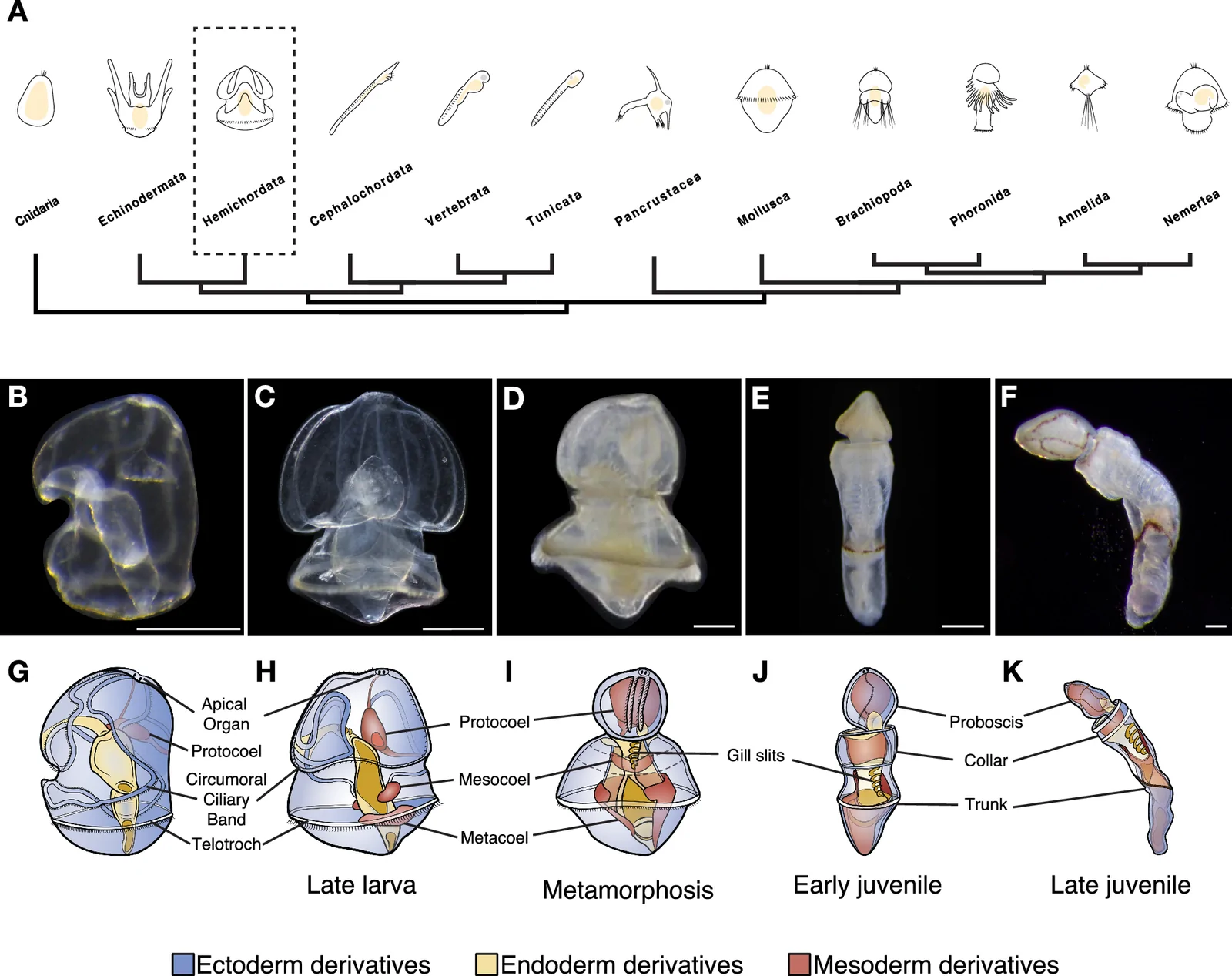
Indirect developing hemichordates as a model to study the transition between larval and adult body plans. **A** Phylogenetic distribution of bilaterians highlighting the prevalence of larval types. Light microscopy of the major life history stages of *S. californicum*: **B** early larva, **C** late larva, **D** during metamorphosis, **E** early juvenile, **F** late juvenile. Representative images from where a minimum of three images were collected from 3 separate spawns. Scale bars are 500 µm. Schematic diagrams of the major life history stages of *S. californicum*: **G** early larva, **H** late larva, **I** during metamorphosis, **J** early juvenile, **K** late juvenile. In the schematics, ectoderm derivatives are shown in blue, endoderm derivatives in yellow, and mesoderm derivatives in red.

Our understanding of adult development is largely driven by the detailed study of a few species, mostly direct developers, defined by the formation of the adult body plan during embryogenesis^1–3^. However, this type of developmental strategy does not represent the full diversity of animal life histories, and in many species the adult is formed through metamorphosis – the transformation of a larval body plan into an adult – often weeks to months after embryogenesis (Fig. 1A)^4,5^. Although species are largely categorized as either direct or indirect developers, developmental strategy is far less categorical and instead developmental mode largely operates along a continuum^6,7^. At one extreme are direct developers where adults form straight from the embryo and there are no intervening larval structures^8–10^. At the other end of the spectrum are species where the larval and adult body plans are developmentally and morphologically uncoupled with the adult formed by transformation of larval tissue during metamorphosis^2^. Most developmental model species lie closer to the direct end of the spectrum; even species with an ecologically significant larvae, like the frog *Xenopus laevis*, form the main elements of the chordate adult body plan during embryogenesis. On the other end of the continuum, sea urchins represent the extreme limit of indirect development: their embryos give rise to a bilaterally symmetric, planktonic larva that is both morphologically and axially very distinct from the radial adult body plan that forms by a drastic body reorganization during metamorphosis^11,12^.

Indirect development has a broad phylogenetic distribution across bilaterians and has even been proposed to represent the ancestral bilaterian developmental strategy^9,13^. However, our understanding of developmental mechanisms in animals defined by indirect development is largely limited to embryogenesis and early larval patterning. How the adult body plan forms by the transformation of the larval body plan rather than from embryonic development is poorly characterized^6,7^.

Recently, single-cell RNA sequencing approaches have expanded our understanding of developmental trajectories and cell type diversification. However, the fate of larval cells and the dynamics of cellular changes during metamorphosis remain poorly understood. Comparative work in single-cell genomics has either focused on direct developers or on embryonic and early larval stages, excluding the later adult life history stages in animals with complex life cycles^14–18^. For a comprehensive understanding of developmental mechanisms through metamorphosis, it is essential to have insights into the cellular composition of larvae and adults and how these may change through metamorphosis. Recent studies are beginning to reveal that this perspective is essential to fully evaluate the cellular diversity represented by particular lineages, to test hypotheses of cell type evolution, and to understand the evolution of animal body plans^19–23^.

Hemichordates are a deuterostome phylum with a relatively simple adult body plan. As the sister group to echinoderms, they occupy a key phylogenetic position for investigating deuterostome and chordate evolution. Their combination of conserved axial patterning programs and comparatively simple morphology has also made them an important system for testing hypotheses of bilaterian body plan evolution^24^. The enteropneust hemichordate *Schizocardium californicum* is an excellent model for the study of indirect development^25^ (Fig. 1B–F). Like many enteropneusts, *S. californicum* develops via a tornaria larva that is organizationally similar to echinoderm larvae, with two primary ciliary bands used for swimming and feeding, and limited musculature^26–28^. This type of larva has been termed “head larvae” as their body plan is largely defined by regulatory networks that define an anterior “head” territory in bilaterians and are missing a Hox-patterned trunk domain^29–31^. The formation of the adult through a radical metamorphosis transforms the planktonic larva into a burrowing benthic adult and involves extensive remodeling of the ectoderm, rapid expansion of the mesoderm, reorganization of the endoderm, and the formation and extension of the trunk territory. Adults are adapted for a burrowing lifestyle using a muscular proboscis, and feed using both mucociliary particle capture and filter feeding through their pharyngeal gill slits^32,33^. The tripartite gut is structurally similar between larva and adult, but the gill slits begin to develop in the late larva in the anterior gut and perforate the anterior trunk of the adult after a posterior migration during metamorphosis^34^. The larval nervous system is defined by an apical organ and neurons associated with the feeding ciliary band, whereas the adult has an extensive basiepidermal plexus and both a dorsal and ventral nerve cord^35–38^.

While morphological changes during enteropneust metamorphosis are extensive, the axial register between life histories is maintained along with the original mouth and anus. Previous work suggests that metamorphosis does not involve extensive larval cell death and larval cells may be carried over into the adult^39^, raising the question of the fate of larval cells in the adult body plan.

Here we identify similarities and differences in cellular composition of the larval and adult body plan of *S. californicum* using single-cell transcriptomics at time points representing larval, metamorphic, and juvenile stages. We assess the degree to which cell types overlap between life histories and whether metamorphosis represents not only a major morphological change in body plan organization, but also a change in cellular composition. We find that larval and juvenile cells largely occupy distinct transcriptional states and show greater similarity to other tissues within the same life-history stage than to their putative counterparts across stages. Additionally, pulse-chase EdU experiments through metamorphosis demonstrate the persistence of labeled larval cells into juveniles, arguing against wholesale larval cell replacement and suggesting extensive remodeling of cellular transcriptional states during metamorphosis.

## Results

### Sampling distinct life history stages in the same organism

To understand the cell types that make up distinct larval and juvenile animals, we sampled five distinct life history stages in *S. californicum*: early larva, late larva, metamorphosis, early juvenile, and late juvenile based on the previous description of development^40^ (Fig. 1B–F) and which are generally representative of other, more distantly related indirect-developing enteropneust species such as *Ptychodera flava* and *Balanoglossus simodensis*.

#### Early larva

The first larval stage (22 days post-fertilization; Fig. 1B,G) exhibits a fully established larval body plan without adult structures. The tornaria is a small, planktonic feeder propelled by two ciliary bands: an anterior circumoral loop for feeding and a posterior telotroch for locomotion^27,41,42^. The remaining ectoderm is largely squamous, with a ventral neurotroch and an anterior apical organ bearing a ciliary tuft, neurons, and paired eyespots^40^. Mesoderm is minimal, consisting of a single anterior protocoel and limited musculature associated with the pharynx and apical strand^42,43^. The endoderm forms a tripartite gut (pharynx, stomach, intestine) terminating in an anus.

#### Late larva

By two months (Fig. 1C,H), larvae are larger, feeding more extensively, and competent for metamorphosis^44^. While retaining overall morphology, this stage shows early adult patterning, including formation of mesocoels and metacoels and the appearance of nascent gill slits. The posterior expands into an anal field, and the nervous system becomes more elaborate, with widespread serotonergic neurons and initial development of the dorsal cord^40^.

#### Metamorphosis

At mid-metamorphosis (Fig. 1D,I), larval and adult features coexist. Ectodermal thickening and coelomic expansion accompany trunk initiation as the blastocoel is reduced^34,45^. The degenerating circumoral band contributes to the proboscis ectoderm, while the anterior coelom expands to form proboscis musculature. The stomochord forms at the proboscis base and supports the heart/kidney complex^46^. Nascent gill slits shift posteriorly as trunk elongation begins, and collagenous skeletal elements initiate. Animals remain planktonic and swim using the telotroch.

#### Juveniles

Early (Fig. 1E,J) and late (Fig. 1F,K) juveniles represent the definitive adult body plan. These benthic worms exhibit the tripartite organization of proboscis, collar, and trunk, with a ventral mouth, anterior trunk gill pores, and both dorsal and ventral nerve cords. Early juveniles have a developed proboscis and collar but limited trunk elongation, whereas late juveniles show substantial trunk extension, capturing the full cellular complement of the adult form.

### Single-cell sequencing reveals the diversity of hemichordate cell types

To understand the cellular composition of each of these distinct life history stages, whole organism cell suspensions were prepared for single-cell RNA sequencing (scRNA-seq). A total of 87,021 single-cell profiles were sequenced: 15,403 cells for early larva, 22,027 for late larva, 13,771 during metamorphosis, 20,061 for early juvenile, and 15,759 for late juvenile.

**Fig. 2:**
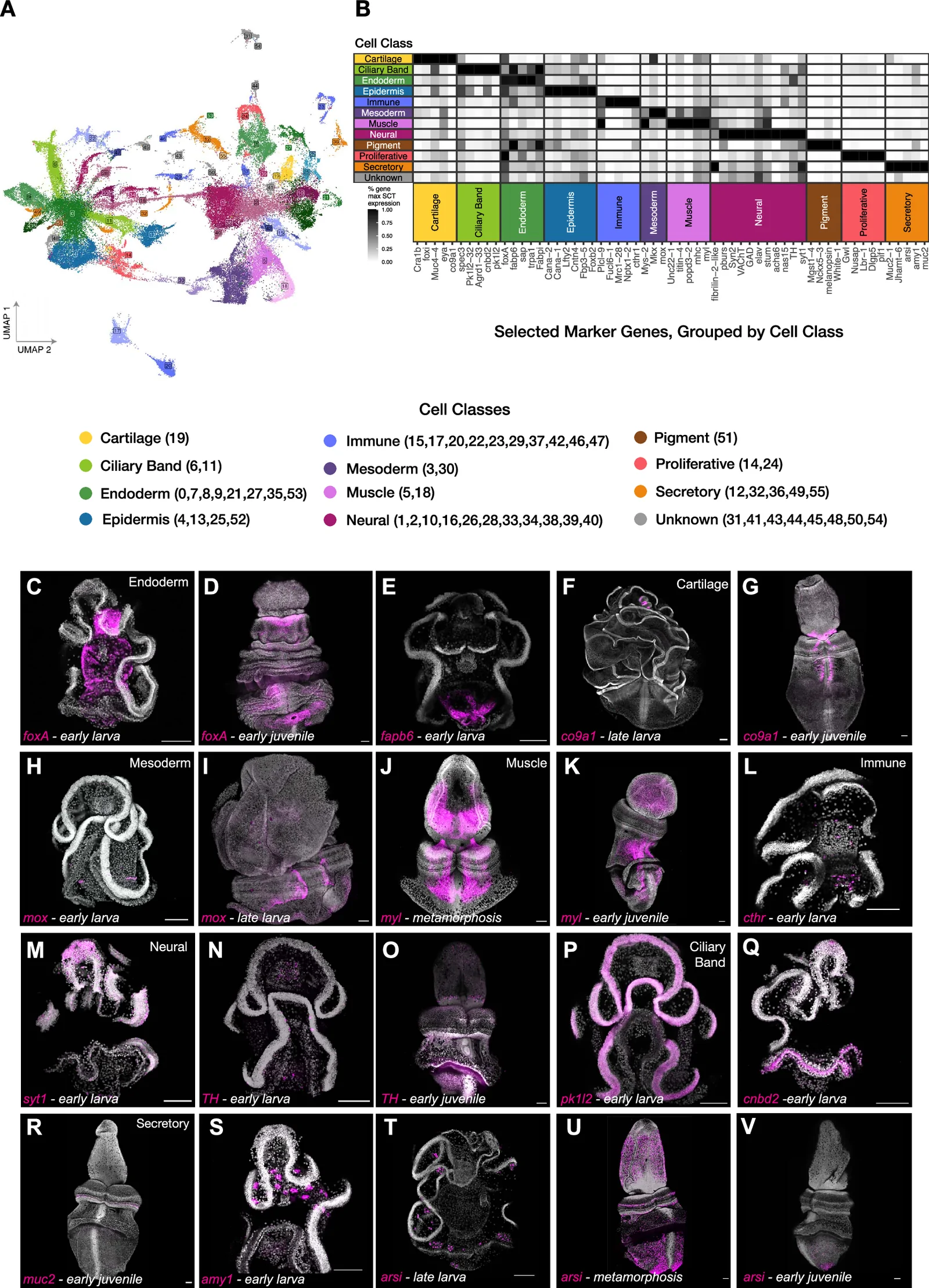
Single-cell sequencing reveals the broad diversity of hemichordate cell types. **A** UMAP embedding of the cell classes of *S. californicum;* 55 clusters were identified and 12 cell classes were described. **B** Selected markers of cell classes of *S. californicum*, percentage of gene maximum was calculated by taking the mean normalized expression of the gene of interest and dividing by the maximum for any cell class. **C**–**T** HCR signal in magenta, nuclei are in gray, scale bars are 100 µm. Each HCR experiment was imaged in triplicate. **C** *foxA* HCR expression in early larva. **D** *foxA* HCR expression in early juvenile. **E** *fabp6* HCR expression in early larva. **F** *co9a1* HCR expression in late larva. **G** *co9a1* HCR expression in early juvenile. **H** *mox* HCR expression in early larva. **I** *mox* HCR expression in late larvae. **J** *myl* HCR expression during metamorphosis. **I** *myl* HCR expression in early juvenile. **L** *cthr* expression in early larva. **M** *syt1* HCR expression in early larva. **N** *TH* HCR expression in early larva. **O** *TH* HCR expression in early juvenile. **P** *pk1l2* HCR expression in early larva. **Q** *cnbd2* HCR expression in early larva. **R** *muc2* HCR expression in early juvenile. **S** *amy1* HCR expression in early larvae. **T** *arsi* HCR expression in late larvae. **U** *arsi* HCR expression during metamorphosis. **V** *arsi* HCR expression in early juvenile.

We generated an atlas of *S. californicum* across life history stages by clustering cellular profiles, identifying 56 clusters containing between 68 and 8392 cells (average 1554 cells/cluster) (Fig. 2A, Supplementary Fig. 1A, Supplementary Data 1). Using the top cluster-specific genes, known hemichordate markers, and functional annotation, we organized these cell type clusters into 12 cell classes (cartilage, ciliary band, endoderm, epidermis, immune cells, mesoderm, muscle, neural, pigment, proliferative, secretory, and unknown) (selected markers in Fig. 2B, full markers in Supplementary Data 2 and Supplementary Figs. 2−8). Cell clusters are defined by markers obtained by analysis of differential expression (log fold change and *p*-value) and specificity (Supplementary Data 3).

We validated our cell type clusters using known marker genes from other species, newly discovered markers from our cluster annotations and known tissue markers in hemichordates, by plotting the relative expression of selected markers in each cell class (Fig. 2B) and then performing HCR (hybridization chain reaction) fluorescent in situ hybridization (Fig. 2C–V, Supplementary Data 4).

#### Endoderm derivatives

*Endoderm clusters: 0, 7, 8, 9, 21, 27, 35, 53* (Fig. 2C–E, Supplementary Fig. 2A–D). Differentially expressed genes for endoderm include fatty acid binding proteins and *foxA*, a known endoderm marker in hemichordates^47,48^. *FoxA* is found in all eight of our endoderm clusters, with limited expression outside of this group. It is expressed throughout the tripartite gut of *S. californicum* larvae in the mouth, stomach, and intestine (Fig. 2C and 2D), consistent with prior observations in *S. kowalevskii*^47^. As in *S. kowalevskii, S. californicum* juveniles *foxA* is expressed in the anterior collar groove, but with a broader expression domain in the ectoderm (Fig. 2D). We also identified additional markers from our single-cell analysis that enabled finer-scale characterization of endoderm. For example, the gut marker *fabp6*, which encodes a fatty acid binding protein family member responsible for lipid balance and retinol- and retinoic acid-binding across animals^49^, is highly expressed in the larval intestine (Fig. 2E) while *trpa1* is expressed in the larval stomach (Supplementary Fig. 2C).

*Cartilage cluster: 19* (Fig. 2F–G, Supplementary Fig. 2E–H). A single cluster was enriched for several cartilage markers, including *collagen alpha-1(IX) chain-like* (*co9a1*). Since cartilage in enteropneust hemichordates is secreted by the endodermal pharyngeal epithelium^50^ and the gill slits specifically are made up of fibrillar collagen^51^, we expected *co9a1* expression would be localized to the gill slits, a characteristic deuterostome feature. We found that *co9a1* is expressed in a small pocket of cells in the late larva in the developing gill slits (Fig. 2F), through metamorphosis (Supplementary Fig. 2G), and in juveniles in the gill bars and the wishbone-shaped proboscis skeleton (Fig. 2G). This cluster also expresses *foxI*, a marker implicated in gill pouch endoderm and in the posterior gut (Supplementary Fig. 2F)^47^.

#### Mesoderm derivatives

*Mesoderm clusters: 3 and 30* (Fig. 2H–I, Supplemental Fig. 3A–B). Differentially expressed genes in the mesoderm clusters included *mesenchyme homeobox* (*mox*), a gene expressed in the paired posterior coelomic cavities in *S. kowalevskii*^52^. Using HCR, we detected *mox*-positive cells in *S. californicum* during initiation of the posterior coelom formation prior to any clear morphological outpocketing of mesoderm (Fig. 2H), as well as in clearly defined celoms of the late larva (Fig. 2I).

**Fig. 3:**
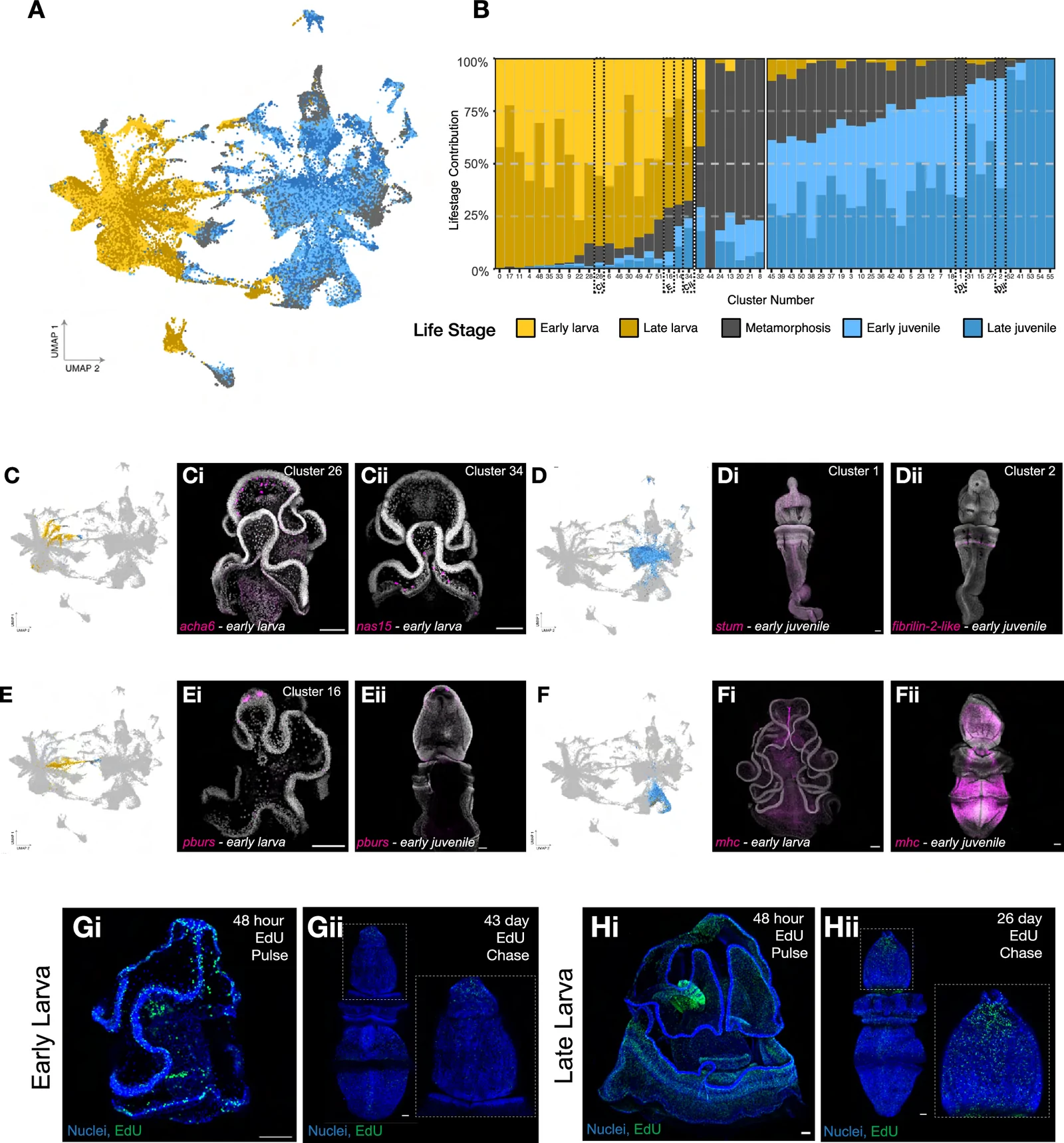
Divergence of transcriptional states is consistent with morphological changes between life history stages. **A** UMAP embedding of *S. californicum* single-cell RNA-seq data with cells colored by life stage. Yellow: early larva, dark gold: late larva, gray: metamorphosis, light blue: early juvenile, dark blue: late juvenile. **B** Bar chart of life stage composition of clusters. **C**–**F** HCR signal in magenta, nuclei are in gray, scale bars are 100 µm. Each HCR experiment was imaged in triplicate. **C** UMAP embedding with larval neural clusters (26, 28, 33, 34) colored by life stage. **Ci** *acha6* HCR expression in early larvae. Cii) *nas15* HCR expression in early larvae. **D** UMAP embedding with juvenile neural clusters (1, 2) colored by life stage. **Di** *stum* HCR expression in early juvenile. Dii) *fibrilin-2-like* HCR expression in early juvenile. **E** UMAP embedding with neural cluster 16 colored by life stage. **Ei** *pburs* HCR expression in early larvae. Eii) *pburs* HCR expression in early juvenile. **F** UMAP embedding with muscle clusters (5, 18) colored by life stage. **Fi** *mhc* HCR expression in early larvae. **Fii** *mhc* HCR expression in early juvenile. **Gi**, **Gii**, **Hi**, **Hii** EdU signal in green, nuclei are in blue, scale bars are 100 µm. Each EdU experiment was imaged in triplicate. **Gi** EdU pulse of early larva for 48 hours. **Gii** EdU chase after 43 days once larva have completed metamorphosis, inset is of proboscis region of the juvenile worm, boxed. **Hi** EdU pulse of late larva for 48 hours. **Hii** EdU chase after 26 days once larvae have completed metamorphosis, inset is of proboscis region of the juvenile worm, boxed.

*Muscle clusters: 5 and 18* (Fig. 2J–K, Supplementary Fig. 3D–H). Enteropneust adults are highly muscular, and muscle clusters were identified in our data set by the expression of muscle components such as *titin, popeye domain-containing protein 3*, and *myosin light chain (myl*) (Supplementary Fig. 3E, F). Expression of *myl* was detected in all life stages and was particularly prevalent near the end of metamorphosis where longitudinal fibers are forming to give rise to the musculature of the juvenile worm (Fig. 2J, K).

*Immune clusters: 15, 17, 20, 22, 23, 29, 37, 42, 46, 47* (Fig. 2L, Supplementary Fig. 4A–C). scRNA-seq allowed us to characterize many previously undescribed cell types including putative immune cells. We found a number of immune populations that expressed macrophage mannose receptors including C-type lectins expressed by most tissue macrophages and dendritic cells^53,54^, as well as a specific population of cells (cluster 17) marked by the expression of *collagen triple helix repeat-containing protein* (*cthr*) (Fig. 2L), a secreted glycoprotein implicated in tissue repair processes^55^.

#### Ectodermal derivatives

*Neural clusters: 1, 2, 10, 16, 26, 28, 33, 34, 38, 39, 40* (Fig. 2M–O, Supplementary Fig. 5). We identified neural cell clusters by the expression of *synaptotagmin* (*syt1*), which is involved in synapse docking^56^ (Fig. 2M) as well as *elav*, a neuron-specific RNA binding protein expressed in differentiated bilaterian neurons^57–59^, but as noted in other species, it does not mark all neurons^60,61^. Neural subtypes were identified by the expression of enzymes involved in the synthesis of neurotransmitters or transporters: *tyrosine hydroxylase* (*TH*), which marks dopaminergic neurons (Fig. 2N, O), the GABAergic marker *GAD* (Supplementary Fig. 5C), cholinergic marker *VAChT* (Supplementary Fig. 5D), and serotonergic marker *Tph* (Supplementary Fig. 5F, G). We observed these neural cell types in both larvae and juveniles.

*Ciliary band clusters: 6 and 11* (Fig. 2P-Q, Supplementary Fig. 4D-G). The tornaria larva is characterized by two distinctive ciliary bands, the circumoral ciliary band and the telotroch (Fig. 1B, C, G, H). Clusters 6 and 11 expressed known ciliary markers. Cluster 6 showed enrichment of *polycystic kidney disease protein 1-like 2* (*pk1l2*), which has been associated with the primary apical cilium of renal epithelia^62–64^. When we examined the expression of *pk1l2* via HCR imaging, we found that it labeled the circumoral ciliary band but not the telotroch (Fig. 2P). Cluster 11 was marked by *cyclic nucleotide-binding domain containing protein* (*cnbd2*), which may be involved in ciliary beating^65,66^. HCR imaging showed that *cnbd2* had a distinct expression pattern in the telotroch (Fig. 2Q).

*Secretory clusters: 12, 32, 36, 49, 55* (Fig. 2R-V, Supplementary Fig. 6A-B). We found several clusters in both larvae and juveniles that were enriched for mucins and glycoproteins and organized these clusters into a functional secretory cell class. We propose that cluster 12 is a secretory cell population based on the high expression of *mucin2 (muc2)* (Supplementary Fig. 6B). Mucins have been implicated as important for muco-ciliary feeding in hemichordates^67^. HCR imaging showed *muc2* expressed in a thin circumferential band of cells in the collar of the juvenile (Fig. 2R), similar to the expression of mucins in *S. kowalevskii*^68^. In hemichordates, the epidermis is highly secretory, producing large amounts of mucus. Even for secretory clusters with a smaller number of cells (374 for cluster 49), we were able to recover distinct patterns of cell type-specific gene expression. Cluster 49 is characterized by expression of *alpha-amylase* (*amy1*). Alpha-amylases hydrolyze 1,4-alpha-glucoside bonds in oligosaccharides and polysaccharides in the salivary gland of humans^69^. HCR imaging showed *amy1* expression in a distinctive pattern of cells in the blastocoel surrounding the larval stomach (Fig. 2S, Supplementary Fig. 6C), so we propose that this cell population may be involved in secretory function. Finally, we found a cluster of secretory cells with a striking shift in abundance and distribution over developmental time. The markers of a putative secretory cluster 32 included *arylsulfatase* (*arsi*), a protease that is upregulated in the *Xenopus laevis* tadpole tail as an integral part of the resorption process during metamorphosis^70^. In the late larva, these *arsi*+ cells are found in clusters throughout the ectoderm (Fig. 2T), become broadly distributed during metamorphosis (Fig. 2U), and finally are predominantly restricted to the most posterior of the early juvenile worm (Fig. 2V). Future work will be necessary to investigate the function of this intriguing cell cluster during metamorphosis.

### Divergence of transcriptional states between life histories

Given that we had sampled the single-cell composition of five life history stages with distinct morphologies and ecological demands, we wanted to understand how cellular composition relates to life history. We considered three possible scenarios: First, that larval cell types represent a subset of adult cell types; second, that the cells of larval and adult cells share transcriptional identity, while each stage also possesses unique cell types; and third, that larval and adult body plans are composed of distinct cell types with limited overlap in transcriptional identity.

To test these possibilities, we plotted life history on our scRNA-seq clusters (Fig. 3A). We observed a striking pattern when comparing the distribution of cells across the five different life history stages: larval and juvenile stages largely clustered in distinct regions of transcriptional space (Fig. 3A). To confirm that this observation was not an artifact resulting from our chosen methods for integrating cells from different stages into a single transcriptional space (See Material and Methods, Supplementary Fig. 1), we performed “Harmony“^71^ and “Portal”^72^ integrations, which still resulted in spatially separated clustering of larval and juvenile life stages (Supplementary Fig. 1B and 1C).

To examine changes in cell type compositions between life stages, we looked at the proportion of cells in each cluster from every timepoint (Fig. 3B). Broadly, these life stage contributions matched our expectations of the morphology of the organism: some tissues are composed almost entirely of juvenile cells, like cartilage (cluster 19), while others are predominantly larval cells, such as the ciliary band (clusters 11 and 6). Consistent with the stage-related decrease in the number of cells in ciliary band clusters, we showed that the expression of markers of cluster 6 such as *pk1l2* decrease over time and are restricted to the circumoral ciliary band and future oral groove of the proboscis during metamorphosis (Supplementary Fig. 4F). This aligns with known morphology and with our previous study that found a higher level of cell death in the circumoral ciliary band^39^.

Based on our annotation, neural cells represent one of the most broadly distributed cell classes across both larval and juvenile life-history stages. The neural cell class encompasses clusters 1, 2, 10, 16, 26, 28, 33, 34, 38, 39, and 40, which are widely dispersed across the global UMAP embedding (Supplementary Fig. 5A). Despite this dispersion, examination of cluster-specific markers (Supplementary Data 3) revealed shared neural-associated molecular signatures that allowed these clusters to be grouped together into a broader neural cell class (Fig. 2B; Supplementary Fig. 5B).

Cells from the neural class recovered substantial organization by life-history stage, indicating that broadly conserved neural identity is accompanied by stage-specific transcriptional states (Supplementary Fig. 5H). Within this broader neural class, individual clusters also exhibit distinct molecular profiles. For example, cluster 16 is enriched for genes previously associated with hemichordate neural patterning such as *t-brain*, whereas cluster 1 is enriched for neural genes in other phyla that have not been previously characterized in hemichordate neurobiology, such as *ox2r* (orexin receptor type 2) and *syt11* (synaptotagmin-11). We also identified genes broadly shared across the neural class, including the known neural marker *rhg39* (Rho GTPase-activating protein 39).

To examine these life-history-related neural differences in greater detail, we next focused on neural clusters 26, 16, 34, 1, and 2 (dashed boxes in Fig. 3B, corresponding to panels in Fig. 3C–E).

There were neural clusters composed of predominately larval cells (Fig. 3C), such as cluster 26 (Fig. 3Ci) which was marked by expression of *nas15*, a zinc metalloproteinase, which is a part of a large family of astacins, some of which are expressed in the nervous system (Park et al., 2010). This cluster also expressed *lysine-specific demethylase 8* (*kdm8-7*), an important regulator of neurite morphogenesis^73^. We found *nas15* expressed throughout the posterior of the larva in cells of the developing telotroch (Fig. 3Ci). This neural cell type is absent in juvenile life stages, likely due to the loss of ciliary band at metamorphosis. Neural cluster 34 expressed *dopamine beta-hydroxylase* (*dbh*), which converts dopamine to noradrenaline^74^ and *neuronal acetylcholine receptor subunit alpha 6* (*acha6*), which belongs to the ligand-gated ion channel superfamily, including GABA, glycine, and 5-HT3 receptors^75^. We found *acha6+* cells distributed around the ventral anterior of the larva (Fig. 3Ci). Other neural cell clusters were composed of cells that were predominantly from the juvenile stage, such as clusters 1 and 2 (Fig. 3D). Cluster 1 was marked by the expression of *stum*, which has been implicated in mediating mechanical sensing in receptor neurons and is a transducer for mechanical stimuli^76^. HCR showed *stum* expression along the dorsal and ventral nerve cords of the juvenile and projecting into the proboscis on the dorsal surface (Fig. 3Di). Neural cluster 2 is marked by *fibrilin-2-like*, a large modular structural glycoprotein component of the extracellular matrix of peripheral nerves^77^. It is expressed in the collar of juveniles, with an enrichment of *fibrilin-2-like*+ cells with neural morphology at the base of the collar (Fig. 3Dii). Neural cluster 16 is present in both life history stages and is marked by *partner of bursicon* (*pburs*), which together with bursicon forms a neuropetide heterodimer broadly conserved in bilaterians^78^ with a well characterized role in arthropod eclosure following metamorphosis^79^ (Fig. 3E). We detected expression of *pburs* in the eyespots as part of the larval apical organ (Fig. 3Ei)^80,81^. At later larval stages, expression continued in the eyespots (Supplementary Fig. 5E) and was persistent into the juvenile in a similar expression territory, in two patches at the tip of the proboscis (Fig. 3Eii). While the tuft of cilia associated with the apical organ degenerates at metamorphosis, some of the neurons and the eyespots appear to persist through metamorphosis, at least in juvenile stages^40^.

Finally, as described above, while many cell types had larval- or juvenile-specific transcriptional signatures, muscle (clusters 5 and 18) was an interesting exception (Fig. 3F). A highly differentially expressed gene in these muscle clusters is *myosin heavy chain* (*mhc*). One of the few muscle types that is present in the larva is the apical strand muscle, which is marked by the expression of *mhc* (Fig. 3Fi). During metamorphosis and into the early juvenile stage, *mhc* expression expands into the distinctive musculature of the juvenile worm (Fig. 3Fii, Supplementary Fig. 3G). While the prevalence and distribution of muscle cells change over developmental time in the animal, our single-cell analysis suggests that the transcriptional signature of these clusters remains essentially unchanged as they do not show spatially separated clustering of larval and juvenile cells (Fig. 3F).

Given the changes that we observed in the transcriptional profiles, we considered two principle possibilities: (1) extensive larval cell death during metamorphosis followed by proliferation of adult stem cell populations, or (2) larval cells are carried over into the juvenile animal with modified patterns of gene expression. Extensive larval cell death at metamorphosis is not supported by our previous observations^39^, so to further explore the possibility of cells being carried over from larval to juvenile, we performed a series of EdU (5-ethynyl-2’-deoxyuridine) pulse-chase experiments through metamorphosis. We incubated early and late larvae with EdU for 48 hours to label any cell in S-phase (Fig. 3Gi, 3Hi), then washed out the EdU label and let the animals continue to grow and develop until they completed metamorphosis. In an early larva, a 48 hour pulse indiscriminately labels a number of different cellular populations including those in the ectoderm (ciliary band), mesoderm (wrapped around the gut), and endoderm (in the mouth and gut) similar to what we had observed with a short EdU pulse previously (Fig. 3Gi)^39^. Forty-three days later, we found that some EdU+ cells were still present, particularly in the anterior proboscis and along the dorsal midline (Fig. 3Gii), indicating that at least some cells from the larval body plan persist through to the juvenile stage. In late larvae, closer to metamorphosis, 48 hour pulses label many cell populations within all three germ layers (Fig. 3Hi). These cell populations include the ciliary bands, the epithelium of the presumptive collar and proboscis ectoderm, the mesocoel and metacoel, and a large enrichment in the gill bars, consistent with previous reports of shorter EdU pulse-chase experiments at this particular stage^39^. In this case, EdU+ cells persisted in the juvenile, with a strong enrichment in proboscis, collar, and dorsal cord (Fig. 3Hii). Additionally, after a pulse-chase experiment, we performed HCR for *fibrilin-2-like*, a marker that was enriched in a juvenile neural cluster and found that there are some EdU+ cells from the larva which express a juvenile neural marker (Supplementary Fig. 5I, J). From these data, we conclude that larval cells may be carried into the juvenile animal with possible changes in transcriptional profile.

### Differences between cells in larval and adult body plans

**Fig. 4:**
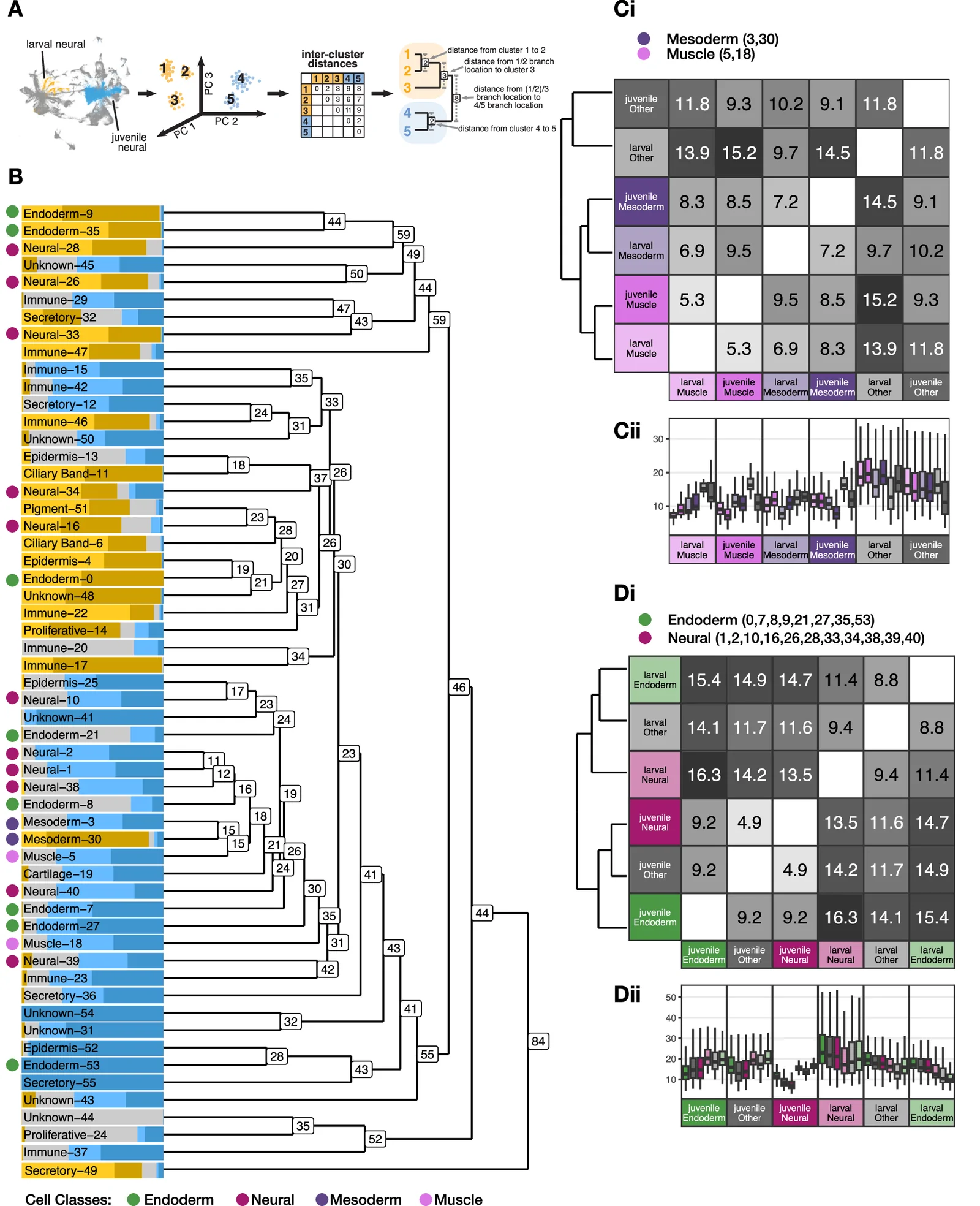
Mesodermal tissues share transcriptional similarity while ectodermal and endoderm tissues reflect life stage divergence. **A** Schematic diagram of approach used to accurately quantify similarity or dissimilarity of clusters in 55-dimensional PC space. **B** Dendrogram shows a hierarchical clustering of scRNA-seq clusters using the centroid of each cluster in 55-dimensional PC space, with bars colored by cluster life stage composition per Fig. 3B. Numbers at nodes indicate the distance between the centroids of the two branches merging at that node, as a percent of the largest inter-cluster distance. Colored dots highlight tissues that are further analyzed in Figs. **Ci**, **Cii**, **Di**, **Dii**. **Ci**, **Cii** Comparison of larval mesoderm and larval muscle versus juvenile mesoderm and juvenile muscle, with “Other” denoting all other cells from that life stage. **Ci** Heatmap indicates inter-centroid distances as a percent of the largest inter-cluster distance, a larger value means centroids are farther apart while a smaller value means centroids are closer. **Cii** Box plots show the distribution from the cells of a group (panel) to the centroids of each other group (bars). **Di**, **Dii** Comparison of larval neurons and larval endoderm versus juvenile neurons and juvenile endoderm, with “Other” denoting all other cells from that life stage. **Di** Heatmap indicates inter-centroid distances as a percent of the largest inter-cluster distance **Dii** Box plots show the distribution from the cells of a group (panel) to the centroids of each other group (bars). In box plots, the lower and upper hinges correspond to the first and third quartiles. The upper whisker extends from the hinge to the largest value no further than 1.5 * IQR from the hinge (where IQR is the inter-quartile range, or distance between the first and third quartiles). The lower whisker extends from the hinge to the smallest value at most 1.5 * IQR from the lower hinge. Horizontal bars indicate medians.

We further quantified and interrogated the overall transcriptional relationships between cell types and life stages using our scRNA-seq dataset. While two- or three-dimensional embeddings like UMAP plots (Fig. 2A, Fig. 3A) are convenient for visual analysis, low-dimensional representations unavoidably distort complex relationships among large numbers of clusters^82^. To more accurately quantify similarity (closeness) or dissimilarity (remoteness) of clusters in 55-dimensional principal component (PC) space, we calculated the centroid for the PC embeddings of the cells in each cluster and then calculated pairwise distances between cluster centroids (Fig. 4A). These distances were used to form a hierarchical clustering, and we calculated the PC-space distance at each branch point (Fig. 4B). Additionally, we implemented a neighbor-joining approach similar to Musser et al., 2021 which recovered relationships broadly consistent with our centroid-based analyses, supporting the robustness of the observed cell-type associations across life-history stages (Supplementary Fig. 10). In our centroid-based analysis, we found that cluster 3 and 30 cluster together, which together defined the broad mesodermal cell type, even though cluster 3 was primarily composed of juvenile cells and cluster 30 was primarily composed of larval cells (Fig. 4B). Larval gut clusters 9 and 35 are closer to larval neural clusters 28 and 26 than to other adult clusters from the juvenile. This is surprising as these cell types arise from separate germ layers, the endoderm and ectoderm. Adult neural clusters 38, 1, 2, and 40 are transcriptionally more similar to adult gut clusters 21, 8, and 27 than they are to larval neural clusters (Fig. 4B). These findings supported our predictions based on the observations of the UMAP that some cell types are more similar between life stages than they are between cell types.

We next quantified whether transcriptional shifts were larger between cell types in the same life stage or between life stages of the same cell type. To do this, we grouped the cells first by tissues of interest, then split those groups by life stage (all cells outside the tissue of interest are grouped into “Other”). As before, we found the centroids of these groups in transcriptional space and calculated pairwise distances between them, then used these to generate a hierarchical clustering (Fig. 4Ci, 4Di). To help evaluate how representative each centroid was of the position of its cells in transcriptional space, we also calculated the distance from individual cells in each group to each group centroid (Fig. 4Cii, 4Dii).

Interestingly, we found that larval mesodermally-derived cells, either mesoderm or muscle, are more transcriptionally similar to juvenile mesodermal cells than they are to other larval cell types (Fig. 4Ci-4Cii). This is counter to what we observed for many of the endodermally- and ectodermally-derived cell types. For example, larval neurons and larval gut cells are generally more transcriptionally similar than either is to adult neurons or adult gut cells, respectively (Fig. 4Di-Dii). This was suggested by the space they occupy in low-dimensional embedding on the UMAP but bolstered by higher-dimensional analysis.

### Genetic makeup of cell type diversity across life history stages

**Fig. 5:**
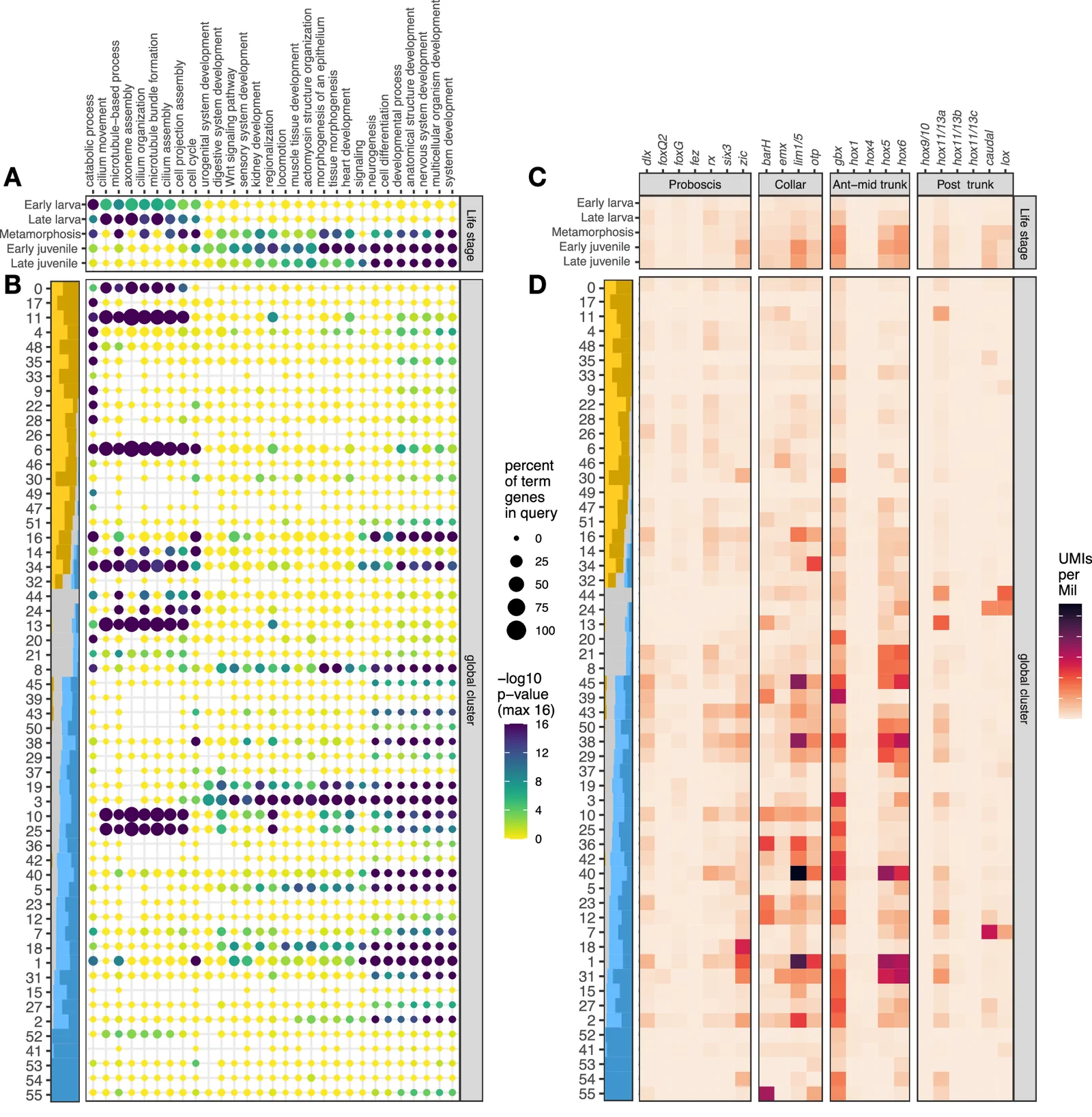
Genetic makeup of cell type diversity across life history stages. **A** GO Term analysis of scRNA-seq differential expression data by life stage. **B** GO Term analysis of scRNA-seq differential expression data by cluster, ordered by cluster life stage composition per Fig. 3B. **C** Analysis of anterior-posterior patterning genes in scRNA-seq data by life stage. **D** Mean expression of anteroposterior patterning genes in scRNA-seq data by cluster, ordered by cluster composition chart from Fig. 3D. *P*-values in dot plots are computed using gProfiler’s custom g:SCS algorithm with included multiple comparison correction.

Next, we sought to understand what kind of genes were driving transcriptional differences between larval and juvenile cell types and how those genes were being regulated. We first used GO term analysis to find classes of genes enriched at different life stages in scRNA-seq differential expression data (Fig. 5A). These results recapitulated our understanding of hemichordate biology: processes such as cilium organization, assembly, and movement were highest in early and late larva, concomitant with the presence of the circumoral ciliary band and the telotroch, as well as during metamorphosis when the circumoral band has degenerated but the telotroch is still retained. In addition, we observed that GO terms were enriched on a cluster-by-cluster basis (Fig. 5B) which also reflected the possible function of these various cell types—for example clusters 6 and 11 were enriched for ciliary components. Ordering the clusters by life stage composition revealed temporal patterns with an enrichment of cellular differentiation and developmental processes starting at metamorphosis. As development proceeds through metamorphosis and into early juvenile and late juvenile stages, we observe an enrichment of GO terms related to morphogenesis like neurogenesis (clusters 16, 1, 2) again reflecting the biological processes occurring at these stages and their underlying cell types (Fig. 5B).

Importantly, we also noted enrichment of GO terms associated with the Wnt signaling pathway during metamorphosis and juvenile stages. It was demonstrated that canonical Wnt signaling in the direct-developing hemichordate *S. kowalevskii* is responsible for activating the Hox complex in the trunk^83^. The anteroposterior transcriptional patterning program has previously been described in *S. californicum*, with the larva being mostly “head” or anterior territory, with the trunk posterior program only being activated at late larval stages and through metamorphosis^29^. We investigated the expression of anteroposterior patterning genes across scRNA-seq and observed specific enrichment of posterior patterning genes in metamorphosis and juvenile stages compared to larval stages (Fig. 5C). More striking, these patterns of gene enrichment also correlated with enrichment of cell types in each life stage (Fig. 5D). Indeed, neural clusters, 38, 40, 1, and 2 that are abundant in early and late juvenile stages also highly express genes of the collar and trunk territories, such as *lim1/5*, *gbx*, *hox5*, and *hox6* (Fig. 5D).

**Fig. 6:**
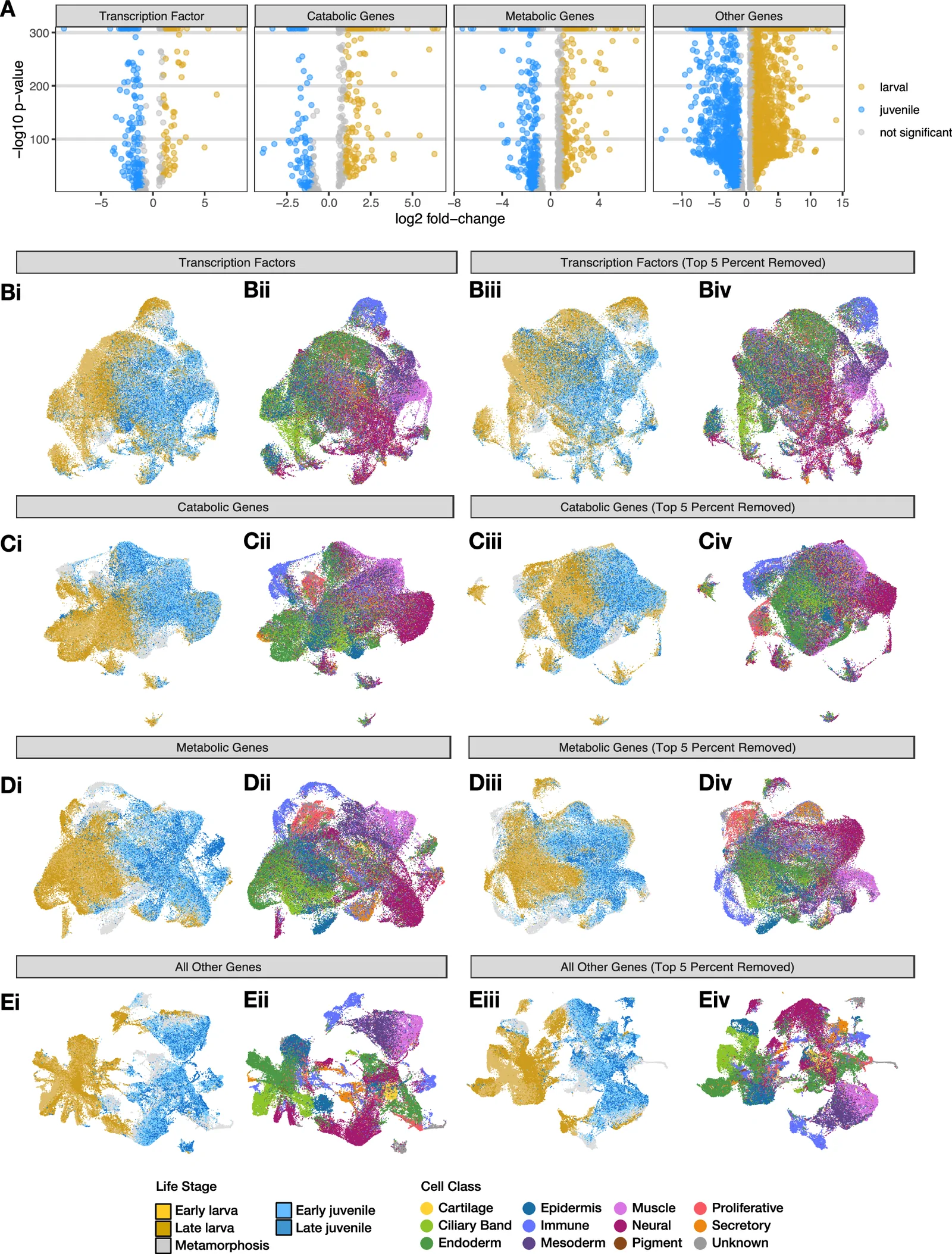
Life stage differences transcend gene type. **A** Volcano plots of differential gene expression between larval and juvenile cells for transcription factors, catabolic genes (GO:0009056), metabolic genes, and all other genes (log2 fold-change versus -log10 Bonferroni-adjusted p-value from Wilcoxon Rank Sum test; points colored by enrichment in larval or juvenile cells, or not significant). **Bi**–**Eiv** UMAP embeddings of the scRNA-seq dataset computed by PCA on restricted gene subsets, colored by life-history stage (panels i and iii) or by broad cell class (panels ii and iv). **Bi**–**Biv** Transcription factors only, full set (**Bi**, **Bii**) and after removal of the top 5% most differentially expressed transcription factors (**Biii**, **Biv**). **Ci**–**Civ** Catabolic genes only, full set (**Ci**, **Cii**) and top 5% removed (**Ciii**, **Civ**). **Di**–**Div** Metabolic genes only, full set (**Di**–**ii**) and top 5% removed (**Diii**–**iv**). **Ei**–**Eiv** All remaining genes (excluding transcription factors, metabolic, and catabolic genes), full set (**Ei**–**ii**) and top 5% removed (**Eiii**–**iv**).

While these patterning genes may reflect the distribution of cell types across life stages, we next asked whether the transcriptional differences between larval and juvenile cell types were disproportionately driven by a small number of highly differentially expressed genes from specific functional classes (Fig. 6A). To address this, we performed principal component (PC) analysis and UMAP embedding on the scRNA-seq dataset using restricted subsets of genes. When analyses were performed using only predicted transcription factors (Fig. 6Bi–ii), cells separated strongly by life-history stage while still recapitulating the major cell-class relationships identified in the full dataset (Fig. 2A). Removal of the top 5% most differentially expressed transcription factors had little effect on either life-stage separation or overall cell-class organization (Fig. 6Biii–iv). Although the transcription factor-only embedding exhibited less sharply resolved structure than analyses using the full transcriptome or non-transcription factor genes, we interpret this cautiously because dimensionality reduction can be sensitive to feature number and variance structure. Nevertheless, the broader organization of the transcription factor-only UMAP may reflect the pleiotropic deployment of regulatory factors across multiple cell classes relative to the more cell-type-restricted expression of terminal differentiation genes.

We next repeated this analysis using catabolic genes (Fig. 6Ci–iv), metabolic genes (Fig. 6Di–iv), and all remaining genes outside these categories (Fig. 6Ei–iv). In each case, cells continued to separate primarily by life-history stage while maintaining the broad cell-class structure observed in the full atlas. Importantly, removal of the top 5% most differentially expressed genes within each category again produced only minor changes to the overall organization of the UMAPs.

Together, these analyses indicate that the transcriptional distinction between larval and juvenile states is not driven by a small number of dominant marker genes or restricted to a single functional class of genes. Instead, the divergence reflects broad, distributed differences across multiple regulatory, metabolic, catabolic, and physiological programs. These findings suggest that life-history stage acts as a major organizing axis of transcriptional identity across conserved functional cell classes, likely reflecting the distinct ecological and physiological demands of a planktonic larva versus a benthic juvenile.

## Discussion

We examined the diversity of cell types in the hemichordate *S. californicum* across five developmental stages: two larval stages, one during metamorphosis, and two juvenile stages. This analysis identified 12 cell classes: cartilage, ciliary band, endoderm, epidermis, immune, mesoderm, muscle, neural, pigment, proliferative, secretory, and unknown. Our findings encompass all major tissue types and include previously unrecognized cell populations.

Some of our findings were unsurprising based on our existing knowledge of hemichordate biology and the loss or gain of cell types associated with loss and gain of structures between life history stages. For example, two ciliary clusters mark the two ciliary bands of the larva, and both the cell clusters and morphological structures are lost following metamorphosis as the benthic adult body plan is formed. Conversely, we observe the appearance of novel clusters associated with the adult body plan, such as the cartilage that is used for structural support of the proboscis and the gill slits.

Our primary objective was to assess how cell type composition changes throughout the organism’s complex life cycle. By characterizing broad cell types, we established new molecular markers to track the distribution of major cell classes across developmental stages.

We considered three explicit hypotheses regarding how cellular composition maps to life history. First, do larval cell types represent a subset of adult cell types, with metamorphosis leading to an expansion of cellular diversity as the organism develops a more complex adult body plan? Second, do larval and adult stages share a substantial portion of their cellular complements, but each stage also possesses unique cell types? Third, are larval and adult body plans composed of distinct cell types, exhibiting minimal overlap in their transcriptional programs?

Surprisingly, our data largely supports the third hypothesis: when life history is overlaid onto the low-dimensional embeddings, there is a marked separation of cell states associated with life history (Fig. 3A), and this separation is borne out by higher-dimensional analysis (Fig. 4B). Larval cells cluster together to define a larval transcriptional state, largely to the exclusion of juvenile states, with metamorphosis representing an intermediate state. In other words, cells cluster primarily by life history stage rather than by cell type identity. For example, larval neurons and larval gut cells are generally more transcriptionally similar to each other than either is to adult neurons or gut, respectively (Fig. 4B, 4Di-ii). This pattern is suggested in UMAP embeddings and confirmed by hierarchical clustering.

Both endoderm and ectoderm-derived cell types follow this stage-specific clustering pattern: larval cells clustering with other larval cells, and adult cells clustering with other adult cells. Mesoderm and muscle are exceptions. These cell types maintain similar transcriptional profiles in both larvae and adults, suggesting that mesoderm undergoes metamorphosis through different mechanisms than the other two germ layers. Several unidentified clusters also exhibited strong life-stage specificity, including populations enriched during metamorphosis or restricted to juvenile stages. These clusters may represent transient developmental states or highly specialized cell populations associated with particular phases of the life cycle.

Interestingly, the transcriptional differences between larval and juvenile cell states are not driven by a small number of stage-specific genes or restricted to a single functional category. Instead, the divergence is broadly distributed across regulatory, metabolic, catabolic, and other physiological gene programs (Fig. 6). We do not observe a pattern in which life-history-specific clustering is dominated by a small subset of highly differentially expressed transcripts. Consistent with this, removal of the top 5% of differentially expressed genes within each functional category had little effect on the overall UMAP structure (Fig. 6Biii–Eiv). This pattern is congruent with previous work in indirect developers proposing that larval and adult body plans are associated with broad territory-level transcriptional states rather than discrete stage-specific master regulators^84^. These results suggest that life-history stage acts as a major organizing axis of transcriptional identity, reshaping broadly conserved functional cell classes across metamorphosis.

### Developmental mechanisms of life history cellular transformation

We propose two hypotheses to explain these observations. First, larval cells may undergo large-scale apoptosis, with adult cells arising from sequestered stem cells. This largely fits with Davidson and colleagues’ “set aside” hypothesis inspired by sea urchin development, where adult structures originate from sequestered progenitor cells^85^ and metamorphosis represents a total cellular turnover between larvae and adults. The transcriptional state discontinuity observed in endodermally and ectodermally derived cells between larval and adult stages (Figs. 3, 4) and the loss of larval ciliary bands at metamorphosis could be consistent with this hypothesis. Additionally, the presence of proliferative cell states identified by irradiation-sensitive genes *lbr-1* and *dlgp5*^39^ and the marker *pif1* could be the source of new cells (Supplementary Fig. 7B, C). However, previous work did not reveal extensive apoptosis during metamorphosis, with TUNEL staining restricted to ciliary band cells at the start of metamorphosis^39^. Furthermore, the carryover of EdU-labeled larval cells through metamorphosis into adulthood that we observed (Fig. 3Gii-Hii) is inconsistent with extensive larval cell turnover between life histories.

In the second hypothesis, larval cells may undergo transcriptional reprogramming during metamorphosis via dedifferentiation and redifferentiation, or via transdifferentiation, which has been postulated to play a key role during adult body plan development by transformation of a larva^86,87^. Lineage and functional studies are needed to test for evidence of reprogramming and to differentiate between the possible alternative developmental explanations. However, evidence for transcriptional reprogramming includes cluster 16 (neural cells), which spans all life stages and exhibits spatial continuity in *pburs* expression. Larval *pburs* localizes to the eyespots in the apical organ (Fig. 3Ei) and during metamorphosis it is carried over into adult apical ectoderm (Fig. 3Eii, Supplementary Fig. 5E), suggesting transcriptional programming changes in retained cells.

In contrast, mesoderm does not follow this pattern. Muscle and mesoderm cells retain similar transcriptional states irrespective of life history, as has also been described in a scRNA-seq comparisons between larvae and adult body plans of two different sea urchin species^21^. Additionally, similar to the findings of Paganos et al. (2025), some immune cells exhibit overlapping transcriptional states or close interstage relationships (clusters 17, 20, Figs. 2A, 3A). Several unidentified clusters also exhibited strong life-stage specificity, particularly during metamorphosis and juvenile development, suggesting the existence of additional transient or lineage-restricted cell states that remain to be characterized (Supplementary Fig. 8). In summary, the cellular composition of the transition from larval to adult body plans in the enteropneust *S. californicum* likely involves transcriptional reprogramming of ectodermal and endodermal cells and conservation of the mesodermal cellular program. Future work combining cellular profiling and functional assays will clarify these mechanisms.

### Life history complexities for the construction of cell atlases

Our data suggests that the striking differences in body plan organization of larvae and adults is primarily based on fundamental differences in cell type composition and transcriptional state. As we continue to build a better understanding of metazoan cell type evolution, it is essential that we consider how to integrate life history complexity into a broader synthesis of animal cell type evolution. Currently, the sampling of bilaterian phyla in comparative cell type studies is mostly limited to direct developers or to early larval stages of animals with complex life cycles.

Much of what we understand of the changes in the cellular composition during metamorphosis comes from seminal work in insects. In holometabolous insects many of the larval tissues undergo programmed cell death, but some larval neurons are carried over and reprogrammed during metamorphosis^88,89^. In sea urchins and nemerteans it has been proposed that most larval cells do not persist into adulthood^90,91^ but this has never been explicitly tested. More broadly in bilaterians, the process of deferred development^92^, such as temporal shifts in development of the trunk programs^13,29^ may help to explain the diversity of animal life cycles. However, how cellular composition changes in relation to these heterochronies has not been explored in detail. Our understanding of the dynamics of cellular composition in complex life cycles requires further analysis.

Our view of cellular differentiation has been heavily influenced by the findings from model species, and in most cases, differentiation in somatic cells is terminal. Some of the earliest branching animals are highly plastic and cell fate changes are very common^93,94^. However, there are also many exceptions to the view that most cells are terminally differentiated – even in vertebrates – and that cellular plasticity is more common than generally acknowledged^87^.

A growing range of single-cell RNA-seq projects are beginning to provide insights into how cell types change between life history stages^19–23^. Our work demonstrates that specific cellular complements have evolved to support the vastly different ecologies and behaviors of larvae and adults, and that larval cells may have stable rather than terminally differentiated cell states^87^. This plasticity may be common in species with a similar life history to *S. californicum* but that needs to be explored in further studies. Ultimately, complex life cycles are phylogenetically broadly dispersed and an essential component of animal biodiversity that should be investigated in more depth and integrated into our understanding of cell type differentiation and evolution.

## Methods

### Collecting, spawning, and larval rearing

Adult *Schizocardium californicum* were collected in Morro Bay State Park, California, in a mudflat located at 35°20′56.7“N 120°50′35.6“W with appropriate state permitting. Animals were spawned with individual females transferred in bowls of filtered seawater (FSW) and placed in an illuminated incubator at 24–26 °C^40^. Once embryos hatched, larvae were transferred to 1 gallon glass jars with continuous stirring and fed with a 1:1 mix of *Dunaliella tertiolecta* and *Rhodomonas lens*. Every two to four days, containers were washed, water was replaced with clean FSW, and fresh algae was added. To grow larger numbers of animals through metamorphosis, some larvae were placed on a continuous flow-through system by being transferred into diffusion tubes^95^. Once animals began metamorphosis, they were transferred into glass bowls with terrarium sand.

### Single-cell sequencing

#### Cell preparation

Early and late juveniles were first diced into fine pieces with a razor blade on a petri dish. All stages were suspended in 1 mg/mL Dispase II (Sigma D4693) in 3.3× PBS, 2% Fetal Calf Serum, and 20 mM HEPES. Cells were pipetted intermittently with Pasteur pipettes passed over a flame to create narrower openings. Cells were then passed through a 100 μm mesh and centrifuged at 4 °C, 150 g, for 5 min with slow braking. Cells were resuspended in fresh media, passed through a 40 μm strainer on ice, and counted on a hemocytometer. Importantly, cells were kept in a 3.3× PBS solution to maintain osmolarity to seawater.

#### Sequencing

Cells were loaded into the 10x Chromium Controller for cell capture and libraries were processed after SPRI size-selection with the 10x Genomics V2.0 workflow. Final libraries were analyzed on an Agilent Bioanalyzer and sequenced using an Illumina Novaseq.

#### Data processing

Once reads were acquired, they were demultiplexed, mapped, and subsequently converted into count matrices using 10x Genomics Cell Ranger 3.0.0^96^. Cell quality filtering, clustering, and dimensionality reduction were done using Seurat v3 in R^97–99^. Cells were filtered to those with at least 500 and less than 57,000 unique molecular identifiers (UMIs) and >90 and no more than 980 genes. We normalized each batch’s gene expression using Seurat’s SCTransform, then combined the batches using reciprocal principal component analysis (rPCA) integration^99^. This technique was chosen both for its efficiency on large datasets, and because rPCA integration by batch resulted in good overlap of the same life stage from different batches without forcing overlap of different life stages. After this, we performed principal component analysis (PCA) on the combined dataset with 55 principal components (PCs), generated a shared nearest neighbor graph, used clustering to identify 56 cell clusters, and determined which genes are most differentially expressed across the dataset.

### Alternative integration approaches

To integrate data sets and test for batch effects, we used the Portal algorithm^72^. Each batch was read in from the Seurat file and integrated incrementally. The datasets were preprocessed and integrated with default parameters and 1000 training steps. We recovered the harmonized expression matrix in log-normalized level. We then processed this expression matrix with the Self-Assembling-Manifold (SAM) algorithm^100^ with default parameters except for without log normalization or filtering genes. Under these parameters, we recovered no significant batch effects.

To integrate data sets and test for batch effects we ran “Harmony” on our principle component embeddings which projects cells into a shared embedding where cells group by cell type rather than dataset-specific conditions^71^. Independently, we took the cell count matrices and used the Portal algorithm where each batch was read in and integrated incrementally^72^. The datasets were preprocessed and integrated with default parameters and 1000 training steps. We recovered the harmonized expression matrix in log-normalized level. We then processed this expression matrix with the Self-Assembling-Manifold (SAM) algorithm^100^ with default parameters except for without log normalization or filtering genes. Under both approaches we recovered distinct differences between life history stage and no significant batch effects.

### GO analysis

We first performed differential gene expression of positive markers as calculated across the relevant groups (cluster, life stage) using Seurat’s FindMarkers on the SCTransform-normalized single-cell expression data. We then filtered these results to genes with (1) average log2 fold-change of at least 1.25; (2) adjusted p-value less than 0.0001; (3) which had a reciprocal best BLASTp hit between *Schizocardium californicum* and *Mus musculus* GRCm39 predicted peptide sequences. These differential expression results were then sorted by log2 fold-change and fed into gprofiler2’s gost function with organism = “mmusculus” and ordered_query=TRUE^101^. GO Terms were manually curated for plotting.

### High-dimensional analysis

To perform high-dimensional analysis of overall transcriptional similarity of clusters and cell types, the centroid of each cluster was calculated in 55-dimensional PC space, inter-cluster distances were found for all pairs of clusters, and these distances were scaled as the percentage of the largest inter-cluster distance. These distances were then used to build a hierarchical clustering of all clusters in the dataset. For more detailed analysis, cells were grouped by the combination of cell type and life stage, group centroids and inter-group distances calculated, and these distances scaled by the same factor as the inter-cluster distances to facilitate direct comparison. Then the distribution of distances from each group’s individual cells to each group centroid was determined to assess the accuracy of inter-centroid distances. Additionally, we implement a neighbor-joining approach^102^ and recovered relationships broadly consistent with our centroid-based analyses, supporting the robustness of the observed cell-type associations across life-history stages (Supplementary Fig. 10).

### Ortholog identification

Gene orthology and naming were determined by collecting sequences of interest from related species and then building gene trees. Sequences were aligned with MUSCLE^103^ and trees were calculated with Bayesian inference trees using MrBayes version 3.1.2^104^ in 1,000,000 generations with sampling of trees every 100 generations and a burn-in period of 25%.

### In situ **HCR**

#### Probe design and preparation

Complementary DNA sequences specific to genes of interest were submitted to the in situ probe generator from the Ozpolat Lab^105^. The sequences generated by the software were used to order DNA oligo pools (50 μmol DNA oPools™ Oligo Pools) from Integrated DNA Technologies, resuspended to 1 μmol/μL in 50 mM Tris buffer, pH 7.5. HCR amplifiers with fluorophores B1-Alexa Fluor-546, B2-Alexa Fluor-488, and B3-Alexa Fluor-647 were ordered from Molecular Instruments, Inc.

#### HCR protocol

HCRs were performed based on Choi et al., 2018 and the Hybridization Chain Reaction (HCR) in situ Protocol from the Patel Lab^106,107^. Animals were fixed with 3.7% paraformaldehyde in MOPS fix buffer (0.1 M MOPS, 0.5 M NaCl, 2 mM EGTA, 1 mM MgCl2, 1× PBS) overnight at 4 °C. Fixed samples were then dehydrated in methanol and stored at −20 °C. Samples were rehydrated in a gradual series of PBS containing 0.1% Tween-20 (PBST). They were permeabilized in detergent solution (1.0% SDS, 0.5% Tween-20, 150 mM NaCl, 1 mM EDTA (pH 8), 50 mM Tris-HCl at pH 7.5) for 30 min. The samples were then extensively washed in PBST, before being pre-hybridized in hybridization buffer (Molecular Instruments) for 1 h at 37 °C. The probes were then added to the hybridization buffer at a final concentration of 0.05 μM and the samples were allowed to hybridize at 37 °C overnight under gentle agitation. Following hybridization, the samples were washed 4 times for 15 minutes in probe wash buffer (Molecular Instruments) at 37 °C and then in 5× SSCT at room temperature. They were then pre-amplified in amplification buffer (Molecular Instruments) for 30 min. Amplifiers were separately incubated at 95 °C for 90 s and then pooled together before being added to the amplification buffer at a final concentration of 60 nM. Amplification was performed overnight, and the samples were subsequently washed 4 times for 30 min in 5× SSCT and incubated in 5× SSCT containing 1:1000 Hoechst overnight.

### Pulse-chase EdU labeling

Labeling and detection of proliferating cells were performed using the Click-iT Plus EdU 488 Imaging Kit (Life Technologies), using the standard protocol with the following modifications. Larvae were cultured in FSW supplemented with 10 μM EdU diluted from a 10 mM stock in DMSO. Animals were pulsed for 48 hours with EdU then either fixed with 3.7% paraformaldehyde in MOPS fix buffer (0.1 M MOPS, 0.5 M NaCl, 2 mM EGTA, 1 mM MgCl2, 1× PBS) overnight at 4 °C or transferred to fresh FSW and allowed to grow and were fixed at either 43 or 26 days later when they had completed metamorphosis. For detection of EdU incorporation, labeled tissue was transferred to a solution of PBS and the detection was performed following the manufacturer’s protocol with an increased permeabilization time in 0.5% Triton X-100 in PBS of 40 min and an increased detection time of 45 min.

### Imaging

Samples were mounted in PBS (larva) or fructose (juveniles) between two glass coverslips raised slightly with small clay feet. Imaging was performed on a Zeiss LSM 700. For large samples, stacks were stitched together using the Tile tool (Zen2 software, Zeiss).

### Reporting summary

Further information on research design is available in the Nature Portfolio Reporting Summary linked to this article.

## Supplementary information

Supplementary Information

Peer Review file

Description of Additional Supplementary Files

Supplementary Data 1

Supplementary Data 2

Supplementary Data 3

Supplementary Data 4

Reporting Summary

## Data Availability

The sequencing raw reads and data generated in this study have been deposited in the NCBI BioProject database under the accession code PRJNA1297089.

## Code availability

Analysis code supporting this study is available at: https://doi.org/10.5281/zenodo.21799035.
