# Supplementary material for: Distinct cell states define larval and adult body plans in a hemichordate
Source: Nat Commun. 2026 Sep 8;17:9358. doi: 10.1038/s41467-026-77191-y (PMC13554239; doi:10.1038/s41467-026-77191-y)
Supplement: Supplementary file 1 [file 41467_2026_77191_MOESM1_ESM.pdf]

Supplementary Figure 1

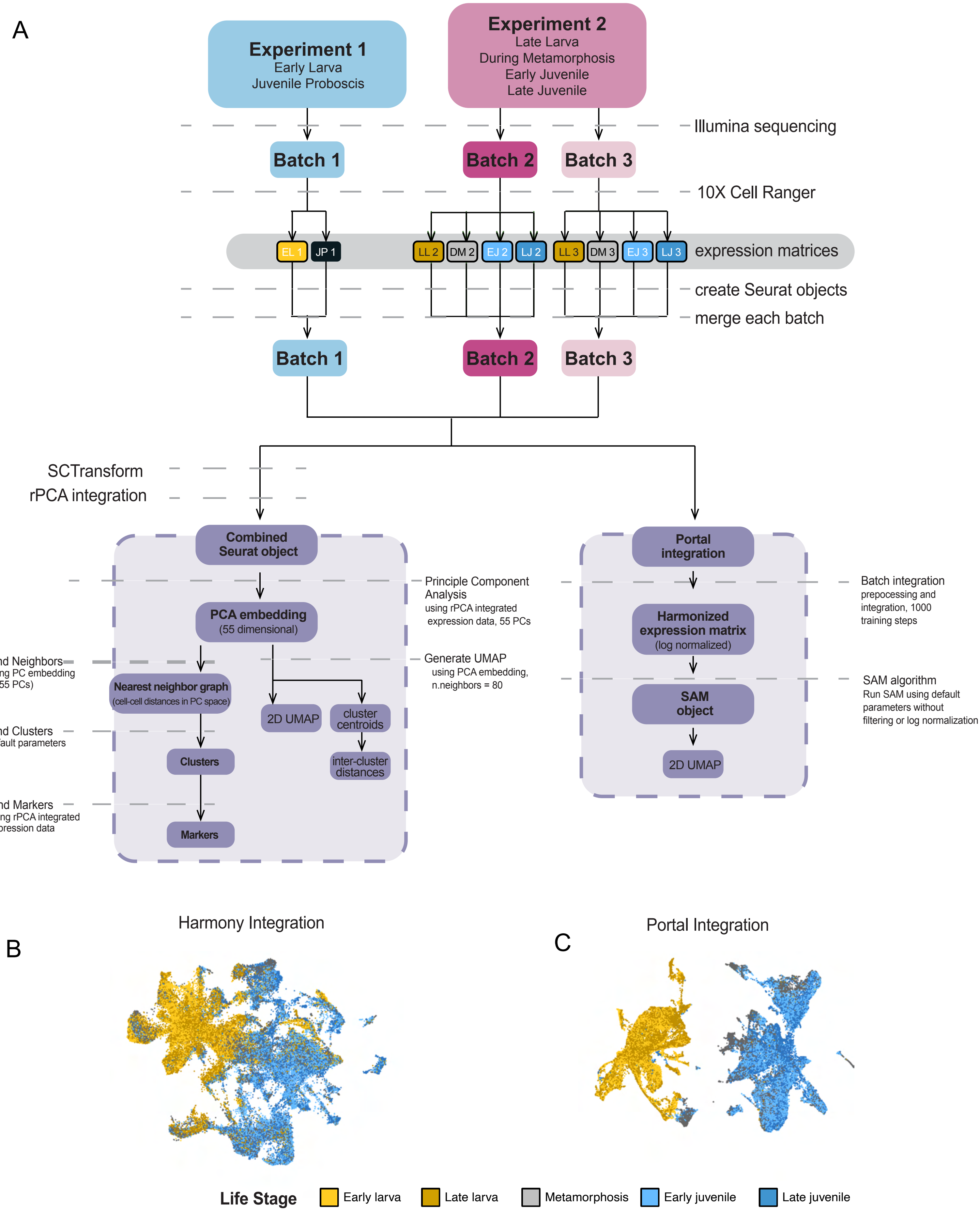

Supplementary Figure 1. Experimental design and alternative integration. A) Flowchart of single-cell analysis including alternative integration approaches. B, C) UMAP embedding of alternative integration approaches, “Harmony” and “Portal” colored by life stage.

Supplementary Figure 2

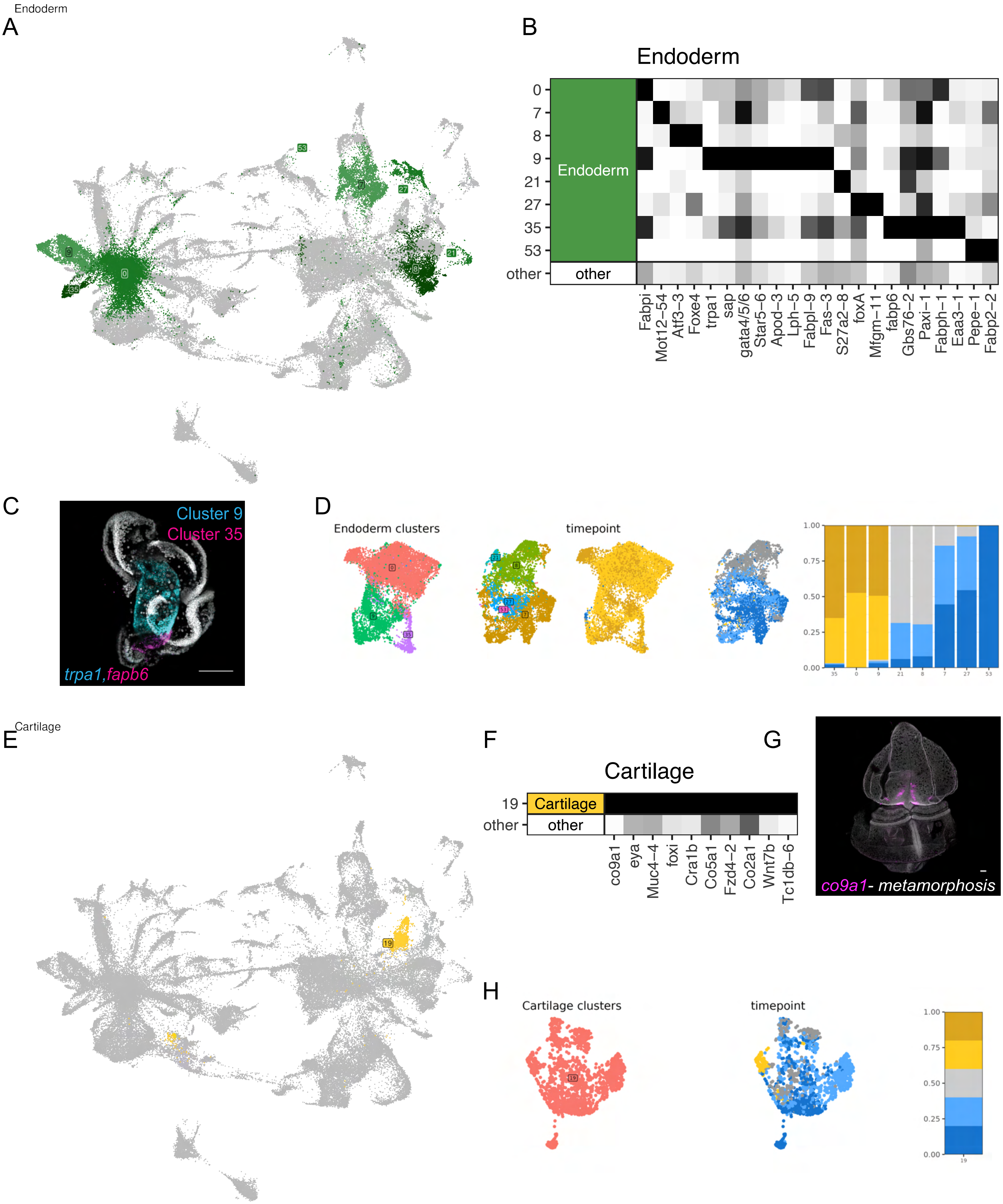

Supplementary Figure 2. Endoderm and cartilage clusters. A) Endoderm clusters (0, 7, 8, 9, 21, 27, 35, 53) plotted in global UMAP embedding. B) Endoderm cluster expression of marker genes. D) Endoderm clusters labeled by cluster, timepoint, and bar chart of life stage composition per Figure 3B. C) HCR expression in early larva of *trpa1* in cyan, *fabp6* in magenta, nuclei in grey, scale bars are 100 µm. E) Cartilage cluster (19) plotted in global UMAP embedding. F) Cartilage cluster expression of marker genes. G) HCR expression during metamorphosis of *co9a1* in magenta, nuclei grey, scale bar 100 µm. H) Cartilage cluster labeled by cluster, timepoint, and bar chart of life stage composition per Figure 3B.

Supplementary Figure 3

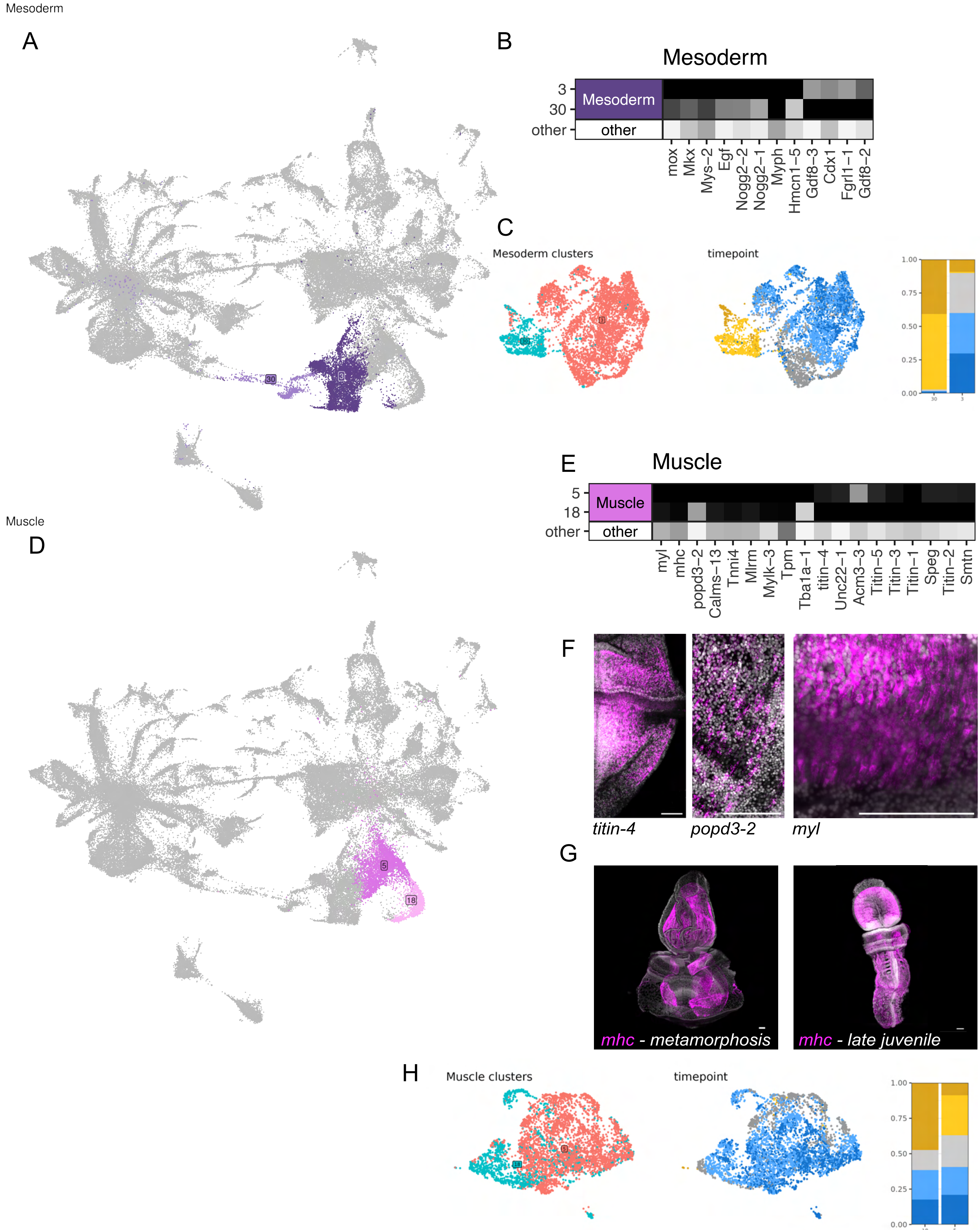

Supplementary Figure 3. Mesoderm clusters. A) Mesoderm clusters (3, 30) plotted in global UMAP embedding. B) Mesoderm cluster expression of marker genes. C) Mesoderm clusters labeled by cluster, timepoint, and bar chart of life stage composition per Figure 3B. D) Muscle clusters (5, 18) plotted in global UMAP embedding. E) Muscle cluster expression of marker genes. F) HCR expression of muscle markers *titin-4, popd3-2, myl1* in magenta, nuclei in grey, scale bars are 100 µm. G) HCR expression during metamorphosis and in late juvenile of *mhc* in magenta, nuclei in grey, scale bars are 100 µm. H) Muscle clusters labeled by cluster, timepoint, and bar chart of life stage composition per Figure 3B.

Supplementary Figure 4

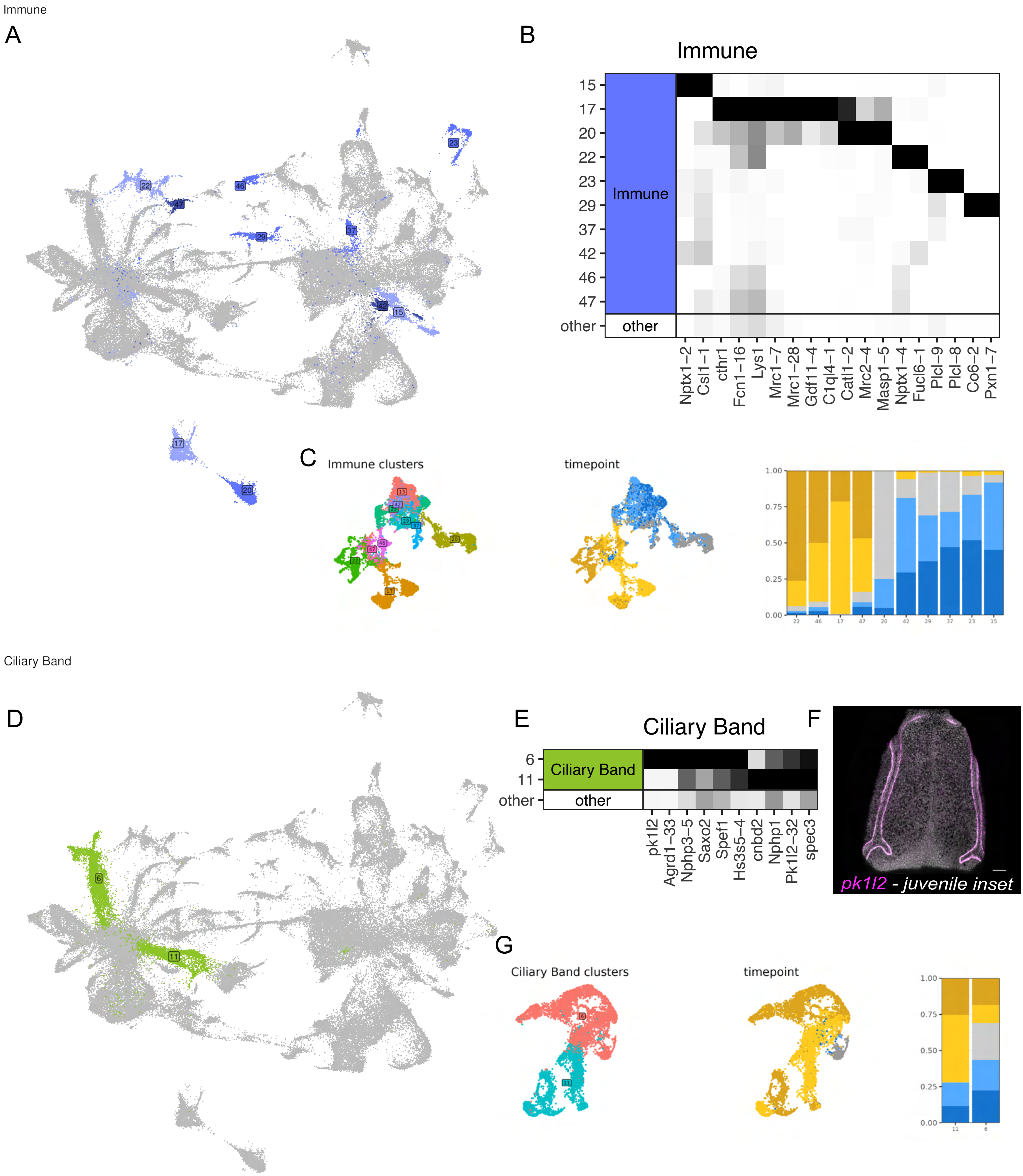

Supplementary Figure 4. Immune and ciliary clusters. A) Immune clusters (15, 17, 20, 22, 23, 29, 37, 42, 46, 47) plotted in global UMAP embedding. B) Immune cluster expression of marker genes. C) Immune clusters labeled by cluster, timepoint, and bar chart of life stage composition per Figure 3B. D) Ciliary clusters (6, 11) plotted in global UMAP embedding. E) Ciliary cluster expression of marker genes. F) HCR expression in early juvenile proboscis inset of *pk1l2* in magenta, nuclei in grey, scale bars are 100 µm. G) Ciliary clusters labeled by cluster, timepoint, and bar chart of life stage composition per Figure 3B.

Supplementary Figure 5

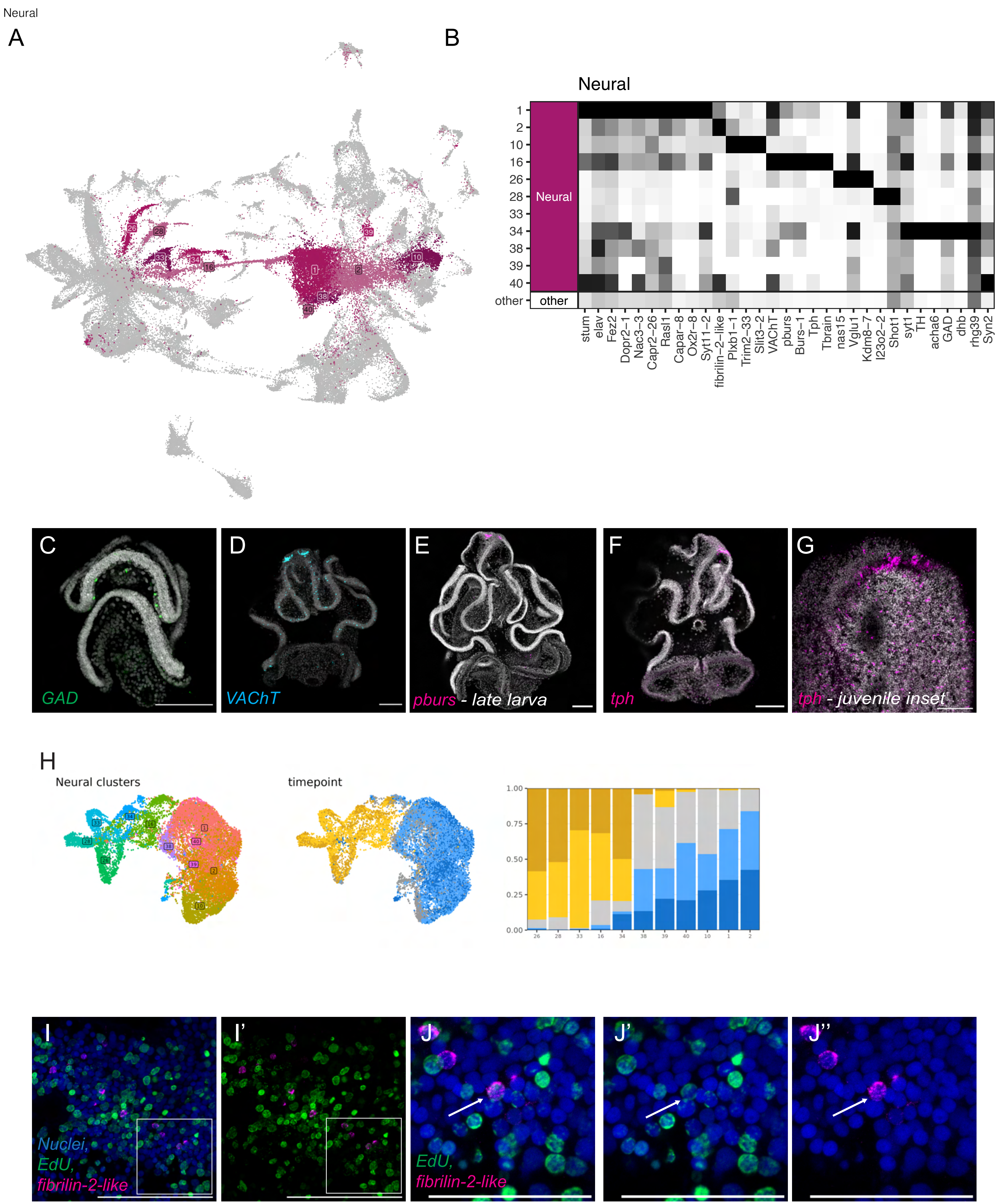

Supplementary Figure 5. Neural clusters. A) Neural clusters (1, 2, 10, 16, 26, 28, 33, 34, 38, 39, 40) plotted in global UMAP embedding. B) Neural cluster expression of marker genes, importantly some markers are broadly expressed across many clusters including *elav, shot1, and rhg39*. C) HCR expression of neural marker *GAD* (green). D) HCR expression of neural marker *VAChT* (cyan). E) HCR expression in late larva of *pburs* (magenta). F, G) HCR expression of *tph* (magenta) in larva (F) and juvenile proboscis (G). Nuclei in grey, scale bars are 100 µm. H) Neural clusters labeled by cluster, timepoint, and bar chart of life stage composition per Figure 3B. I) Pulse-chase chase experiment where late larvae were incubated with EdU, allowed to metamorphose, and then EdU positive cells were detected along with HCR expression of the juvenile neural marker *fibrilin-2-like*. J) Inset of I) with white arrows denoting a specific cell that is positive for EdU and *fibrilin-2-like*. Nuclei in blue, scale bars are 100 µm.

Supplementary Figure 6

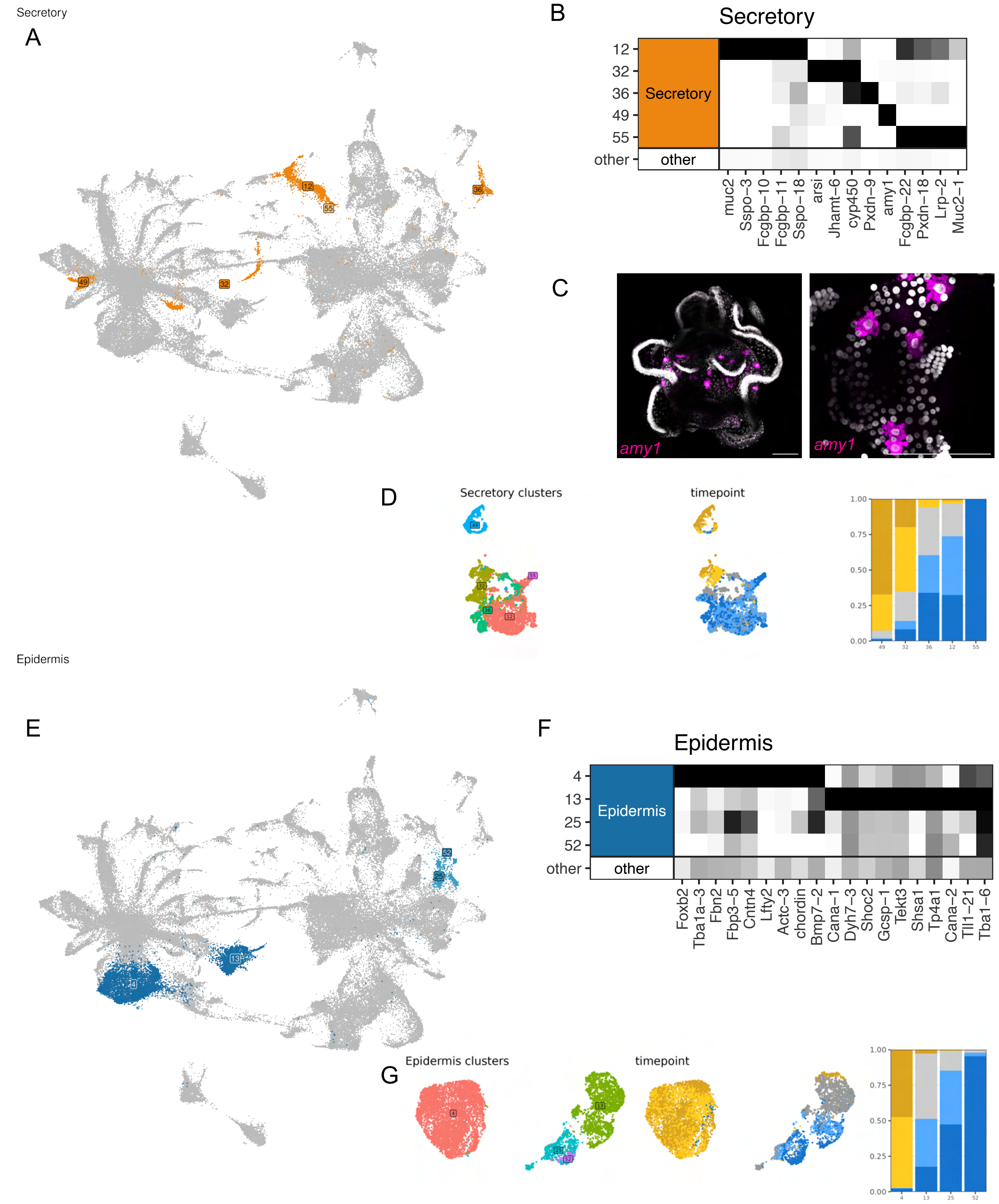

Supplementary Figure 6. Secretory and epidermis clusters. A) Secretory clusters (12, 32, 36, 49, 55) plotted in global UMAP embedding. B) Secretory cluster expression of marker genes. C) HCR expression of secretory marker *amy1* (magenta). D) Secretory clusters labeled by cluster, timepoint, and bar chart of life stage composition per Figure 3B. E) Epidermis clusters (4, 13, 25, 52) plotted in global UMAP embedding. F) Epidermis cluster expression of marker genes. G) Epidermis clusters labeled by cluster, timepoint, and bar chart of life stage composition per Figure 3B.

Supplementary Figure 7

Proliferative

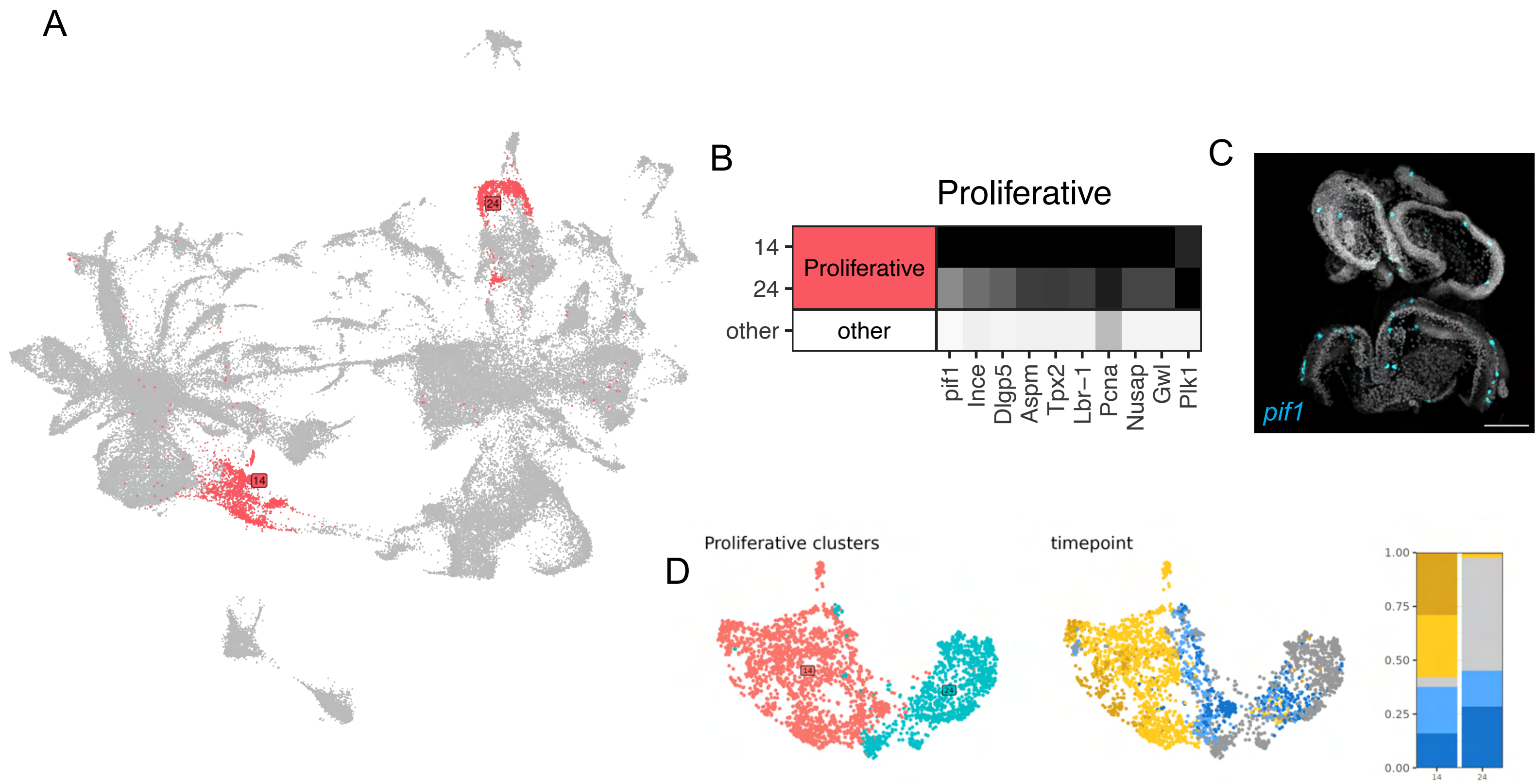

Pigment

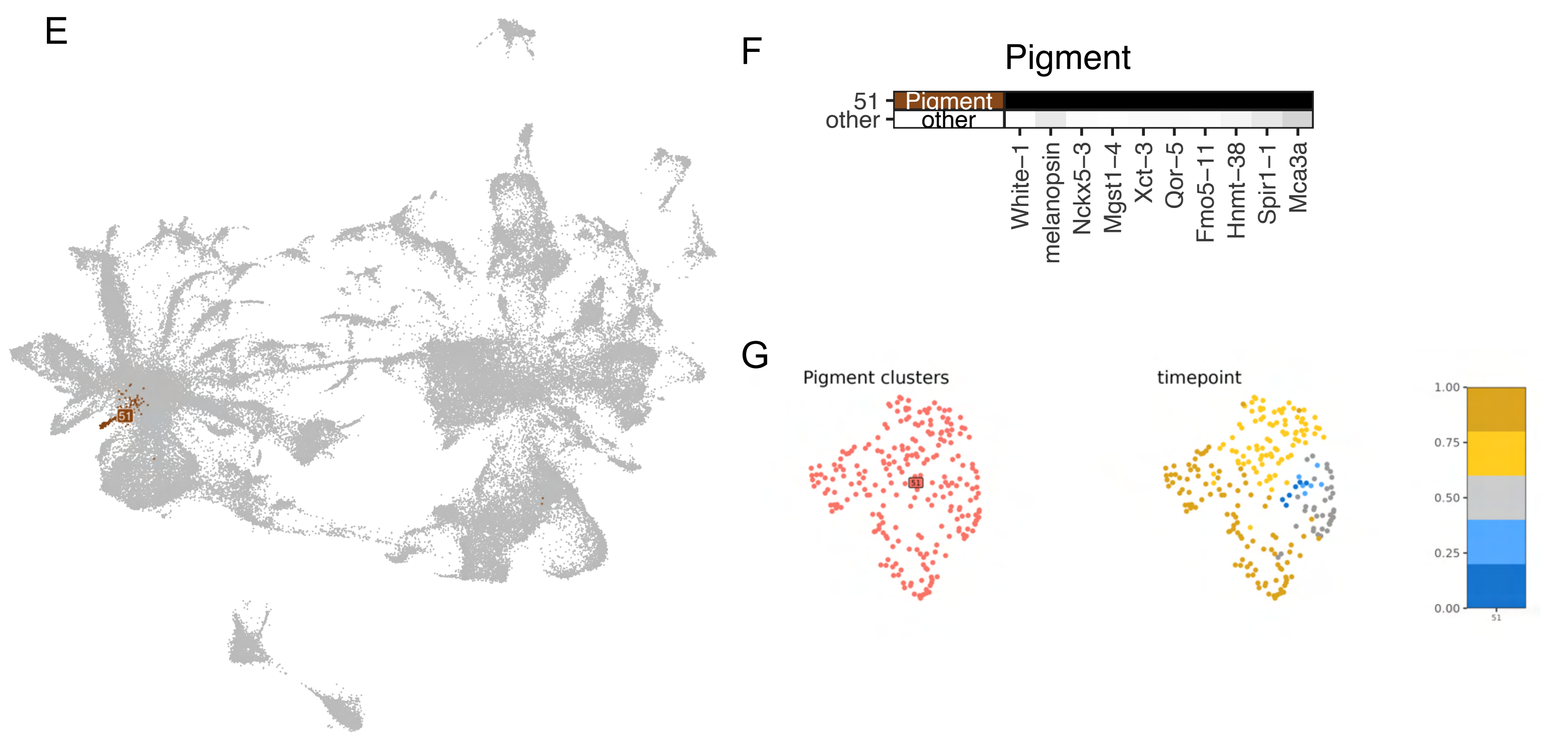

Supplemental Figure 7. Proliferation and pigment clusters. A) Proliferative clusters (14, 24) plotted in global UMAP embedding. B) Proliferative cluster expression of marker genes. C) HCR expression in early larva of *pif1* in cyan, nuclei in grey, scale bars are 100 µm. D) Proliferative clusters labeled by cluster, timepoint, and bar chart of life stage composition per Figure 3B. E) Pigment cluster (51) plotted in global UMAP embedding. F) Pigment cluster expression of marker genes. G) Pigment clusters labeled by cluster, timepoint, and bar chart of life stage composition per Figure 3B.

Supplemental Figure 8

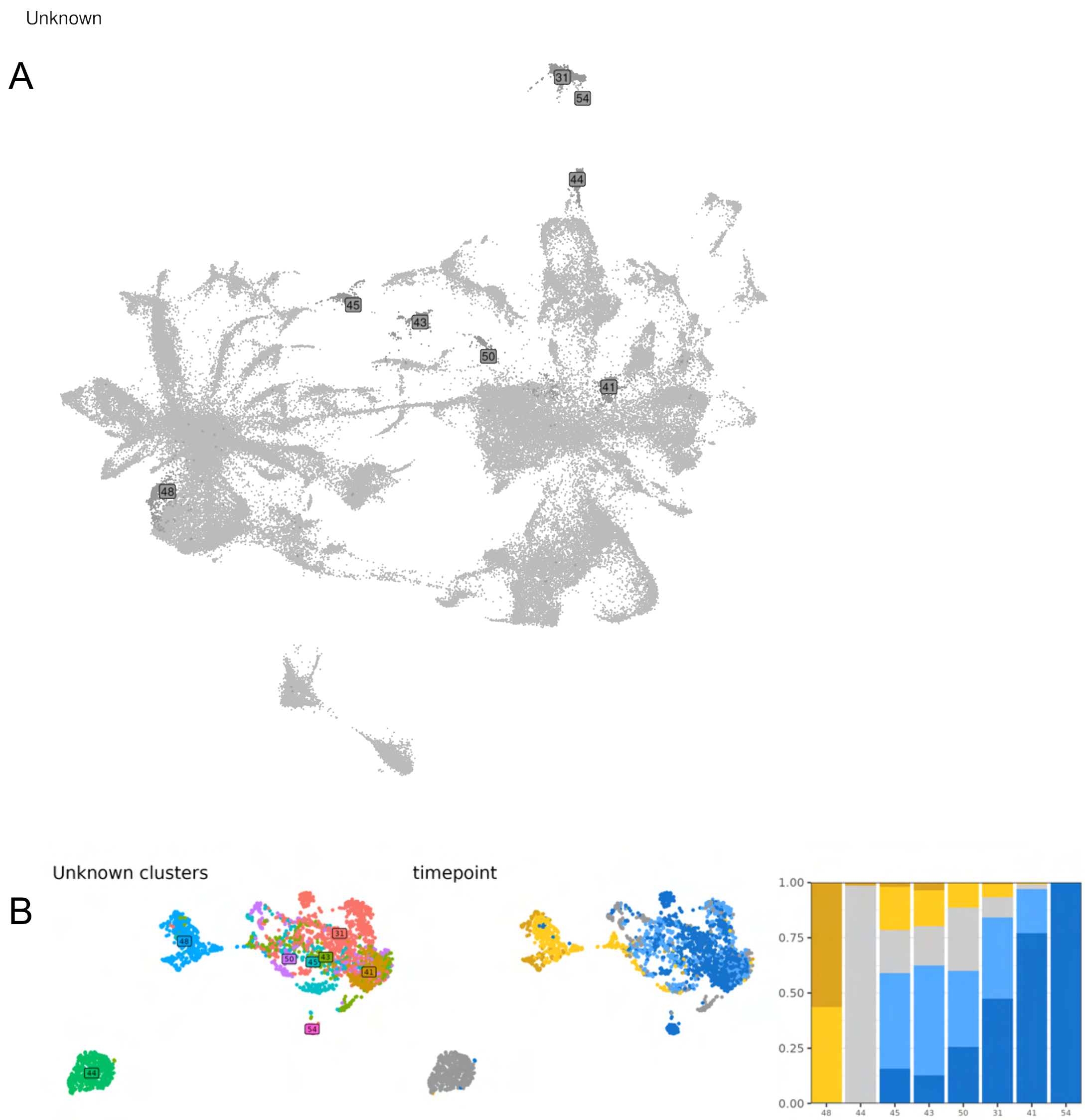

Supplemental Figure 8. Unknown clusters. A) Unknown clusters (31,41,43,44,45,48,50, 54) plotted in global UMAP embedding. B) Unknown clusters labeled by cluster, timepoint, and bar chart of life stage composition per Figure 3B.

Supplemental Figure 9

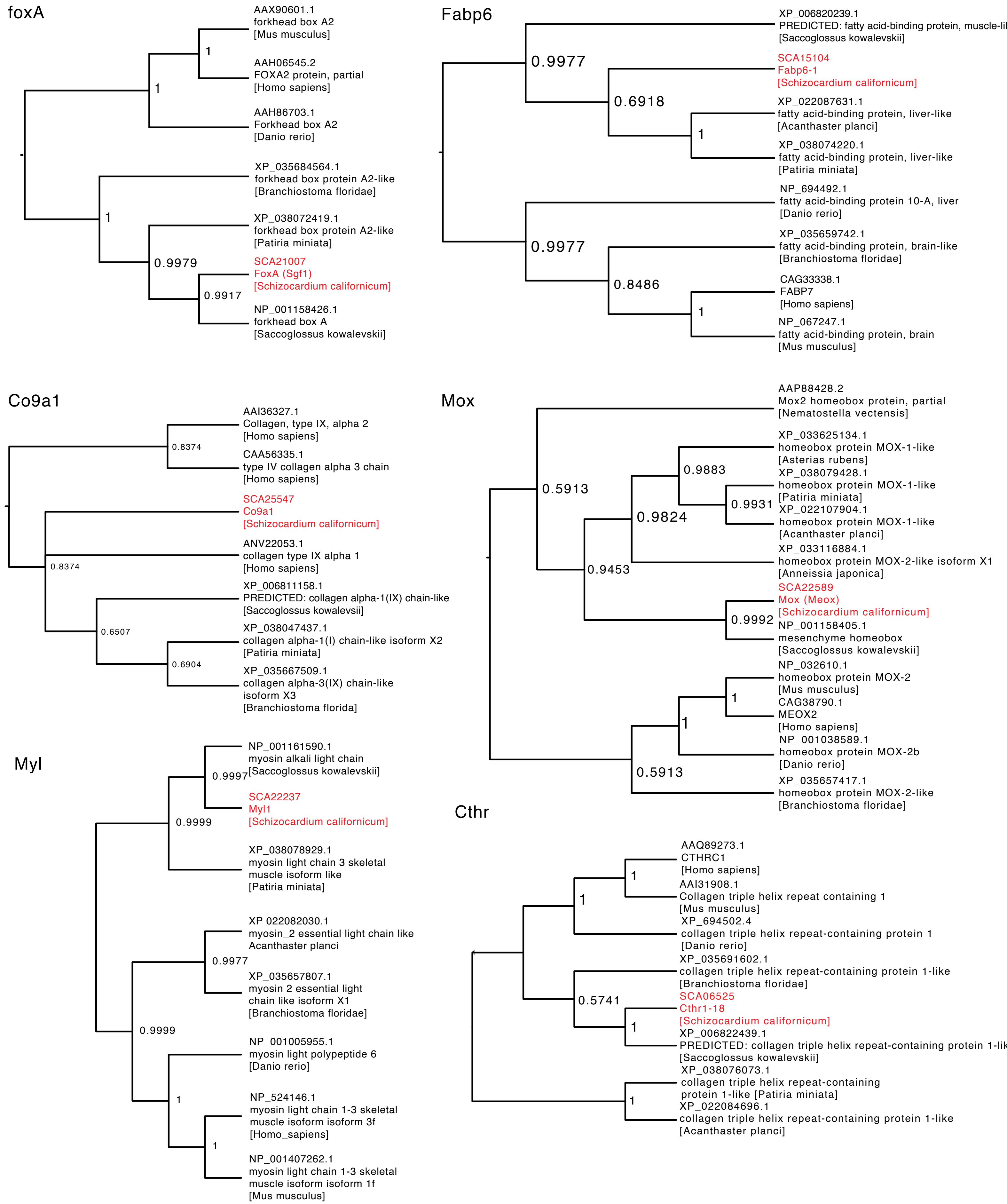

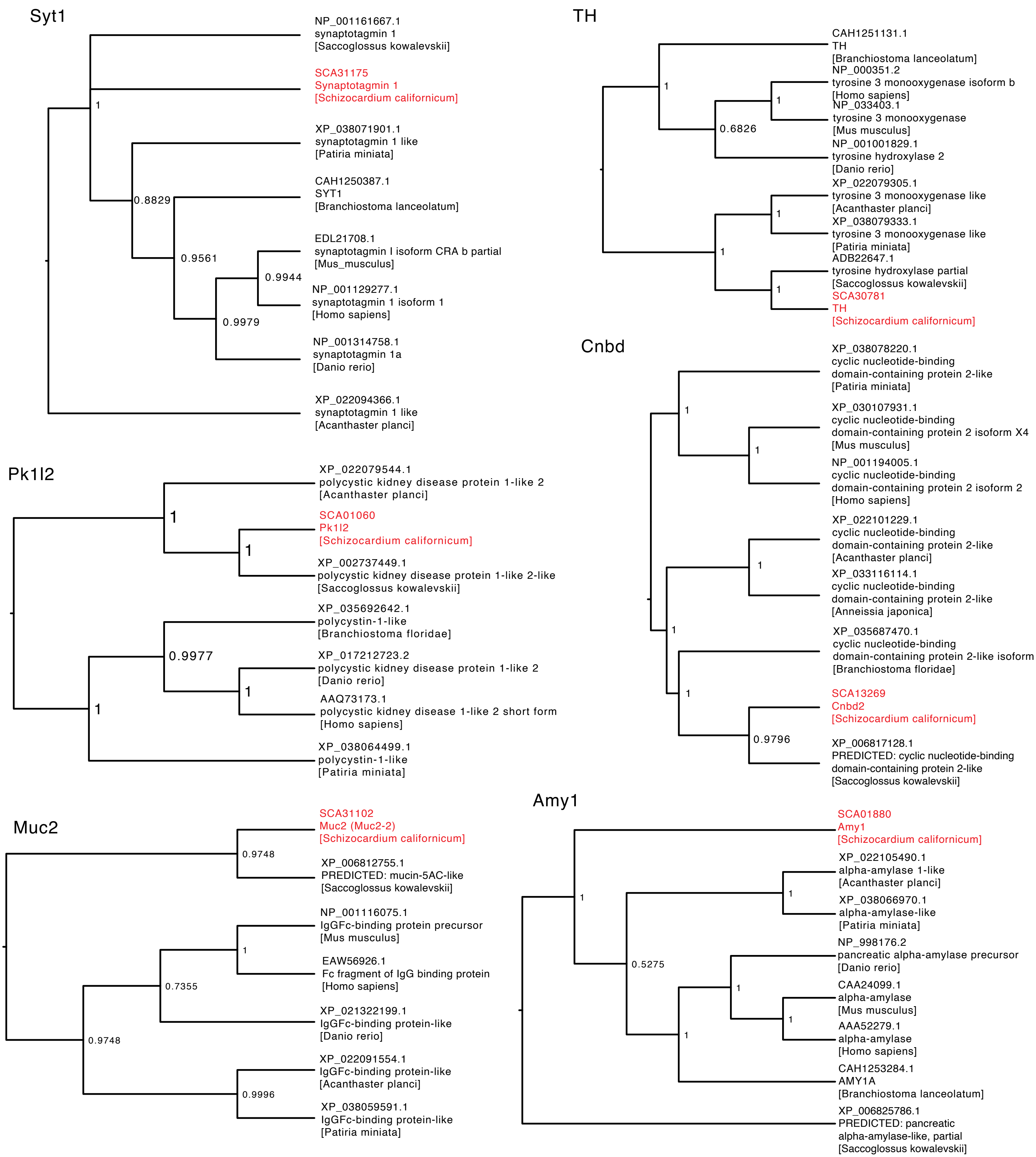
Syt1
NP_001161667.1
synaptotagmin 1
[Saccoglossus kowalevskii]
SCA31175
Synaptotagmin 1
[Schizocardium californicum]
1
XP_038071901.1
synaptotagmin 1 like
[Patiria miniata]
CAH1250387.1
SYT1
[Branchiostoma lanceolatum]
0.8829
EDL21708.1
synaptotagmin I isoform CRA b partial
[Mus_musculus]
0.9561
0.9944
NP_001129277.1
synaptotagmin 1 isoform 1
[Homo sapiens]
0.9979
NP_001314758.1
synaptotagmin 1a
[Danio rerio]
XP_022094366.1
synaptotagmin 1 like
[Acanthaster planci]
TH
CAH1251131.1
TH
[Branchiostoma lanceolatum]
NP_000351.2
tyrosine 3 monooxygenase isoform b
[Homo sapiens]
1
1
NP_033403.1
tyrosine 3 monooxygenase
[Mus musculus]
0.6826
NP_001001829.1
tyrosine hydroxylase 2
[Danio rerio]
XP_022079305.1
tyrosine 3 monooxygenase like
[Acanthaster planci]
1
XP_038079333.1
tyrosine 3 monooxygenase like
[Patiria miniata]
1
ADB22647.1
tyrosine hydroxylase partial
[Saccoglossus kowalevskii]
1
SCA30781
TH
[Schizocardium californicum]
Cnbd
XP_038078220.1
cyclic nucleotide-binding
domain-containing protein 2-like
[Patiria miniata]
1
XP_030107931.1
cyclic nucleotide-binding
domain-containing protein 2 isoform X4
[Mus musculus]
1
NP_001194005.1
cyclic nucleotide-binding
domain-containing protein 2 isoform 2
[Homo sapiens]
XP_022101229.1
cyclic nucleotide-binding
domain-containing protein 2-like
[Acanthaster planci]
1
XP_033116114.1
cyclic nucleotide-binding
domain-containing protein 2-like
[Anneissia japonica]
1
XP_035687470.1
cyclic nucleotide-binding
domain-containing protein 2-like isoform
[Branchiostoma floridae]
1
SCA13269
Cnbd2
[Schizocardium californicum]
0.9796
XP_006817128.1
PREDICTED: cyclic nucleotide-binding
domain-containing protein 2-like
[Saccoglossus kowalevskii]
Pk1l2
XP_022079544.1
polycystic kidney disease protein 1-like 2
[Acanthaster planci]
1
SCA01060
Pk1l2
[Schizocardium californicum]
1
XP_002737449.1
polycystic kidney disease protein 1-like 2-like
[Saccoglossus kowalevskii]
XP_035692642.1
polycystin-1-like
[Branchiostoma floridae]
0.9977
XP_017212723.2
polycystic kidney disease protein 1-like 2
[Danio rerio]
1
AAQ73173.1
polycystic kidney disease 1-like 2 short form
[Homo sapiens]
1
XP_038064499.1
polycystin-1-like
[Patiria miniata]
Muc2
SCA31102
Muc2 (Muc2-2)
[Schizocardium californicum]
0.9748
XP_006812755.1
PREDICTED: mucin-5AC-like
[Saccoglossus kowalevskii]
NP_001116075.1
IgGFc-binding protein precursor
[Mus musculus]
1
EAW56926.1
Fc fragment of IgG binding protein
[Homo sapiens]
0.7355
XP_021322199.1
IgGFc-binding protein-like
[Danio rerio]
0.9748
XP_022091554.1
IgGFc-binding protein-like
[Acanthaster planci]
0.9996
XP_038059591.1
IgGFc-binding protein-like
[Patiria miniata]
Amy1
SCA01880
Amy1
[Schizocardium californicum]
XP_022105490.1
alpha-amylase 1-like
[Acanthaster planci]
1
1
XP_038066970.1
alpha-amylase-like
[Patiria miniata]
0.5275
NP_998176.2
pancreatic alpha-amylase precursor
[Danio rerio]
1
CAA24099.1
alpha-amylase
[Mus musculus]
1
AAA52279.1
alpha-amylase
[Homo sapiens]
1
CAH1253284.1
AMY1A
[Branchiostoma lanceolatum]
XP_006825786.1
PREDICTED: pancreatic
alpha-amylase-like, partial
[Saccoglossus kowalevskii]

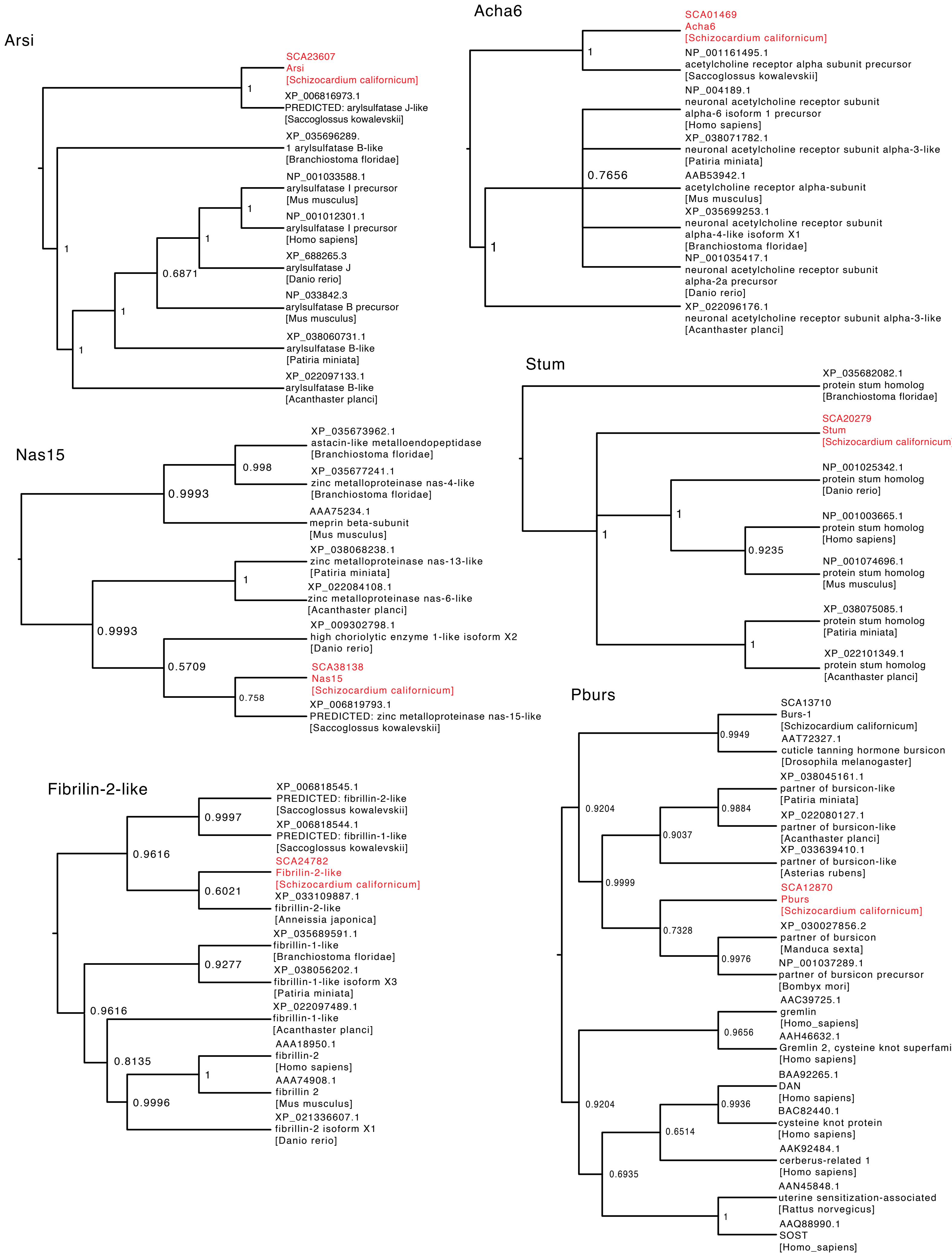
Arsi
SCA23607
Arsi
[Schizocardium californicum]
1
XP_006816973.1
PREDICTED: arylsulfatase J-like
[Saccoglossus kowalevskii]
XP_035696289.
1 arylsulfatase B-like
[Branchiostoma floridae]
NP_001033588.1
arylsulfatase I precursor
[Mus musculus]
1
NP_001012301.1
arylsulfatase I precursor
[Homo sapiens]
1
XP_688265.3
arylsulfatase J
[Danio rerio]
0.6871
NP_033842.3
arylsulfatase B precursor
[Mus musculus]
1
XP_038060731.1
arylsulfatase B-like
[Patiria miniata]
1
1
XP_022097133.1
arylsulfatase B-like
[Acanthaster planci]
Acha6
SCA01469
Acha6
[Schizocardium californicum]
1
NP_001161495.1
acetylcholine receptor alpha subunit precursor
[Saccoglossus kowalevskii]
NP_004189.1
neuronal acetylcholine receptor subunit
alpha-6 isoform 1 precursor
[Homo sapiens]
XP_038071782.1
neuronal acetylcholine receptor subunit alpha-3-like
[Patiria miniata]
0.7656
AAB53942.1
acetylcholine receptor alpha-subunit
[Mus musculus]
XP_035699253.1
neuronal acetylcholine receptor subunit
alpha-4-like isoform X1
[Branchiostoma floridae]
1
NP_001035417.1
neuronal acetylcholine receptor subunit
alpha-2a precursor
[Danio rerio]
XP_022096176.1
neuronal acetylcholine receptor subunit alpha-3-like
[Acanthaster planci]
Stum
XP_035682082.1
protein stum homolog
[Branchiostoma floridae]
SCA20279
Stum
[Schizocardium californicum]
NP_001025342.1
protein stum homolog
[Danio rerio]
1
NP_001003665.1
protein stum homolog
[Homo sapiens]
1
0.9235
NP_001074696.1
protein stum homolog
[Mus musculus]
XP_038075085.1
protein stum homolog
[Patiria miniata]
1
XP_022101349.1
protein stum homolog
[Acanthaster planci]
Nas15
XP_035673962.1
astacin-like metalloendopeptidase
[Branchiostoma floridae]
0.998
XP_035677241.1
zinc metalloproteinase nas-4-like
[Branchiostoma floridae]
0.9993
AAA75234.1
meprin beta-subunit
[Mus musculus]
XP_038068238.1
zinc metalloproteinase nas-13-like
[Patiria miniata]
1
XP_022084108.1
zinc metalloproteinase nas-6-like
[Acanthaster planci]
0.9993
XP_009302798.1
high choriolytic enzyme 1-like isoform X2
[Danio rerio]
0.5709
SCA38138
Nas15
[Schizocardium californicum]
0.758
XP_006819793.1
PREDICTED: zinc metalloproteinase nas-15-like
[Saccoglossus kowalevskii]
Fibrilin-2-like
XP_006818545.1
PREDICTED: fibrillin-2-like
[Saccoglossus kowalevskii]
0.9997
XP_006818544.1
PREDICTED: fibrillin-1-like
[Saccoglossus kowalevskii]
0.9616
SCA24782
Fibrilin-2-like
[Schizocardium californicum]
0.6021
XP_033109887.1
fibrillin-2-like
[Anneissia japonica]
XP_035689591.1
fibrillin-1-like
[Branchiostoma floridae]
0.9277
XP_038056202.1
fibrillin-1-like isoform X3
[Patiria miniata]
0.9616
XP_022097489.1
fibrillin-1-like
[Acanthaster planci]
0.8135
AAA18950.1
fibrillin-2
[Homo sapiens]
1
AAA74908.1
fibrillin 2
[Mus musculus]
0.9996
XP_021336607.1
fibrillin-2 isoform X1
[Danio rerio]
Pburs
SCA13710
Burs-1
[Schizocardium californicum]
0.9949
AAT72327.1
cuticle tanning hormone bursicon
[Drosophila melanogaster]
XP_038045161.1
partner of bursicon-like
[Patiria miniata]
0.9884
XP_022080127.1
partner of bursicon-like
[Acanthaster planci]
0.9204
0.9037
XP_033639410.1
partner of bursicon-like
[Asterias rubens]
0.9999
SCA12870
Pburs
[Schizocardium californicum]
0.7328
XP_030027856.2
partner of bursicon
[Manduca sexta]
0.9976
NP_001037289.1
partner of bursicon precursor
[Bombyx mori]
AAC39725.1
gremlin
[Homo_sapiens]
0.9656
AAH46632.1
Gremlin 2, cysteine knot superfami
[Homo sapiens]
BAA92265.1
DAN
[Homo sapiens]
0.9936
BAC82440.1
cysteine knot protein
[Homo sapiens]
0.9204
0.6514
AAK92484.1
cerberus-related 1
[Homo sapiens]
0.6935
AAN45848.1
uterine sensitization-associated
[Rattus norvegicus]
1
AAQ88990.1
SOST
[Homo_sapiens]

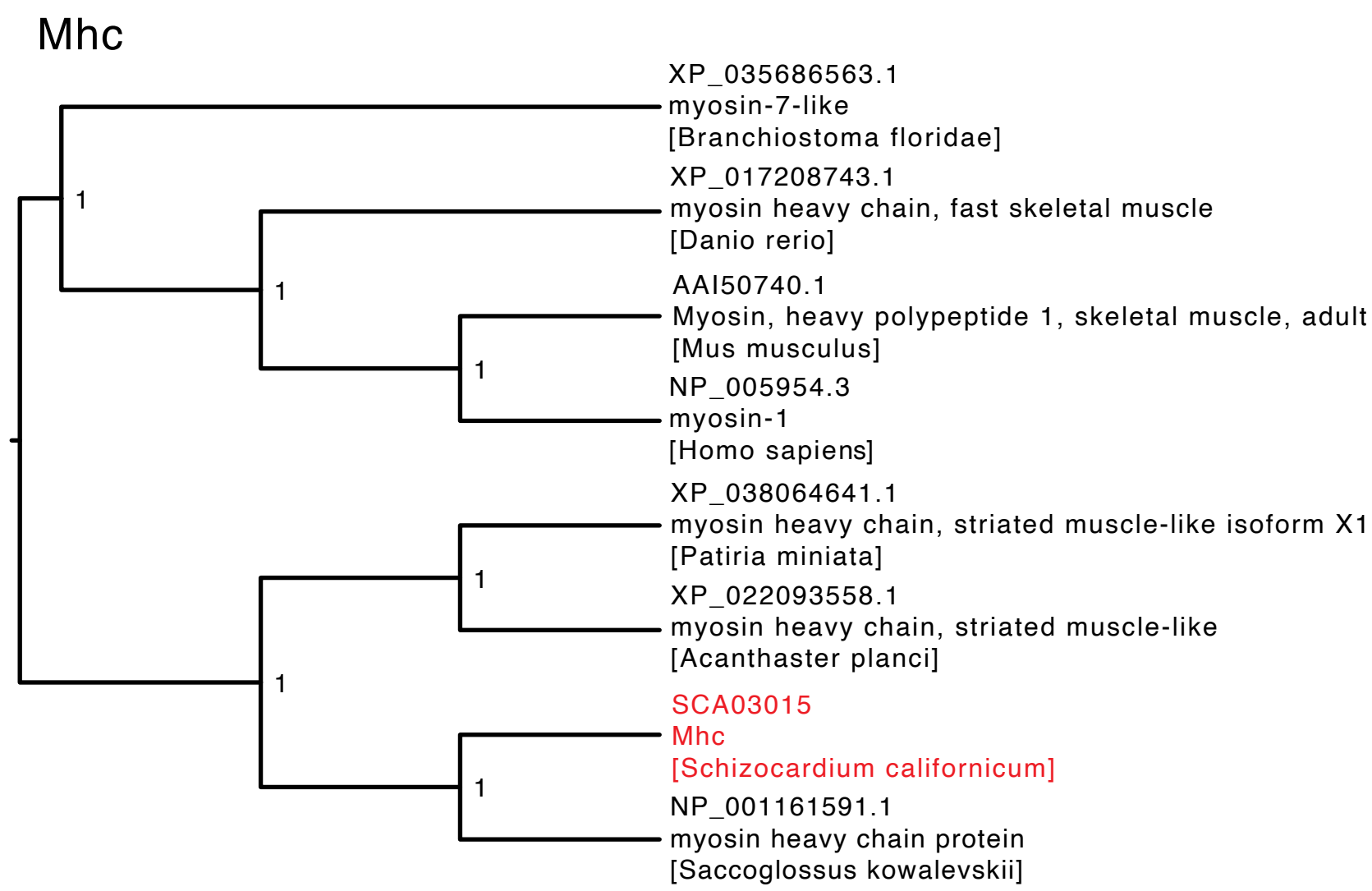

Supplemental Figure 9. Phylogenetic trees of Schizocardium californicum orthologs.

Supplemental Figure 10

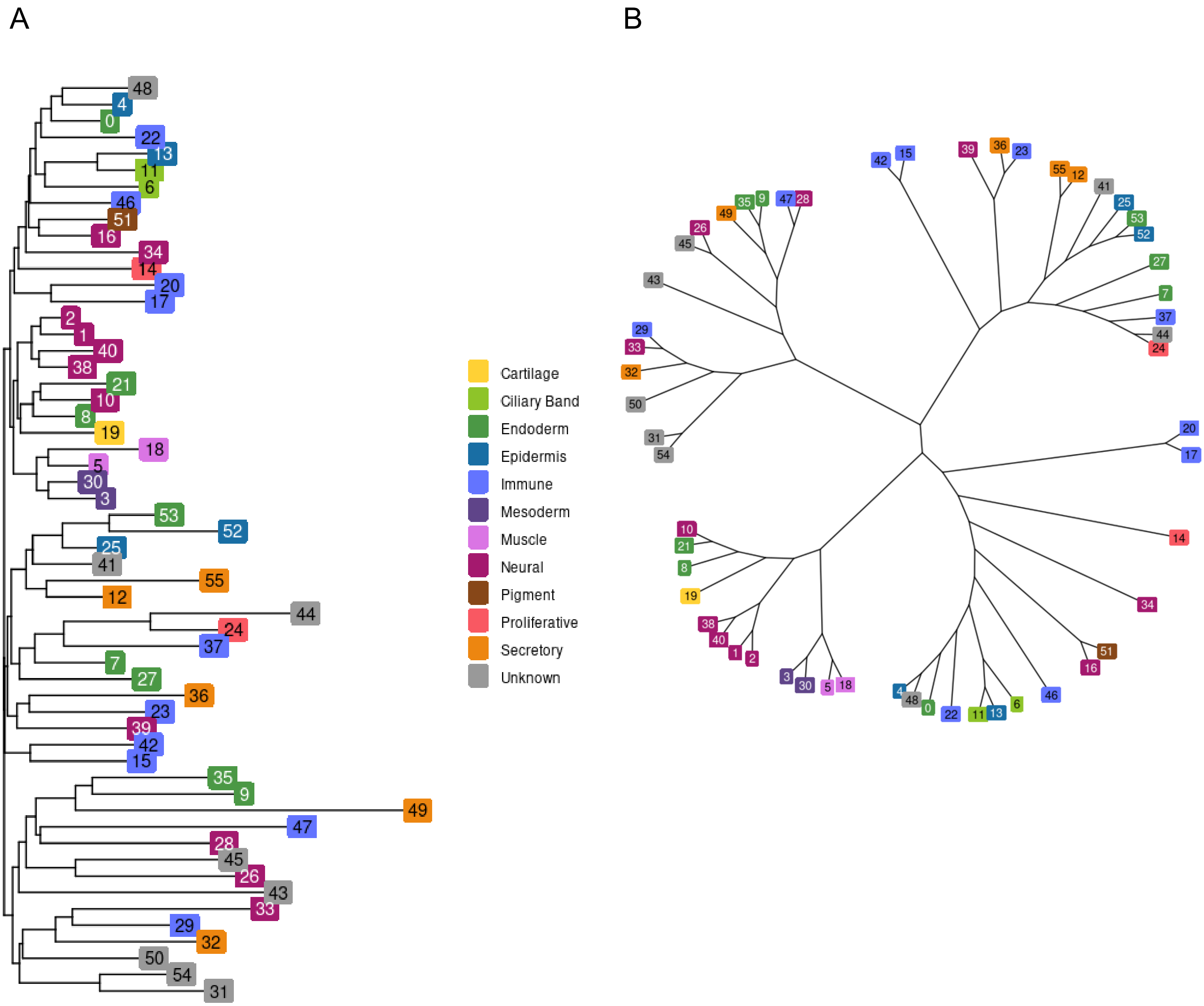

Supplemental Figure 10. Neighbor-joining approach. As described by Musser et al. (2021). A) Standard and B) Daylight embeddings.

Supplemental Tables:

Supplemental Table 1. Cluster Information. scRNA-seq statistics for 56 clusters identified and cell class annotation. Note, clustering was done including an additional sample enriched for juvenile proboscis cells, however these cells were excluded in downstream analyses.

Supplemental Table 2. Cell Class Information. Sheet 1. Cell class markers including gene nomenclature, figure key, function based on literature, citations, and previous markers known in hemichordates highlighted in mustard yellow. Sheet 2. Unknown clusters including putative annotations.

Supplemental Table 3. Cell Cluster Markers. Cell cluster markers obtained by analysis of differential expression (log fold change and p-value) and specificity (how frequently a marker appears).

Supplemental Table 4. HCR Target Sequence. HCR probe target sequences with corresponding amplifier design (B1, B2, or B3).
