# Supplementary material for: Distinct cell states define larval and adult body plans in a hemichordate
Source: Nat Commun. 2026 Sep 8;17:9358. doi: 10.1038/s41467-026-77191-y (PMC13554239; doi:10.1038/s41467-026-77191-y)
Supplement: Supplementary file 2 [file 41467_2026_77191_MOESM2_ESM.pdf]

nature portfolio

Peer Review File

Distinct cell states define larval and adult body plans in a hemichordate

Corresponding Author: Professor Christopher Lowe

This file contains all reviewer reports in order by version, followed by all author rebuttals in order by version.

Version 0:

Reviewer comments:

Reviewer #1

(Remarks to the Author)
Bump et al. employed a comprehensive single-cell RNA sequencing to characterize the cellular composition and transcriptional dynamics throughout metamorphosis in the hemichordate Schizocardium californicum. They proposed that distinct cell states define larval and adult body plans in a hemichordate. The scientific topic is of general interesting but the evidence provided is somewhat far from convincing. There are some key points that remain to be addressed.

Major Comments:
1. The manuscript defined 56 clusters and identified them as 12 cell types (cartilage, ciliary band, endoderm, epidermis, immune, mesoderm, muscle, neural, pigment, proliferative, secretory, and unknown). The annotation of cell types is critical for subsequent analysis. While the authors provided marker genes for each cell cluster in Supplemental Table 2, it remains unclear which key genes were primarily used to define the cell types. In Figure 2B, the authors list only 2-4 marker genes for each cell type, which seems insufficient to robustly define a cell type. For instance, the known function of pif1-7 gene, listed as a marker for the proliferative cells, does not directly relate to cell proliferation. It would enhance the reliability of cell type annotation if the authors provide further analyses by examining the expression specificity of well-known cell type markers (>10 defining marker genes per cell type, preferably with source literature summarized in a suppl. table) from the hemichordates and the classic model animals (e.g., sea urchin) in the cell types defined in this study.

2. Several of the defined cell classes contain multiple clusters. For instance, the endoderm includes eight clusters, the immune system includes ten, and the secretory system contains five. These clusters appear widely dispersed in UMAP space (Supplemental Figures 2A, 4A, 6A, etc.), makeing it difficult to understand why they are considered as the same-origin cell type. Even if they share a broad classification, there should be substantial functional heterogeneity among these clusters, but the authors does not discuss in sufficient detail. A deeper analysis and discussion of these differences are necessary.

3. The clear separation of larval and juvenile cells in the integrated analysis (UMAP) is surprising. In Figure 5, the authors listed some GO terms enriched in differentially expressed genes between larval and juvenile cells to explain the cell type diversity. One term specifically enriched in larvae is "catabolic process". As catabolic processes are fundamental to core metabolism and energy production (often involving housekeeping genes), their enrichment might reflect differing metabolic states rather than cell type states. It would be informative to know if the observed stage-specific clustering pattern remains robust after excluding broadly expressed metabolic/housekeeping genes. Could the strong separation be partly driven by such global physiological differences rather than, or in addition to, cell state differences?

4. Figure 3B already shows differences in cell types between life stages, and pulse-chase experiments revealed that some larval cells persist into the juvenile stage. However, the manuscript does not fully clarify which cells in the juvenile stage are newly produced and which are carried over from the larval stage. Performing developmental trajectory analyses (e.g., pseudotime or lineage inference) across the sampled stages could provide powerful insights into cell fate continuity and transformation. This would also be valuable for understanding the origin and potential fate of cells in the "unknown" clusters.

5. Figure 5E-H shows that both transcription factor (TF) and non-TF genes contribute to the life-stage separation. However, the authors do not discuss which specific genes have the most significant impact on this separation. I strongly recommend that the authors conduct further analysis of a set of genes responsible for distinguishing larval and juvenile transcriptional

states (e.g., the 'larval-territory' and 'adult-territory' genes such as caveolin/innexin and ATP1b as reported by Wang et al., Nat Ecol Evol 2020) with experimental validations by in situ hybridization (ISH). Providing these additional analysis and discussion of relevant implications would greatly improve the analytical depth of this study.

Minor Suggestion:
In the Results section, the authors describe morphological features of the five different developmental stages in detail. This section spans over two pages, which could distract from the main focus of the paper. I suggest shortening this section and move the detailed description into suppl. info.

Reviewer #2

(Remarks to the Author)
In "Distinct cell states define larval and adult body plans in a hemichordate", Bump and co-authors use single-cell transcriptomics to demonstrate that extensive transcriptional reprogramming underpins larval metamorphosis in an indirect developer. Animals display different life strategies, ranging from lineages with direct development to others with radically distinct, independent intermediate life stages (i.e., larvae). How these strategies evolved and how larvae and adults are developmentally connected through metamorphosis remain open questions in biology. Here, the authors address these questions by characterising the cell type diversity in early and late larvae, metamorphosing larvae, and early and late juveniles of the hemichordate S. californicum, a species with maximally indirect development and a head larva (i.e., the juvenile trunk forms during metamorphosis). Profiling ~87,000 cells, the authors identified 56 clusters generally grouped into 12 major cell classes, including ciliary bands, endoderm, epidermis, immune cells, muscle and neurons, among others. They use in situ hybridisation chain reaction (HCR) to experimentally validate selected markers for these clusters. Importantly, because the authors sampled different life stages, they could explore how these cell types change across life stages, discovering that cells clustered by stage rather than by cell type identity. This meant that, e.g., the endoderm of the larvae was transcriptionally more similar to the ciliary band cells present in that stage than to the juvenile endodermal cells. This striking finding is supported by multiple data integration methods (rPCA, Harmony, and Portal) and hierarchical clustering. Interestingly, muscle/mesoderm was the only cell type to exhibit high transcriptional similarity within cell type rather than across life stages, aligning well with the conservation of larval musculature into the juvenile as metamorphosis proceeds. To further investigate the relationships between larval and juvenile cell types, the authors performed pulse-and-chase experiments with EdU to label cells undergoing DNA replication in early and late larvae and trace their fate after metamorphosis, demonstrating that some larval cells persist into the juvenile. Together, the authors conclude that metamorphosis in this indirect-developing hemichordate involves extensive cellular reprogramming, which is significant because the traditional scenario to explain maximally indirect development was through the idea of set-aside cells, or undifferentiated cells that are maintained as stem cells in the larva and are later used to build the juvenile during metamorphosis. Overall, this is a well-executed and important contribution to the field of evo-devo that, by providing high-quality datasets for an indirect developing hemichordate, sets the foundations for multiple future investigations, from the evolution of cell types to the mechanisms of cellular reprogramming.

The analyses are sound, nicely presented, and broadly support the conclusions. I do not have major criticisms, but a few suggestions that might broaden the biological significance of the data presented.

1) Cell clusters of unknown identity. The authors could not assign eight clusters to a specific cell identity. Are these clusters dominated by hemichordate-specific genes with unclear orthology (hence the difficulties in assigning an identity), or is it that they have only a few marker genes? It is interesting that five of these clusters are strongly stage-specific (e.g., cluster 48 is enriched in late larva, cluster 44 is exclusively in metamorphosis, clusters 31, 41 and 54 are enriched or exclusive to juveniles). Have the authors validated some of these clusters? Do they truly represent well-defined, stage-specific cell types?

2) Cluster 44. Following up on the point above, the metamorphosis-specific cluster (cluster 44) is exciting as it is more transcriptionally similar to proliferative cells (cluster 24; Figure 4B). Could the authors elaborate a bit more on this cluster, the markers that define it, etc.? Is there anything particular about it? Could this be "transdifferentiating or reprogramming cells"?

3) To facilitate the reuse of all the valuable data, I encourage the authors to establish an open repository with their code.

Minor points/text edits:

- Line 129: comma after "At the most dorsal anterior,"
- Line 142: comma after "In the mesoderm,"
- Line 157: comma after "At this stage,"
- Line 160: comma after "towards the trunk, and"
- Line 173: comma after "distinct life history stages,"
- Line 182: comma after "Based on cluster-specific gene expression,"
- Line 197-198: better spell out the genus names when there are two similar abbreviations in the same sentence.
- Line 204: "while fzd1/2/7 is expressed" to be consistent with the tense in previous sentences.
- Line 218: comma after "Using HCR,"
- Line 220: comma after "2H), as well as"
- Line 224: "Expression of myl"
- Line 250: comma after "via HCR imaging,"
- Line 263: comma after "(374 for cluster 49),

Reviewer #3

(Remarks to the Author)
The paper entitled Distinct cell states define larval and adult body plans in a hemichordate by Bump and colleagues tackle the question of what are the cell types present during the life cycle of the indirectly developing hemichordate Schizocardium californicum.

The authors use single cell transcriptomics and high-resolution microscopy to map and compare gene expression profiles across a developmental window ranging from early larvae to post-metamorphic juveniles. Using multiple single-cell integration approaches, the authors show that most cell types appear to be specific to either pre-metamorphic or post-metamorphic stages, suggesting divergence of molecular fingerprints between the two life histories. However, they also identify clusters corresponding to mesodermal derivatives, including muscles, that are shared across stages. Overall, the data provided in this study and the resulting conclusions bridge a fundamental gap in our understanding of animal development and evolution, and are of significant importance to the cell-type evolution community, as extensive datasets from hemichordates are currently lacking

Despite the extraordinary quality of the imaging data and the single cell transcriptomic analysis the manuscript has a few weaknesses.

Major points:

We think it would be useful if the authors dug a bit further into the cell types present and their molecular profiles. It would also be helpful if tables of marker genes were provided, allowing the reader to assess the cell type classifications using more than a handful of genes. Is it possible for the authors to refine their cell type groupings for broad classes such as ectoderm, endoderm, immune, and mesoderm? For instance, do they identify ectodermal clusters corresponding to the apical plate? In a similar vein, the authors mention that the two mesoderm clusters seem to correspond to the developing or formed coeloms. Why not name this cell class “coeloms”?

Regarding the secretory clusters, the grouping feels somewhat superficial, as secretory cells can represent a broad spectrum of cell types originating from multiple lineages, not only the epidermis as highlighted in this section. In particular, amy1 expression appears to mark non-epidermal cell types based on its expression pattern. The authors also state that amy1 is associated with the larval stomach. Is it expressed within the larval stomach, similar to other amylases that label acinar-like cells in sea urchins, or is it found in cells surrounding the stomach?

An interesting question is whether clearly separated larval and juvenile clusters can still reflect lineage relationships, especially in stages where lineage is assumed rather than reconstructed, such as the postmetamorphic juvenile. The authors could consider approaches such as building a cell type tree using a neighbor-joining method, as in Musser et al., 2022 (https://pmc.ncbi.nlm.nih.gov/articles/PMC9233960/). This could highlight lineage relationships of cell types in both larval and juvenile stages and serve as an independent validation of the cluster centroid analysis, strengthening the reader’s confidence in and understanding of the results.

The authors state (lines 433–443) that clustering analyses using only transcription factors and only non–transcription factor genes demonstrate some cell separation by life stage. While this appears true, it is also evident that the UMAP structure partially collapses into a cloud-like shape when using transcription factors alone, compared with the well-structured UMAP in the full analysis. This could suggest a higher similarity of regulatory programs and pleiotropic expression of transcription factors across clusters and cell type classes in hemichordates. We think the authors should further comment on and discuss this point. In contrast, the non–transcription factor analysis is able to recover the overall clustering structure, which is expected because it includes the bulk of terminal differentiation genes that define cell types.

The authors mention the presence of serotonergic neurons that expand during larval development in the Results section. However, there is no mention of this neuronal type in the dataset. Are they absent from the data? If so, the authors should highlight this. Otherwise they should include this neuronal population in their analysis, as it is one of the best-studied cell types in hemichordates.
Unless we are mistaken, there are no scripts or links to the scripts related to the analysis provided.

Minor points:

To a non-expert it is hard to understand which cell types and markers were known before. The authors could modify the respective sections in results to make this more clear.

While the introduction is really well-written we find that a section on how fundamental hemichordates are for evolutionary biology is missing. While many of us already know, it would be great to highlight also here for the non-expert reader.

It is not clear what gene-naming strategy the authors are using. For instance, the common abbreviation for dopamine-beta-hydroxylase is dbh and not dopo. We recommend that the authors clearly state the naming strategy used, check all gene names and abbreviations throughout the manuscript, and ensure consistency.

Lines 38-39: “a few select species” should read “a few selected species”

Line 136: Are there sphincters that make the gut tripartite? If it would be good to mention.

Line 361: “These cell population include” should read “These cell populations include”

Line 536: Cell viability was checked with Hoechst and counted on a hemocytometer. It is not clear how Hoechst that labels both dead and alive cells was used to estimate cell viability. Did the authors also use dyes that specifically label alive or dead cells?

Line 570: It is unclear what the reads were mapped to.

Supplemental Figure 2 C: the color code suggests that fapb6 expressing cells correspond to cluster 0, but this is in contrast with what reported in panel B of the same figure. Perhaps the authors meant cluster 35, instead? Also in terms of number of cells this appears more likely.

Reviewer #4

(Remarks to the Author)
I co-reviewed this manuscript with one of the reviewers who provided the listed reports. This is part of the Nature Communications initiative to facilitate training in peer review and to provide appropriate recognition for Early Career Researchers who co-review manuscripts.

Version 1:

Reviewer comments:

Reviewer #1

(Remarks to the Author)
In the revised version of the manuscript, the authors addressed most of the concerns raised by the reviewer. In particular, the revised explanation of the broad cell-class annotations and intra-class heterogeneity has improved the clarity of the manuscript. However, I still have major concerns which I do not think have been fully addressed.

The authors added PAGA connectivity analysis and additional EdU/HCR evidence, but these additions do not fully resolve the original concern. The current PAGA analysis provides a broad graph-based relationship among clusters, but it does not clearly identify cross-stage continuity within specific cell classes or distinguish juvenile populations that may be carried over from larval cells from those that may be newly produced or newly differentiated. I agree that the authors do not need to force a global developmental trajectory across the entire dataset; however, a more stratified analysis may be more informative and biologically appropriate. For example, the authors could consider performing trajectory or cross-stage mapping analyses separately within major cell classes, and/or provide a juvenile–larval cell-state similarity heatmap or continuity score. Moreover, for the unknown clusters, marker annotation alone does not fully address their possible developmental origin or fate. The authors suggest that some unknown clusters may represent transient developmental states or specialized cell populations. This is an interesting possibility, but it would be strengthened by placing these unknown clusters into a more explicit developmental or transcriptional context. For instance, unknown clusters could be included in the class-level trajectory or similarity analyses together with neighboring annotated cell classes. HCR validation of one or two representative unknown-cluster markers, especially for stage-specific or metamorphosis-enriched unknown clusters, would also substantially strengthen this interpretation.

(Remarks on code availability)

Reviewer #2

(Remarks to the Author)
The authors have satisfactorily addressed my previous comments, and the manuscript is now improved. I commend the authors for their beautiful work.

(Remarks on code availability)
The code is sufficient.

Reviewer #3

(Remarks to the Author)

The authors have adequately addressed our previous comments/suggestions.

Minor comment in Fig2 panels R and U there is a white vertical line visible, possibly a result from cropping the images. Please edit accordingly.

(Remarks on code availability)

Open Access This Peer Review File is licensed under a Creative Commons Attribution 4.0 International License, which permits use, sharing, adaptation, distribution and reproduction in any medium or format, as long as you give appropriate credit to the original author(s) and the source, provide a link to the Creative Commons license, and indicate if changes were made.
In cases where reviewers are anonymous, credit should be given to 'Anonymous Referee' and the source.
The images or other third party material in this Peer Review File are included in the article's Creative Commons license, unless indicated otherwise in a credit line to the material. If material is not included in the article's Creative Commons license and your intended use is not permitted by statutory regulation or exceeds the permitted use, you will need to obtain permission directly from the copyright holder.
To view a copy of this license, visit https://creativecommons.org/licenses/by/4.0/

REVIEWER COMMENTS

We thank the Reviewers for their thoughtful and constructive evaluation of our manuscript; *Distinct cell states define larval and adult body plans in a hemichordate*. We appreciate the reviewers' careful assessment and valuable suggestions, which have helped us improve the clarity, rigor, and presentation of our study. In response to the comments, we have clarified our cell-class annotation framework, performed new analyses to evaluate the basis of larval and juvenile transcriptional differences and incorporated an alternative tree-based approach to assess cell-state relationships. We believe these revisions have strengthened the manuscript considerably. Below, we provide a detailed point-by-point response to each reviewer comment, indicating the corresponding changes made in the revised manuscript.

Reviewer #1 (Remarks to the Author):

Bump et al. employed a comprehensive single-cell RNA sequencing to characterize the cellular composition and transcriptional dynamics throughout metamorphosis in the hemichordate Schizocardium californicum. They proposed that distinct cell states define larval and adult body plans in a hemichordate. The scientific topic is of general interesting but the evidence provided is somewhat far from convincing. There are some key points that remain to be addressed.

We appreciate the reviewers thorough reading of our manuscript and that they found the topic interesting. We have provided several of additional analyses as well as reorganized portions of the manuscript based on the reviewer's suggestions.

Major Comments:

1. The manuscript defined 56 clusters and identified them as 12 cell types (cartilage, ciliary band, endoderm, epidermis, immune, mesoderm, muscle, neural, pigment, proliferative, secretory, and unknown). The annotation of cell types is critical for subsequent analysis. While the authors provided marker genes for each cell cluster in Supplemental Table 2, it remains unclear which key genes were primarily used to define the cell types. In Figure 2B, the authors list only 2-4 marker genes for each cell type, which seems insufficient to robustly define a cell type. For instance, the known function of pif1-7 gene, listed as a marker for the proliferative cells, does not directly relate to cell proliferation. It would enhance the reliability of cell type annotation if the authors provide further analyses by examining the expression specificity of well-known cell type markers (>10 defining marker genes per cell type, preferably with source literature summarized in a suppl. table) from the hemichordates and the classic model animals (e.g., sea urchin) in the cell types defined in this study.

Response:
We agree that robust cell-class annotation is essential for the downstream analyses. In the revised manuscript, we have expanded the annotation framework substantially. In Supplemental Table 2 we now provide at least ten defining marker genes for each cell class, including associated functional and literature support and highlight genes which have previously been studied in hemichordates. This table is now referred to earlier in the Results so that readers encounter the annotation criteria before the downstream analyses. Furthermore, Supplemental Figures 2–7 now show broader marker sets for each major class, while Figure 2B has been relabeled as 'Selected Marker Genes, Grouped by Cell Class' to make clear that it is a representative summary rather than the full basis for annotation.

We also clarified the proliferative-cell annotation. In addition to *pif1*, this class is supported by genes previously associated with irradiation-sensitive proliferative cells in *S. californicum* (Bump et al 2022), including *lbr-1* and *dlgp5*, as well as HCR validation for *pif1* in Supplemental Figure 7C. We now discuss this explicitly in the text.

We have changed the text:

Original text:
We generated an atlas of S. californicum across life history stages by clustering cellular profiles, identifying 56 clusters containing between 68 and 8,392 cells (average 1,554 cells/cluster) (Figure 2A, Supplemental Figure 1A, Supplemental Table 1). Cell clusters are defined by markers obtained by analysis of differential expression (log fold change and p-value) and specificity (Supplemental Table 2). Based on cluster-specific gene expression we further organized these cell type clusters into 12 cell classes (cartilage, ciliary band, endoderm, epidermis, immune cells, mesoderm, muscle, neural, pigment, proliferative, secretory, and unknown) (Figure 2B, Supplemental Table 2).

We validated our cell type clusters using both newly discovered markers from our cluster annotations (Supplemental Table 2, 3) and known tissue markers in hemichordates, by plotting the relative expression of a gene of interest in each cell class (Figure 2B) and then performing HCR (hybridization chain reaction) fluorescent in situ hybridization (Figures 2C-T, Supplemental Table 4).

New text:
We generated an atlas of *S. californicum* across life history stages by clustering cellular profiles, identifying 56 clusters containing between 68 and 8,392 cells (average 1,554 cells/cluster) (Figure 2A, Supplemental Figure 1A, Supplemental Table 1). Using the top cluster-specific genes, known hemichordate markers, and functional annotation, we

organized these cell type clusters into 12 cell classes (cartilage, ciliary band, endoderm, epidermis, immune cells, mesoderm, muscle, neural, pigment, proliferative, secretory, and unknown) (selected markers in Figure 2B, full markers in Supplemental Table 2 and Supplemental Figures 2-8). Cell clusters are defined by markers obtained by analysis of differential expression (log fold change and p-value) and specificity (Supplemental Table 3).

We validated our cell type clusters using known marker genes from other species, newly discovered markers from our cluster annotations, and known tissue markers in hemichordates, by plotting the relative expression of a selected markers in each cell class (Figure 2B) and then performing HCR (hybridization chain reaction) fluorescent *in situ* hybridization (Figures 2C-V, Supplemental Table 4).

2. Several of the defined cell classes contain multiple clusters. For instance, the endoderm includes eight clusters, the immune system includes ten, and the secretory system contains five. These clusters appear widely dispersed in UMAP space (Supplemental Figures 2A, 4A, 6A, etc.), making it difficult to understand why they are considered as the same-origin cell type. Even if they share a broad classification, there should be substantial functional heterogeneity among these clusters, but the authors does not discuss in sufficient detail. A deeper analysis and discussion of these differences are necessary.

Response:
We operationally define cell classes based on shared molecular and functional properties rather than proximity in the global UMAP embedding or inferred lineage relationships. Since the global embedding reflects transcriptional variation across all expressed genes, including strong life-stage-associated physiological programs, functionally related cell types do not necessarily occupy adjacent positions in two-dimensional UMAP space.

We agree that broad classes such as neural, immune, or secretory encompass substantial internal heterogeneity. To better clarify this structure, we expanded the marker analyses in Supplemental Table 3 and Supplemental Figures 2–7 to distinguish both (1) markers shared across a class and (2) markers distinguishing individual clusters within a class.

For example, the neural class includes clusters 1, 2, 10, 16, 26, 28, 33, 34, 38, 39, and 40, which are distributed broadly across the global embedding. Despite this dispersion, these clusters share neural-associated markers and functional signatures, including *vglu, syn2,* and *rhg39*, while also exhibiting cluster-specific differences. For example,

cluster 16 is enriched for *alx1* and *tbrain*, whereas cluster 1 expresses *ox2r* and *syt11*. Subclustering of neural-class cells further revealed substantial organization by life-history stage, highlighting the existence of stage-specific neuronal states within the broader neural class (See data below, new Supplemental Figure 5H).

H

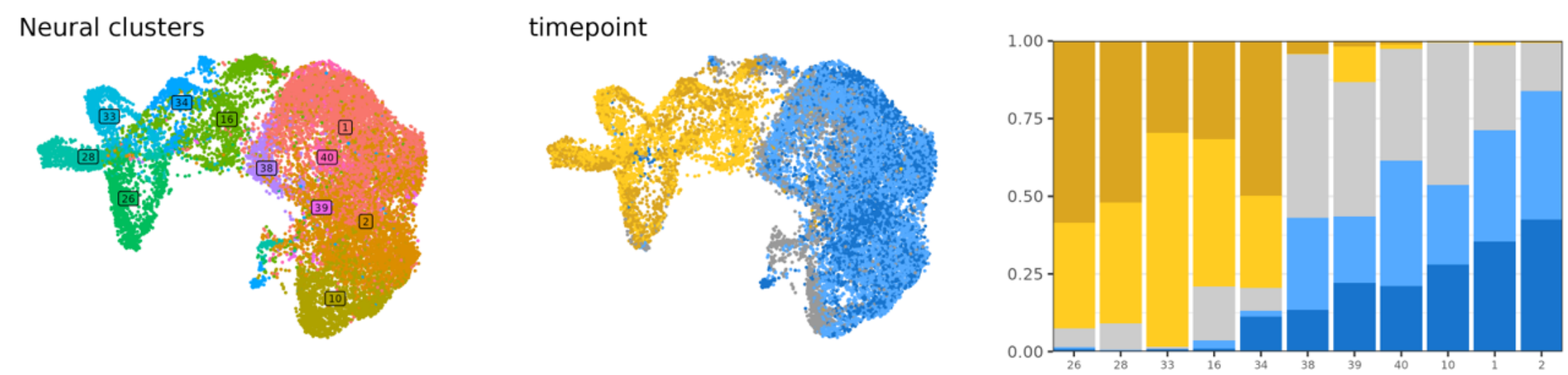

Original Text Results: Based on our annotation, neural cells are one of most broadly distributed classes of cell types in both larvae and juveniles. Some cell types were present in both life stages but varied in their life stage composition, with a key example being the neural cell types (clusters 26, 16, 34, 1, 2 dashed boxes in Figure 3B with corresponding panels in Figure 3C-E).

New text: Based on our annotation, neural cells represent one of the most broadly distributed cell classes across both larval and juvenile life-history stages. The neural cell class encompasses clusters 1, 2, 10, 16, 26, 28, 33, 34, 38, 39, and 40, which are widely dispersed across the global UMAP embedding (Supplemental Figure 5A). Despite this dispersion, examination of cluster-specific markers (Supplemental Table 3) revealed shared neural-associated molecular signatures that allowed these clusters to be grouped together into a broader neural cell class (Figure 2B; Supplemental Figure 5B).

Cells from the neural class recovered substantial organization by life-history stage, indicating that broadly conserved neural identity is accompanied by stage-specific transcriptional states (Supplemental Figure 5H). Within this broader neural class, individual clusters also exhibit distinct molecular profiles. For example, cluster 16 is enriched for genes previously associated with hemichordate neural patterning such as *t-brain*, whereas cluster 1 is enriched for neural genes in other phyla that have not been previously characterized in hemichordate neurobiology, such as *ox2r* (orexin receptor type 2) and *syt11* (synaptotagmin-11). We also identified genes broadly shared across the neural class, including the known neural marker *rhg39* (Rho GTPase-activating protein 39).

To examine these life-history-related neural differences in greater detail, we next focused on neural clusters 26, 16, 34, 1, and 2 (dashed boxes in Figure 3B, corresponding to panels in Figure 3C–E).

3. The clear separation of larval and juvenile cells in the integrated analysis (UMAP) is surprising. In Figure 5, the authors listed some GO terms enriched in differentially expressed genes between larval and juvenile cells to explain the cell type diversity. One term specifically enriched in larvae is "catabolic process". As catabolic processes are fundamental to core metabolism and energy production (often involving housekeeping genes), their enrichment might reflect differing metabolic states rather than cell type states. It would be informative to know if the observed stage-specific clustering pattern remains robust after excluding broadly expressed metabolic/housekeeping genes. Could the strong separation be partly driven by such global physiological differences rather than, or in addition to, cell state differences?

Response:
We agree that the strong separation between larval and juvenile cell states could potentially reflect broad physiological or metabolic differences rather than differences in cell state alone. To address this possibility, we performed additional analyses examining the contribution of different functional gene categories to the clustering structure.

Specifically, we repeated the PCA and UMAP analyses using only transcription factors, only metabolic genes, only catabolic genes, or all remaining genes (new Figure 6). We additionally repeated these analyses after removing the top 5% of differentially expressed genes from each category. In all cases, separation between larval and juvenile states remained broadly preserved. Importantly, removal of strongly differentially expressed metabolic and catabolic genes did not collapse the life-stage separation.

Together, these analyses indicate that the transcriptional distinction between larval and juvenile states cannot be explained solely by broad metabolic or housekeeping differences, but instead reflects distributed differences across regulatory, physiological, and cell-state-associated programs. In response to these questions and the questions raised in comment 5 we have edited the text to reflect these new analyses and provided updated text in response to comment 5.

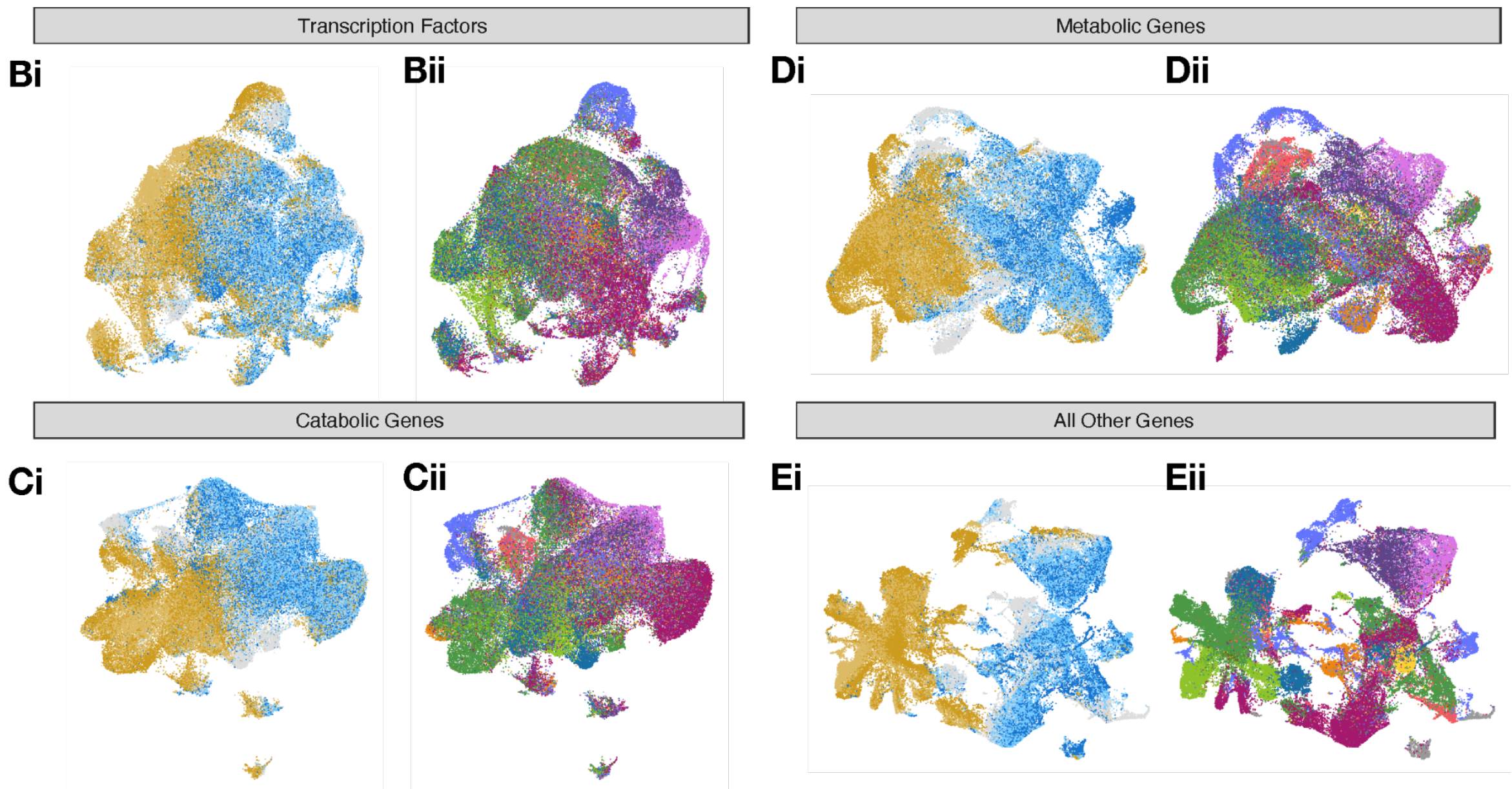

4. Figure 3B already shows differences in cell types between life stages, and pulse-chase experiments revealed that some larval cells persist into the juvenile stage. However, the manuscript does not fully clarify which cells in the juvenile stage are newly produced and which are carried over from the larval stage. Performing developmental trajectory analyses (e.g., pseudotime or lineage inference) across the sampled stages could provide powerful insights into cell fate continuity and transformation. This would also be valuable for understanding the origin and potential fate of cells in the "unknown" clusters.

Response:

We agree that trajectory-based approaches could potentially provide important insight into cell fate continuity and transformation across metamorphosis. To explore this, we implemented PAGA connectivity analysis (Wolf et al. 2019), which recovered relationships between clusters broadly consistent with our centroid-based analyses (Figure 4). However, we found that conventional pseudotime approaches were difficult to interpret in this system because larval and juvenile transcriptional states form largely discrete manifolds across widely separated developmental stages and were not included in the manuscript.

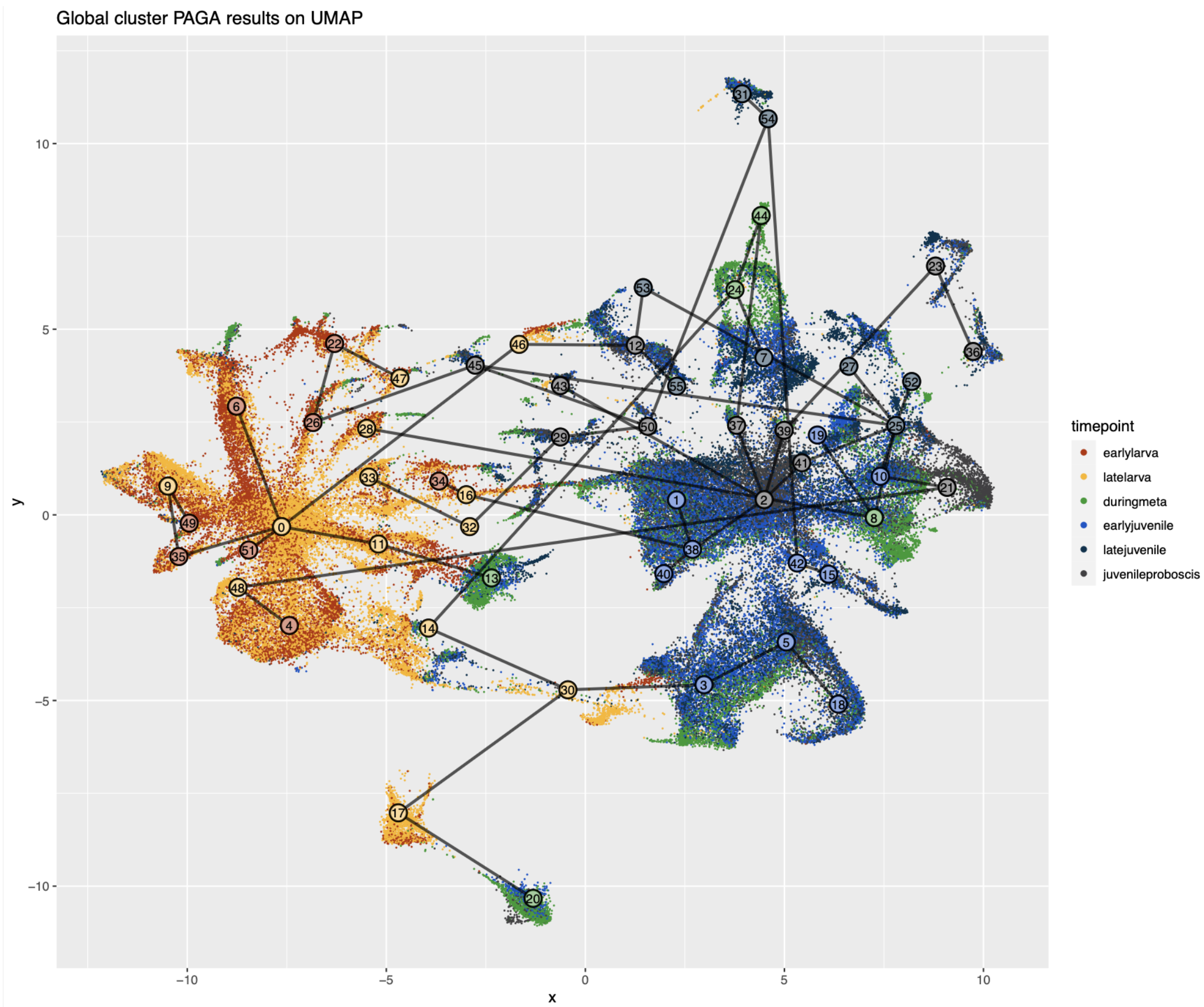

Importantly, our EdU pulse-chase experiments provide independent experimental evidence that at least some larval cells persist into juvenile structures despite substantial transcriptional remodeling during metamorphosis (Figure 3). In addition, HCR labeling of Cluster 1 neural markers combined with EdU pulse-chase labeling revealed co-labeled cells in juveniles, consistent with the persistence of larval cells into juvenile neural populations (See data below, new Supplemental Figure S5I-J). However, because the EdU labeling approach marks broad populations of proliferative larval cells rather than lineage-restricted cell types, the precise relationship between persistent larval cells and juvenile transcriptional identities remains unresolved.

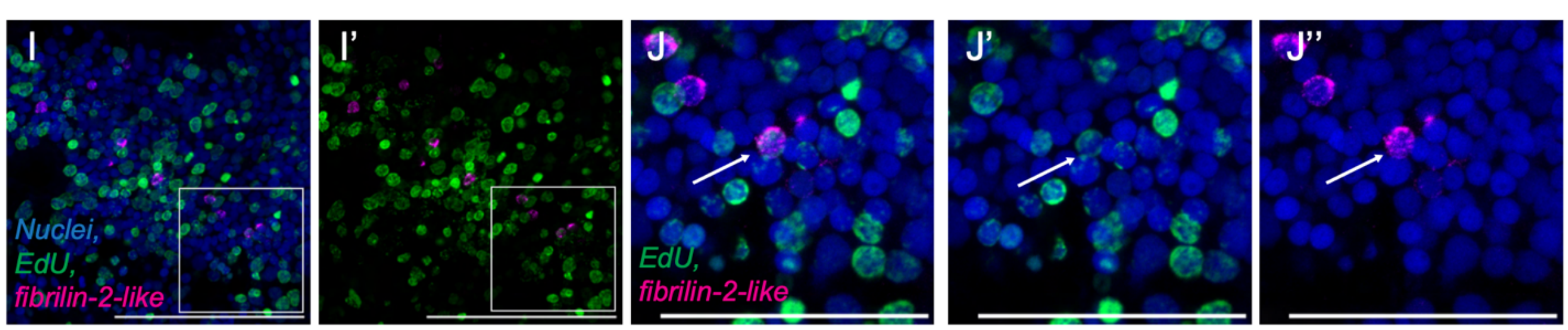

We additionally expanded the annotation of previously ‘unknown’ clusters by providing additional marker information and putative annotations in Supplemental Table 2.

Several of these clusters exhibit strong life-stage enrichment, raising the possibility that they represent transient metamorphic or stage-specific cell states. Resolving the lineage relationships and developmental trajectories of these populations will likely require lineage-specific labeling approaches combined with denser temporal sampling and additional single-cell profiling.

5. Figure 5E-H shows that both transcription factor (TF) and non-TF genes contribute to the life-stage separation. However, the authors do not discuss which specific genes have the most significant impact on this separation. I strongly recommend that the authors conduct further analysis of a set of genes responsible for distinguishing larval and juvenile transcriptional states (e.g., the 'larval-territory' and 'adult-territory' genes such as caveolin/innexin and ATP1b as reported by Wang et al., Nat Ecol Evol 2020) with experimental validations by in situ hybridization (ISH). Providing these additional analysis and discussion of relevant implications would greatly improve the analytical depth of this study.

Response:
We agree that identifying the transcriptional programs underlying larval and juvenile separation is important. To address this, we performed additional differential expression analyses and examined whether a small number of highly differentially expressed genes disproportionately drove the separation between life-history stages.

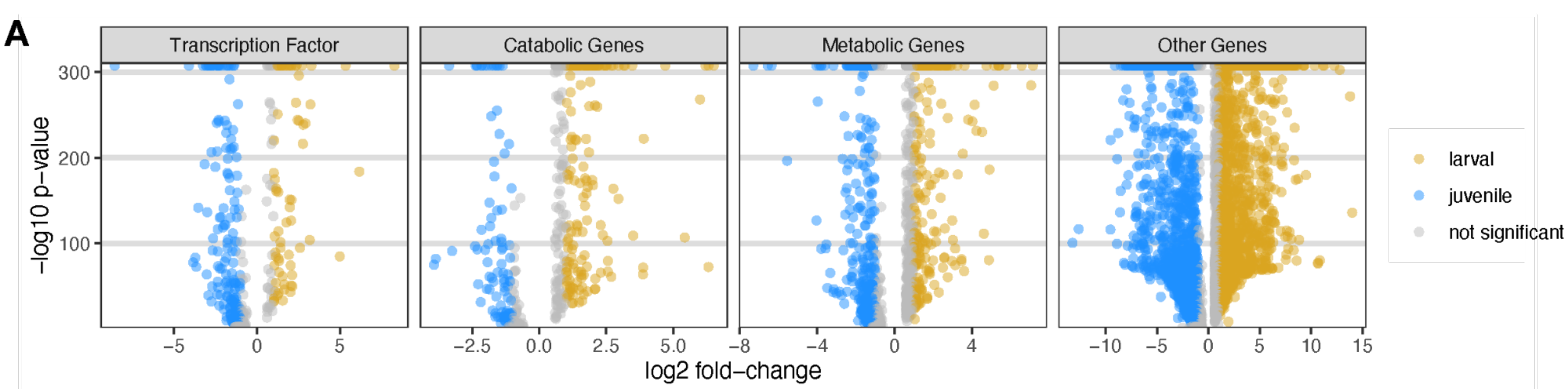

As noted above in response to comment 3 we categorized genes into transcription factor, metabolic, catabolic, and all other gene classes, and repeated PCA and UMAP analyses both before and after removing the top 5% of differentially expressed genes within each category (Figure 6B–E). In all cases, the broad separation between larval and juvenile cell states remained largely preserved. These results indicate that the life-stage separation is not driven by a small number of dominant marker genes but instead reflects distributed transcriptional differences across multiple regulatory and physiological programs.

The distributed nature of these transcriptional differences is also consistent with previous work in indirect developers suggesting that larval and adult body plans are associated with broad territory-level regulatory programs rather than a small number of stage-specific master genes (e.g. Wang et al. 2020). Rather than identifying a single dominant transcriptional module underlying life-history identity, our analyses suggest that larval and juvenile states in S. californicum are defined by broad shifts across multiple regulatory and physiological programs. We now discuss this point explicitly in the revised manuscript and provide the additional analyses in Figure 6. We also checked the mapping of the genes identified in Wang et al. 2020 as having life history association - ATP1b and Calveolin/innexin - and do not find the same relationship.

Original text:
While these patterning genes may reflect the distribution of these cell types across life stages, we next wanted to examine how different types of genes might explain the contrasts between larval and juvenile cell types. To explore this, we ran a principal component (PC) analysis and UMAP embedding on our scRNA-seq data using either exclusively predicted transcription factors (Figure 5E, 5F) or non-transcription factor genes (Figure 5G, 5H). Both analyses demonstrate some cell separation by life stage (Figure 5E, 5G) and recapitulate separation of the broad cell types (Figure 5F, 5H) we originally identified (Figure 2A). This suggests that both transcription factor and non-transcription factor genes reflect the large effect of physiology and homeostasis between life history stages. This approach demonstrates that regulatory, physiological, and homeostatic programs are all involved in determining cell type composition and transcriptional signatures across disparate life stages. These differences are perhaps linked to the differing ecological demands that body plan morphology must meet as a planktonic larva versus as a mud-dwelling benthic juvenile.

New text (added also in response to comment 3):

While these patterning genes may reflect the distribution of cell types across life stages, we next asked whether the transcriptional differences between larval and juvenile cell types were disproportionately driven by a small number of highly differentially expressed genes from specific functional classes (Fig. 6A). To address this, we performed principal component (PC) analysis and UMAP embedding on the scRNA-seq dataset using restricted subsets of genes. When analyses were performed using only predicted transcription factors (Fig. 6Bi–ii), cells separated strongly by life-history stage while still recapitulating the major cell-class relationships identified in the full dataset (Fig. 2A). Removal of the top 5% most differentially expressed transcription factors had little effect on either life-stage separation or overall cell-class organization (Fig. 6Biii–iv).

We next repeated this analysis using catabolic genes (Fig. 6Ci–iv), metabolic genes (Fig. 6Di–iv), and all remaining genes outside these categories (Fig. 6Ei–iv). In each case, cells continued to separate primarily by life-history stage while maintaining the broad cell-class structure observed in the full atlas. Importantly, removal of the top 5% most differentially expressed genes within each category again produced only minor changes to the overall organization of the UMAPs. Although the transcription factor-only embedding exhibited less sharply resolved structure than analyses using the full transcriptome or non-transcription factor genes, we interpret this cautiously because dimensionality reduction can be sensitive to feature number and variance structure. Nevertheless, the broader organization of the transcription factor-only UMAP may reflect the pleiotropic deployment of regulatory factors across multiple cell classes relative to the more cell-type-restricted expression of terminal differentiation genes.

We next repeated this analysis using catabolic genes (Figure 6Ci–iv), metabolic genes (Figure 6Di–iv), and all remaining genes outside these categories (Figure 6Ei–iv). In each case, cells continued to separate primarily by life-history stage while maintaining the broad cell-class structure observed in the full atlas. Importantly, removal of the top 5% most differentially expressed genes within each category again produced only minor changes to the overall organization of the UMAPs.

Together, these analyses indicate that the transcriptional distinction between larval and juvenile states is not driven by a small number of dominant marker genes or restricted to a single functional class of genes. Instead, the divergence reflects broad, distributed differences across multiple regulatory, metabolic, catabolic, and physiological programs. These findings suggest that life-history stage acts as a major organizing axis of transcriptional identity across conserved functional cell classes, likely reflecting the distinct ecological and physiological demands of a planktonic larva versus a benthic juvenile.

Response, part 2: We added an additional paragraph to the discussion to address this point:

Interestingly, the transcriptional differences between larval and juvenile cell states are not driven by a small number of stage-specific genes or restricted to a single functional category. Instead, the divergence is broadly distributed across regulatory, metabolic, catabolic, and other physiological gene programs (Figure 6). We do not observe a pattern in which life-history-specific clustering is dominated by a small subset of highly differentially expressed transcripts. Consistent with this, removal of the top 5% of differentially expressed genes within each functional category had little effect on the overall UMAP structure (Figure 6Biii–Eiv). This pattern is congruent with previous work in indirect developers proposing that larval and adult body plans are associated with

broad territory-level transcriptional states rather than discrete stage-specific master regulators (Wang et al., 2020). These results suggest that life-history stage acts as a major organizing axis of transcriptional identity, reshaping broadly conserved functional cell classes across metamorphosis.

Minor Suggestion:
In the Results section, the authors describe morphological features of the five different developmental stages in detail. This section spans over two pages, which could distract from the main focus of the paper. I suggest shortening this section and move the detailed description into suppl. Info.

At the suggestion of the reviewer, we shortened this section and referred readers to the main reference describing the stage morphology.

Revised section in the results:

Early larva. The first larval stage (22 days post-fertilization; Fig. 1B,G) exhibits a fully established larval body plan without adult structures. The tornaria is a small, planktonic feeder propelled by two ciliary bands: an anterior circumoral loop for feeding and a posterior telotroch for locomotion (Garstang, 1939; Lacalli & Gilmour, 2002; R. Strathmann & Bonar, 1976). The remaining ectoderm is largely squamous, with a ventral neurotroch and an anterior apical organ bearing a ciliary tuft, neurons, and paired eyespots (Gonzalez et al., 2018). Mesoderm is minimal, consisting of a single anterior protocoel and limited musculature associated with the pharynx and apical strand (R. Strathmann & Bonar, 1976; Tagawa et al., 1998). The endoderm forms a tripartite gut (pharynx, stomach, intestine) terminating in an anus.

Late larva. By two months (Fig. 1C,H), larvae are larger, feeding more extensively, and competent for metamorphosis (Hadfield et al., 2001). While retaining overall morphology, this stage shows early adult patterning, including formation of mesocoels and metacoels and the appearance of nascent gill slits. The posterior expands into an anal field, and the nervous system becomes more elaborate, with widespread serotonergic neurons and initial development of the dorsal cord (Gonzalez et al., 2018).

At **mid-metamorphosis** (Fig. 1D,I), larval and adult features coexist. Ectodermal thickening and coelomic expansion accompany trunk initiation as the blastocoel is reduced (Agassiz, 1873; Nielsen & Hay-Schmidt, 2007). The degenerating circumoral band contributes to the proboscis ectoderm, while the anterior coelom expands to form proboscis musculature. The stomochord forms at the proboscis base and supports the heart/kidney complex (Balser & Ruppert, 1990). Nascent gill slits shift posteriorly as

trunk elongation begins, and collagenous skeletal elements initiate. Animals remain planktonic and swim using the telotroch.

Juveniles. Early (Fig. 1E,J) and late (Fig. 1F,K) juveniles represent the definitive adult body plan. These benthic worms exhibit the tripartite organization of proboscis, collar, and trunk, with a ventral mouth, anterior trunk gill pores, and both dorsal and ventral nerve cords. Early juveniles have a developed proboscis and collar but limited trunk elongation, whereas late juveniles show substantial trunk extension, capturing the full cellular complement of the adult form.

Reviewer #2 (Remarks to the Author):

In "Distinct cell states define larval and adult body plans in a hemichordate", Bump and co-authors use single-cell transcriptomics to demonstrate that extensive transcriptional reprogramming underpins larval metamorphosis in an indirect developer. Animals display different life strategies, ranging from lineages with direct development to others with radically distinct, independent intermediate life stages (i.e., larvae). How these strategies evolved and how larvae and adults are developmentally connected through metamorphosis remain open questions in biology. Here, the authors address these questions by characterising the cell type diversity in early and late larvae, metamorphosing larvae, and early and late juveniles of the hemichordate S. californicum, a species with maximally indirect development and a head larva (i.e., the juvenile trunk forms during metamorphosis). Profiling ~87,000 cells, the authors identified 56 clusters generally grouped into 12 major cell classes, including ciliary bands, endoderm, epidermis, immune cells, muscle and neurons, among others. They use in situ hybridisation chain reaction (HCR) to experimentally validate selected markers for these clusters. Importantly, because the authors sampled different life stages, they could explore how these cell types change across life stages, discovering that cells clustered by stage rather than by cell type identity. This meant that, e.g., the endoderm of the larvae was transcriptionally more similar to the ciliary band cells present in that stage than to the juvenile endodermal cells. This striking finding is supported by multiple data integration methods (rPCA, Harmony, and Portal) and hierarchical clustering. Interestingly, muscle/mesoderm was the only cell type to exhibit high transcriptional similarity within cell type rather than across life stages, aligning well with the conservation of larval musculature into the juvenile as metamorphosis proceeds. To further investigate the relationships between larval and juvenile cell types, the authors performed pulse-and-chase experiments with EdU to label cells undergoing DNA replication in early and late larvae and trace their fate after metamorphosis, demonstrating that some larval cells persist into the juvenile. Together, the authors conclude that metamorphosis in this indirect-developing hemichordate involves

extensive cellular reprogramming, which is significant because the traditional scenario to explain maximally indirect development was through the idea of set-aside cells, or undifferentiated cells that are maintained as stem cells in the larva and are later used to build the juvenile during metamorphosis. Overall, this is a well-executed and important contribution to the field of evo-devo that, by providing high-quality datasets for an indirect developing hemichordate, sets the foundations for multiple future investigations, from the evolution of cell types to the mechanisms of cellular reprogramming.

The analyses are sound, nicely presented, and broadly support the conclusions. I do not have major criticisms, but a few suggestions that might broaden the biological significance of the data presented.

1) Cell clusters of unknown identity. The authors could not assign eight clusters to a specific cell identity. Are these clusters dominated by hemichordate-specific genes with unclear orthology (hence the difficulties in assigning an identity), or is it that they have only a few marker genes? It is interesting that five of these clusters are strongly stage-specific (e.g., cluster 48 is enriched in late larva, cluster 44 is exclusively in metamorphosis, clusters 31, 41 and 54 are enriched or exclusive to juveniles). Have the authors validated some of these clusters? Do they truly represent well-defined, stage-specific cell types?

Response:
We agree that the cell clusters of unknown identity are very interesting. These clusters are not lacking marker genes but instead are enriched for hemichordate-specific genes or markers that do not clearly resolve a single cell identity. For example, among the top ten marker genes for cluster 43, only one could be assigned a clear orthology relationship to known genes in other phyla. To provide additional context, we have added putative annotations for these unknown clusters in a new tab in Supplemental Table 2.

We have also added a breakdown of life-stage enrichment for these clusters in Supplemental Figure 8. This analysis highlights that cluster 48 (putative metabolic support cells) is enriched in late larvae, cluster 44 (putative active metabolic cells) is enriched at metamorphosis, and clusters 31, 41, 54 are enriched in juveniles and correspond to putative neuroectoderm, proliferative cells, and unidentified states respectively. Several additional clusters, including 45, 43, and 50 may represent transient metamorphic cell states. Higher temporal-resolution sampling through metamorphosis will be required to resolve the identity and developmental trajectories of these populations. We have added an acknowledgement of this interesting observation in the discussion.

2) Cluster 44. Following up on the point above, the metamorphosis-specific cluster (cluster 44) is exciting as it is more transcriptionally similar to proliferative cells (cluster 24; Figure 4B). Could the authors elaborate a bit more on this cluster, the markers that define it, etc.? Is there anything particular about it? Could this be "transdifferentiating or reprogramming cells"?

Response:
Cluster 44 is particularly interesting because it is enriched specifically during metamorphosis and shows transcriptional similarity to proliferative cells (cluster 24). However, the cluster contains a mixed marker profile that makes assignment to a single canonical cell identity difficult. Highly enriched genes include Histone H1-like, associated with chromatin organization and cell-cycle activity; Death-associated protein 1, linked to programmed cell death; Sacsin, involved in cytoskeletal organization; and L-xylulose reductase, associated with carbohydrate metabolism. Based on this combination of markers, we annotated cluster 44 as 'Unknown: Putative Active Metabolic Cells.

3) To facilitate the reuse of all the valuable data, I encourage the authors to establish an open repository with their code.

Response:
We have provided an open github repository of our code [https://github.com/0x644BE25/schizocardium_single-cell] which will have all the analysis and plotting code by publication and hope it is valuable for the community.

Minor points/text edits:

- Line 129: comma after "At the most dorsal anterior,"
- Line 142: comma after "In the mesoderm,"
- Line 157: comma after "At this stage,"
- Line 160: comma after "towards the trunk, and"
- Line 173: comma after "distinct life history stages,"
- Line 182: comma after "Based on cluster-specific gene expression,"
- Line 197-198: better spell out the genus names when there are two similar abbreviations in the same sentence.
- Line 204: "while fzd1/2/7 is expressed" to be consistent with the tense in previous sentences.
- Line 218: comma after "Using HCR,"
- Line 220: comma after "2H), as well as"

- Line 224: "Expression of myl"
- Line 250: comma after "via HCR imaging,"
- Line 263: comma after "(374 for cluster 49),

We appreciate the reviewers' close read of our manuscript and have made those changes.

Reviewer #3 (Remarks to the Author):

The paper entitled Distinct cell states define larval and adult body plans in a hemichordate by Bump and colleagues tackle the question of what are the cell types present during the life cycle of the indirectly developing hemichordate Schizocardium californicum.

The authors use single cell transcriptomics and high-resolution microscopy to map and compare gene expression profiles across a developmental window ranging from early larvae to post-metamorphic juveniles. Using multiple single-cell integration approaches, the authors show that most cell types appear to be specific to either pre-metamorphic or post-metamorphic stages, suggesting divergence of molecular fingerprints between the two life histories. However, they also identify clusters corresponding to mesodermal derivatives, including muscles, that are shared across stages. Overall, the data provided in this study and the resulting conclusions bridge a fundamental gap in our understanding of animal development and evolution, and are of significant importance to the cell-type evolution community, as extensive datasets from hemichordates are currently lacking

Despite the extraordinary quality of the imaging data and the single cell transcriptomic analysis the manuscript has a few weaknesses.

Major points:

We think it would be useful if the authors dug a bit further into the cell types present and their molecular profiles. It would also be helpful if tables of marker genes were provided, allowing the reader to assess the cell type classifications using more than a handful of genes. Is it possible for the authors to refine their cell type groupings for broad classes such as ectoderm, endoderm, immune, and mesoderm? For instance, do they identify ectodermal clusters corresponding to the apical plate? In a similar vein, the authors mention that the two mesoderm clusters seem to correspond to the developing or formed coeloms. Why not name this cell class “coeloms”?

Response:
We appreciate the reviewer's suggestion and note that this was a consistent point raised across reviewers. While a complete, unbiased list of cluster marker genes was already provided in Supplemental Table 2, we have substantially expanded these annotations in the revised manuscript. We have reordered Supplemental Tables 2 and 3 and now provide at least 10 defining marker genes for each cell class, together with relevant summaries and references supporting the annotations.

We also agree that the original presentation of Figure 2B did not sufficiently clarify the basis for cell-class assignment. We have therefore relabeled the axis as 'Selected Marker Genes, Grouped by Cell Class' and now explicitly indicate that comprehensive marker lists are provided in Supplemental Table 2 and Supplemental Figures 2–7.

The reviewer's suggestion to further subdivide broad classes such as ectoderm, endoderm, immune, and mesoderm is well taken. Some classes, such as endoderm, exhibit relatively clear transcriptional signatures, whereas canonical ectodermal markers are less well resolved. Instead, we identify several epidermal populations that are likely predominantly ectodermal, though not necessarily exclusively so. Future work will be needed to resolve transcriptional differences between epidermal populations derived from distinct germ layers.

We do not recover ectodermal clusters corresponding to discrete regional structures such as the apical plate. However, we do identify neuronal populations associated with the apical organ, including cluster 16 marked by pburs expression.

Finally, although two clusters likely correspond to developing coelomic structures in early larvae, we retained the broader annotation of 'mesoderm' because coeloms rapidly transform into differentiated mesodermal derivatives in other later stages.

Regarding the secretory clusters, the grouping feels somewhat superficial, as secretory cells can represent a broad spectrum of cell types originating from multiple lineages, not only the epidermis as highlighted in this section. In particular, amy1 expression appears to mark non-epidermal cell types based on its expression pattern. The authors also state that amy1 is associated with the larval stomach. Is it expressed within the larval stomach, similar to other amylases that label acinar-like cells in sea urchins, or is it found in cells surrounding the stomach?

Response:
We grouped these cells as 'secretory' was based on shared predicted functional properties inferred from their transcriptional profiles rather than lineage relationships.

We agree with the reviewer that secretory cells likely arise from multiple lineages and have revised the text to clarify that this represents a functional rather than developmental classification. Supplemental Figure 6 now provides expanded marker heatmaps and cluster visualizations for these populations.

We also agree that amy1-expressing cells do not appear to correspond to epidermal populations. Based on the HCR data, our interpretation is that these cells occupy the blastocoelar space surrounding the larval stomach rather than the stomach epithelium itself. We have now included additional HCR imaging of amy1 expression (Supplement 6C, also see below). Although these cells also express carboxypeptidase, they do not express high levels of *hnf1a* or *ptf1a*. Future comparative analyses may clarify whether these cells are homologous to the acinar-like digestive cell populations described in echinoderm larvae.

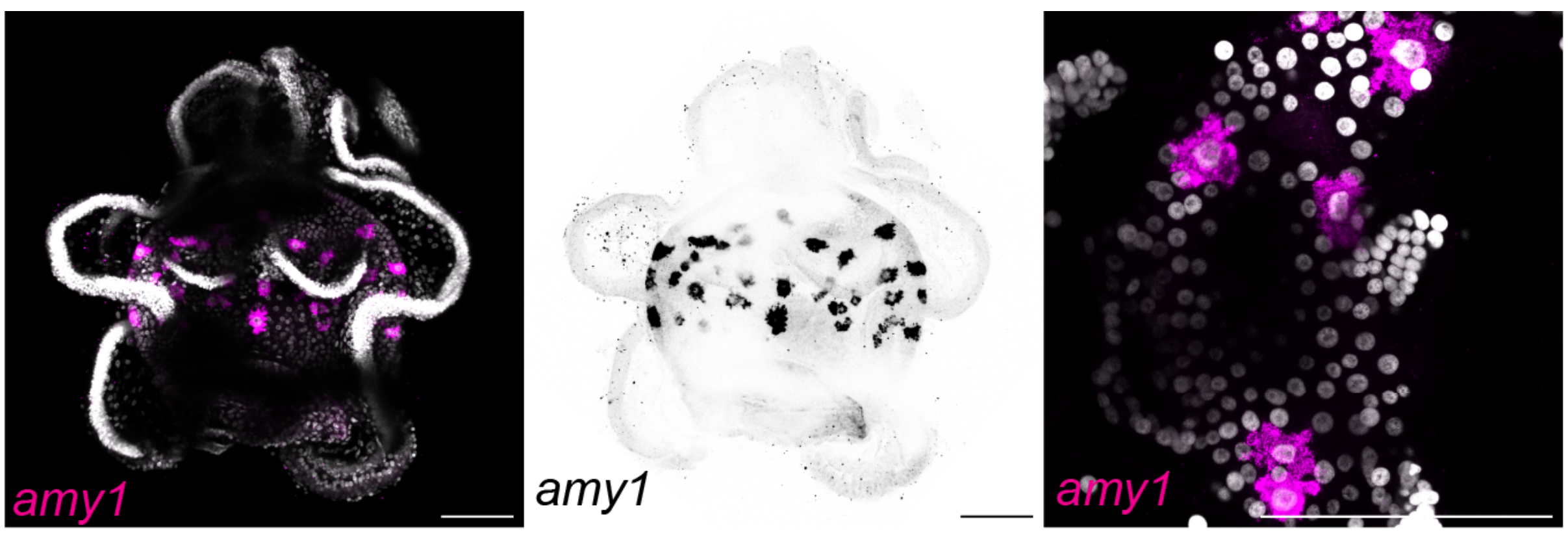

Original Text:
Secretory clusters: 12, 32, 36, 49, 55 (Figure 2R-V, Supplemental Figure 6A-B). Hemichordate epidermis is highly secretory, producing large amounts of mucus from the epidermis, and we found several secretory clusters in both larvae and juveniles. We propose that cluster 12 is a secretory cell population based on the high expression of mucin2 (muc2)(Supplemental Figure 6B). Mucins have been implicated as important for muco-ciliary feeding in hemichordates (Ruppert et al., 1999). HCR imaging showed muc2 expressed in a thin circumferential band of cells in the collar of the juvenile (Figure 2R), similar to the expression of mucins in S. kowalevskii (Simakov et al., 2015). Even for putative secretory clusters with a smaller number of cells (374 for cluster 49) we were able to recover distinct patterns of cell type-specific gene expression. Cluster 49 is characterized by expression of alpha-amylase (amy1). Alpha-amylases hydrolyze 1,4-alpha-glucoside bonds in oligosaccharides and polysaccharides in the salivary gland of humans (Samuelson et al., 1988). HCR imaging showed amy1 expression in a very distinctive pattern of cells surrounding the larval stomach (Figure 2S), so we propose that this cell population may be involved in secretory function. Finally, we found

a cluster of secretory cells with a striking shift in abundance and distribution over developmental time. The markers of a putative secretory cluster 32 included arylsulfatase (arsi), a protease that is upregulated in the Xenopus laevis tadpole tail as an integral part of the resorption process during metamorphosis (Das et al., 2006). In the late larva, these arsi+ cells are found in clusters throughout the ectoderm (Figure 2T), become broadly distributed during metamorphosis (Figure 2U), and finally are predominantly restricted to the most posterior of the early juvenile worm (Figure 2V). Future work will be necessary to investigate the function of this intriguing cell type during metamorphosis.

New Text:

Secretory clusters: 12, 32, 36, 49, 55 (Figure 2R-V, Supplemental Figure 6A-B). We found several clusters in both larvae and juveniles that were enriched for mucins and glycoproteins and organized these clusters into a functional secretory cell class. We propose that cluster 12 is a secretory cell population based on the high expression of *mucin2 (muc2)*(Supplemental Figure 6B). Mucins have been implicated as important for muco-ciliary feeding in hemichordates (Ruppert et al., 1999). HCR imaging showed *muc2* expressed in a thin circumferential band of cells in the collar of the juvenile (Figure 2R), similar to the expression of mucins in *S. kowalevskii* (Simakov et al., 2015). In hemichordates epidermis is highly secretory, producing large amounts of mucus from the epidermis. Even for secretory clusters with a smaller number of cells (374 for cluster 49), we were able to recover distinct patterns of cell type-specific gene expression. Cluster 49 is characterized by expression of *alpha-amylase* (*amy1*). Alpha-amylases hydrolyze 1,4-alpha-glucoside bonds in oligosaccharides and polysaccharides in the salivary gland of humans (Samuelson et al., 1988). HCR imaging showed *amy1* expression in a distinctive pattern of cells surrounding the larval stomach (Figure 2S), so we propose that this cell population may be involved in secretory function. Finally, we found a cluster of secretory cells with a striking shift in abundance and distribution over developmental time. The markers of a putative secretory cluster 32 included *arylsulfatase* (*arsi*), a protease that is upregulated in the *Xenopus laevis* tadpole tail as an integral part of the resorption process during metamorphosis (Das et al., 2006). In the late larva, these *arsi*+ cells are found in clusters throughout the ectoderm (Figure 2T), become broadly distributed during metamorphosis (Figure 2U), and finally are predominantly restricted to the most posterior of the early juvenile worm (Figure 2V). Future work will be necessary to investigate the function of this intriguing cell cluster during metamorphosis.

An interesting question is whether clearly separated larval and juvenile clusters can still reflect lineage relationships, especially in stages where lineage is assumed rather than reconstructed, such as the postmetamorphic juvenile. The authors could consider approaches such as building a cell type tree using a neighbor-joining method, as in Musser et al., 2022 (https://pmc.ncbi.nlm.nih.gov/articles/PMC9233960/). This could highlight lineage relationships of cell types in both larval and juvenile stages and serve as an independent validation of the cluster centroid analysis, strengthening the reader's confidence in and understanding of the results.

Response:
To further investigate potential lineage relationships among larval and juvenile cell types, we additionally implemented a neighbor-joining approach similar to that described by Musser et al. (2022). This analysis recovered relationships broadly consistent with our centroid-based analyses, supporting the robustness of the observed cell-type associations across life-history stages (Supplemental Figure 10).

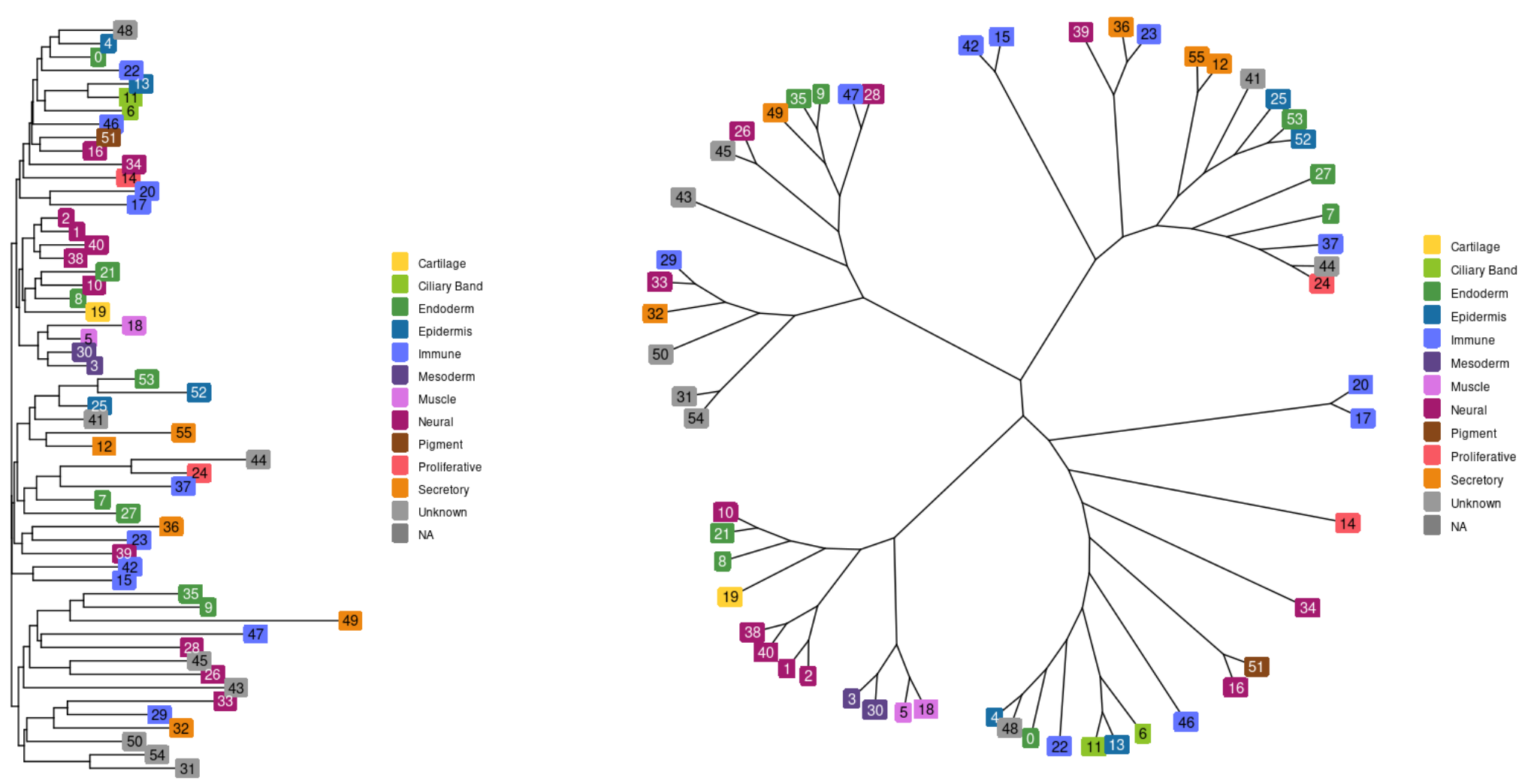

The authors state (lines 433–443) that clustering analyses using only transcription factors and only non–transcription factor genes demonstrate some cell separation by life stage. While this appears true, it is also evident that the UMAP structure partially collapses into a cloud-like shape when using transcription factors alone, compared with the well-structured UMAP in the full analysis. This could suggest a higher similarity of regulatory programs and pleiotropic expression of transcription factors across clusters and cell type classes in hemichordates. We think the authors should further comment on and discuss this point. In contrast, the non–transcription factor analysis is able to recover the overall clustering structure, which is expected because it includes the bulk of terminal differentiation genes that define cell types.

We agree that the transcription factor-only UMAP exhibits less sharply resolved structure than analyses using the full transcriptome or non-transcription factor genes alone. However, we are cautious about overinterpreting these differences because UMAP embeddings represent two-dimensional projections of higher-dimensional transcriptional space and can be sensitive to differences in feature number and variance structure.

Nevertheless, we agree that the broader cloud-like organization observed in the transcription factor analysis may reflect the pleiotropic deployment of regulatory factors across multiple cell classes relative to the more cell-type-restricted expression patterns of terminal differentiation genes. We have added discussion of this possibility in the revised manuscript.

New text:

Although the transcription factor-only embedding exhibited less sharply resolved structure than analyses using the full transcriptome or non-transcription factor genes, we interpret this cautiously because dimensionality reduction can be sensitive to feature number and variance structure. Nevertheless, the broader organization of the transcription factor-only UMAP may reflect the pleiotropic deployment of regulatory factors across multiple cell classes relative to the more cell-type-restricted expression of terminal differentiation genes.

The authors mention the presence of serotonergic neurons that expand during larval development in the Results section. However, there is no mention of this neuronal type in the dataset. Are they absent from the data? If so, the authors should highlight this. Otherwise they should include this neuronal population in their analysis, as it is one of the best-studied cell types in hemichordates.

Serotonergic neurons are present in our dataset based on expression of tryptophan hydroxylase (tph). Although *tph* expression is not restricted to a single cluster, in response to this comment we performed HCR for *tph* and recovered serotonergic cell populations in both larvae and juveniles. These results are now included in Supplemental Figure 5F–G. We additionally reference prior immunohistochemical work describing serotonergic neurons in both larval and adult S. californicum (Gonzalez et al., 2018).

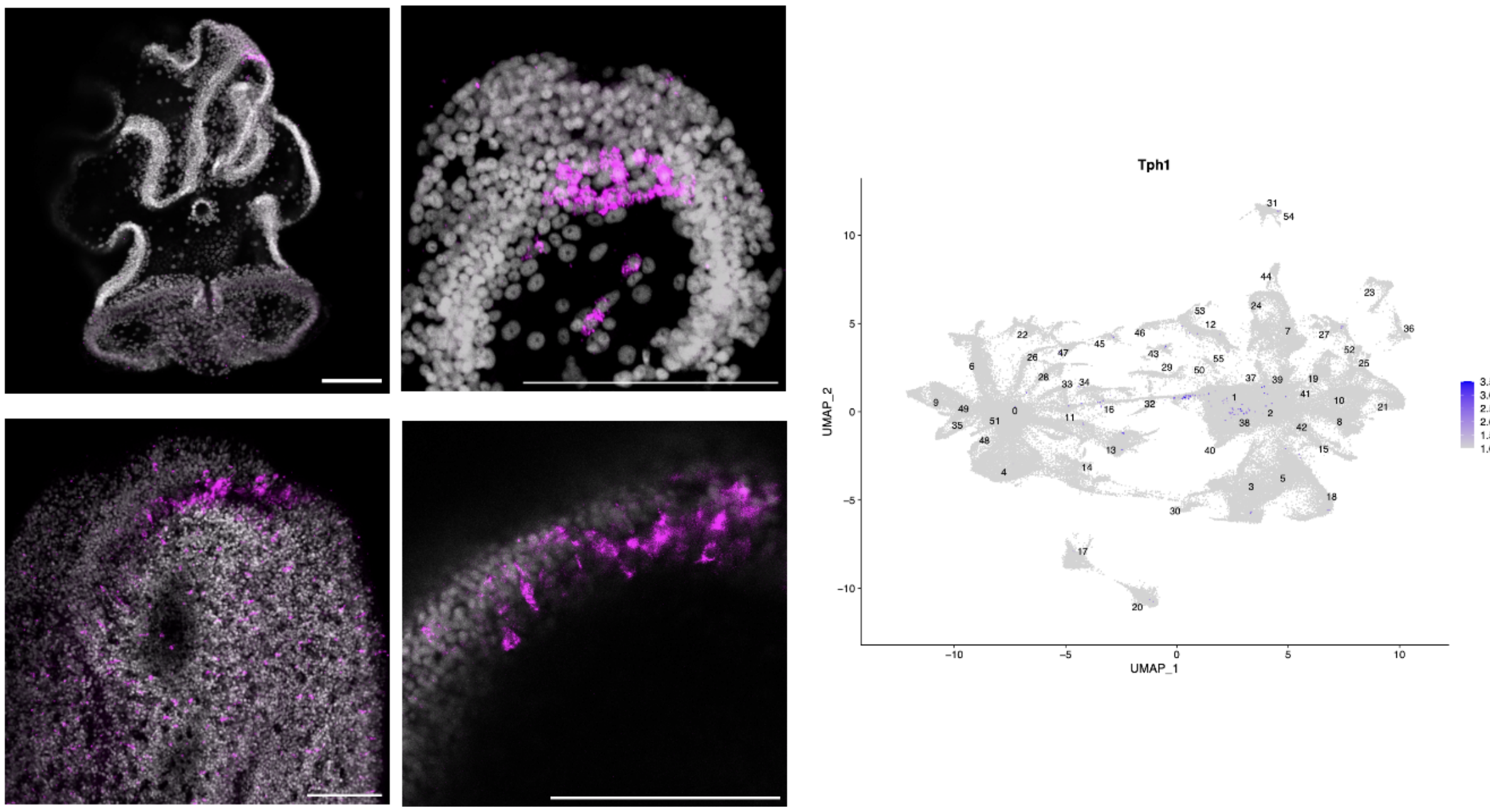

Unless we are mistaken, there are no scripts or links to the scripts related to the analysis provided.

Response:
We have provided an open github repository of our code [https://github.com/0x644BE25/schizocardium_single-cell] which will have all the analysis and plotting code by publication and hope it is valuable for the community.

Minor points:

To a non-expert it is hard to understand which cell types and markers were known before. The authors could modify the respective sections in results to make this more clear.

Response:
As far as we are aware, this is the first study describing broad molecular profiles of hemichordate cell types at single-cell resolution. Our annotations therefore combine previously characterized hemichordate markers with newly identified markers recovered through scRNA-seq analysis. To clarify this distinction, we have extensively revised Supplemental Table 2 to indicate which markers were previously described in hemichordates and which represent newly identified markers from this study. We

additionally include orthology analyses for several representative markers in Supplemental Figure 9.

While the introduction is really well-written we find that a section on how fundamental hemichordates are for evolutionary biology is missing. While many of us already know, it would be great to highlight also here for the non-expert reader.

We have added additional introductory text highlighting the importance of hemichordates for understanding deuterostome evolution, chordate origins, and bilaterian body plan evolution (line 76).

Next text:
Hemichordates are a deuterostome phylum with a relatively simple adult body plan. As the sister group to echinoderms, they occupy a key phylogenetic position for investigating deuterostome and chordate evolution. Their combination of conserved axial patterning programs and comparatively simple morphology has also made them an important system for testing hypotheses of bilaterian body plan evolution (Lowe et al. 2015).

It is not clear what gene-naming strategy the authors are using. For instance, the common abbreviation for dopamine-beta-hydroxylase is dbh and not dopo. We recommend that the authors clearly state the naming strategy used, check all gene names and abbreviations throughout the manuscript, and ensure consistency.

Response:
We reviewed gene nomenclature throughout the manuscript and revised several abbreviations for consistency. Supplemental Table 2 now includes both the full gene names and the abbreviations used in the manuscript. Where possible, we retained nomenclature consistent with previous hemichordate studies and included relevant citations.

Lines 38-39: “a few select species” should read “a few selected species”
Thanks. We have made this change.

Line 136: Are there sphincters that make the gut tripartite? If it would be good to mention.

Response:

We are able to identify the gut as tripartite with a distinctive pharynx, stomach, and intestine with muscle that wraps around the pharynx referred to as pharyngeal muscle (Gonzalez et al. 2018). There is a sphincter between the pharynx and stomach.

Line 361: “These cell population include” should read “These cell populations include”

Thanks. We have made this change.

Line 536: Cell viability was checked with Hoechst and counted on a hemocytometer. It is not clear how Hoechst that labels both dead and alive cells was used to estimate cell viability. Did the authors also use dyes that specifically label alive or dead cells?

Response:
We apologize for the lack of clarity in the original methods description. In preliminary dissociation trials, we assessed viability using flow cytometry with Hoechst to identify nuclei and propidium iodide to specifically label dead cells, which suggested approximately 85% viability. However, because we were concerned that FACS sorting after dissociation could increase cell death, the final experiments used unsorted dissociated cells that were counted directly on a hemocytometer prior to GEM loading. We revised the methods section to clarify this workflow.

Line 570: It is unclear what the reads were mapped to.

As part of our NCBI submission, we are also including the reference that our reads were mapped to.

Supplemental Figure 2 C: the color code suggests that fapb6 expressing cells correspond to cluster 0, but this is in contrast with what reported in panel B of the same figure. Perhaps the authors meant cluster 35, instead? Also in terms of number of cells this appears more likely.

This minor comment by the reviewer has proved to be very insightful for our study. Cluster 0, like many cluster 0s in single-cell RNA sequencing studies often contains the most cells and with that, can be heterogeneous. Indeed, there are not any markers of Cluster 0 that only occur once in our cluster marker list (new Supplemental Table 3).

While fabp6 does occur as a specific marker for Cluster 0 (with a p-value of 2.19e-22 and logFC of .437, it also appears as a marker for Cluster 35 as well (with a p-value of of 6.18e-97 and logFC of 1.99). Fabp6 is likely is a better considered as a characteristic marker of Cluster 35 and we have updated Supplemental Figure 2C accordingly.

Reviewer #4 (Remarks to the Author):

I co-reviewed this manuscript with one of the reviewers who provided the listed reports. This is part of the Nature Communications initiative to facilitate training in peer review and to provide appropriate recognition for Early Career Researchers who co-review manuscripts.

We appreciate this reviewer co-reviewing the manuscript as part of the Early Career Researcher initiative.

REVIEWERS' COMMENTS

We are grateful that the reviewers have taken the time to consider our revised manuscript and appreciate their feedback.

Reviewer #1 (Remarks to the Author):

In the revised version of the manuscript, the authors addressed most of the concerns raised by the reviewer. In particular, the revised explanation of the broad cell-class annotations and intra-class heterogeneity has improved the clarity of the manuscript. However, I still have major concerns which I do not think have been fully addressed.

We appreciate the comments from Reviewer 1 and are glad that they found the revised explanation of cell class annotations and intra-class heterogeneity improved. We address their following points below.

The authors added PAGA connectivity analysis and additional EdU/HCR evidence, but these additions do not fully resolve the original concern. The current PAGA analysis provides a broad graph-based relationship among clusters, but it does not clearly identify cross-stage continuity within specific cell classes or distinguish juvenile populations that may be carried over from larval cells from those that may be newly produced or newly differentiated. I agree that the authors do not need to force a global developmental trajectory across the entire dataset; however, a more stratified analysis may be more informative and biologically appropriate. For example, the authors could consider performing trajectory or cross-stage mapping analyses separately within major cell classes, and/or provide a juvenile–larval cell-state similarity heatmap or continuity score.

We absolutely agree that cross-stage continuity between cell classes is a fascinating question. While out of the scope of this current project, we hope that future work which captures specific cellular populations through metamorphosis will resolve many of the hypothesis we raise here. As the reviewer suggested we did perform the juvenile-larval state similarity heatmap below, however as this did not provide any new information relative to neighbor joining tree analysis we chose to omit it from the final version of the manuscript.

Moreover, for the unknown clusters, marker annotation alone does not fully address their possible developmental origin or fate. The authors suggest that some unknown clusters may represent transient developmental states or specialized cell populations. This is an interesting possibility, but it would be strengthened by placing these unknown clusters into a more explicit developmental or transcriptional context. For instance, unknown clusters could be included in the class-level trajectory or similarity analyses together with neighboring annotated cell classes. HCR validation of one or two representative unknown-cluster markers, especially for stage-specific or metamorphosis-enriched unknown clusters, would also substantially strengthen this

interpretation.

The unknown clusters pose many interesting questions, some of which the reviewer raises here. Many of them are unknown because we are unable to resolve a consensus molecular profile for this population. As well as resolving their identity knowing their stage specific role is also intriguing. We hope by including these populations, both in their own new supplemental figure but also still highlighting them in our cluster composition by life stage plot (Figure 3B) and neighbor joining tree analysis (Figure 4B) will allow future researchers a foundation to study these populations. Additionally, we believe the developmental stages that are stage specific even within our well annotated cell classes will provide fertile ground for future work (Line 263-264).

Reviewer #2 (Remarks to the Author):

The authors have satisfactorily addressed my previous comments, and the manuscript is now improved. I commend the authors for their beautiful work.

We are glad that the reviewer has felt we address their previous comments sufficiently and that they have appreciated the work.

Reviewer #2 (Remarks on code availability):

The code is sufficient.

We are happy to provide it!

Reviewer #3 (Remarks to the Author):

The authors have adequately addressed our previous comments/suggestions.

Minor comment in Fig2 panels R and U there is a white vertical line visible, possibly a result from cropping the images. Please edit accordingly.

We have checked Figure 2 R and U to ensure the white vertical line is no longer visible. We appreciate the reviewers' suggestions.
