# Supplementary material for: Distinct cell states define larval and adult body plans in a hemichordate
Source: Nat Commun. 2026 Sep 8;17:9358. doi: 10.1038/s41467-026-77191-y (PMC13554239; doi:10.1038/s41467-026-77191-y)
Supplement: Supplementary file 3 [file 41467_2026_77191_MOESM3_ESM.pdf]

Description of Additional Supplementary Files

File Name: Supplementary Data 1
Description: Cluster Info

File Name: Supplementary Data 2
Description: Cell Class Info

File Name: Supplementary Data 3
Description: Cluster Markers

File Name: Supplementary Data 4
Description: HCR Info
