# Supplementary material for: Distinct cell states define larval and adult body plans in a hemichordate
Source: Nat Commun. 2026 Sep 8;17:9358. doi: 10.1038/s41467-026-77191-y (PMC13554239; doi:10.1038/s41467-026-77191-y)
Supplement: Supplementary file 4 [file 41467_2026_77191_MOESM8_ESM.pdf]

nature portfolio

Corresponding author(s): Christopher J. Lowe

Last updated by author(s): 2026-August-03

Reporting Summary

Nature Portfolio wishes to improve the reproducibility of the work that we publish. This form provides structure for consistency and transparency in reporting. For further information on Nature Portfolio policies, see our Editorial Policies and the Editorial Policy Checklist.

Statistics

For all statistical analyses, confirm that the following items are present in the figure legend, table legend, main text, or Methods section.

n/a	Confirmed	
☐	☒	The exact sample size (*n*) for each experimental group/condition, given as a discrete number and unit of measurement
☒	☐	A statement on whether measurements were taken from distinct samples or whether the same sample was measured repeatedly
☐	☒	The statistical test(s) used AND whether they are one- or two-sided<br>*Only common tests should be described solely by name; describe more complex techniques in the Methods section.*
☒	☐	A description of all covariates tested
☒	☐	A description of any assumptions or corrections, such as tests of normality and adjustment for multiple comparisons
☐	☒	A full description of the statistical parameters including central tendency (e.g. means) or other basic estimates (e.g. regression coefficient) AND variation (e.g. standard deviation) or associated estimates of uncertainty (e.g. confidence intervals)
☐	☒	For null hypothesis testing, the test statistic (e.g. *F*, *t*, *r*) with confidence intervals, effect sizes, degrees of freedom and *P* value noted<br>*Give P values as exact values whenever suitable.*
☒	☐	For Bayesian analysis, information on the choice of priors and Markov chain Monte Carlo settings
☒	☐	For hierarchical and complex designs, identification of the appropriate level for tests and full reporting of outcomes
☒	☐	Estimates of effect sizes (e.g. Cohen's *d*, Pearson's *r*), indicating how they were calculated

Our web collection on statistics for biologists contains articles on many of the points above.

Software and code

Policy information about availability of computer code

Data collection	N/A
Data analysis	https://github.com/0x644BE25/schizocardium_single-cell

For manuscripts utilizing custom algorithms or software that are central to the research but not yet described in published literature, software must be made available to editors and reviewers. We strongly encourage code deposition in a community repository (e.g. GitHub). See the Nature Portfolio guidelines for submitting code & software for further information.

Data

Policy information about availability of data

All manuscripts must include a data availability statement. This statement should provide the following information, where applicable:

- Accession codes, unique identifiers, or web links for publicly available datasets
- A description of any restrictions on data availability
- For clinical datasets or third party data, please ensure that the statement adheres to our policy

The sequencing raw reads and data generated in this study have been deposited in the NCBI BioProject database under the accession code PRJNA1297089.

Research involving human participants, their data, or biological material

Policy information about studies with human participants or human data. See also policy information about sex, gender (identity/presentation), and sexual orientation and race, ethnicity and racism.

Reporting on sex and gender	N/A
Reporting on race, ethnicity, or other socially relevant groupings	N/A
Population characteristics	N/A
Recruitment	N/A
Ethics oversight	N/A

Note that full information on the approval of the study protocol must also be provided in the manuscript.

Field-specific reporting

Please select the one below that is the best fit for your research. If you are not sure, read the appropriate sections before making your selection.

☒ Life sciences ☐ Behavioural & social sciences ☐ Ecological, evolutionary & environmental sciences

For a reference copy of the document with all sections, see nature.com/documents/nr-reporting-summary-flat.pdf

Life sciences study design

All studies must disclose on these points even when the disclosure is negative.

Sample size	Sample size of number of animals for single cell RNA sequencing was chosen by initial trial experiments to target 10,000 cells per 10x GEM capture.
Data exclusions	Cells were filtered to those with at least 500 and less than 57,000 unique molecular identifiers (UMIs) and >90 and no more than 980 genes. Clustering was done including an additional sample enriched for juvenile proboscis cells, however these cells were excluded in downstream analyses to prevent bias from this sample.
Replication	The early larva time point and juvenile proboscis sample was collected separately from the late larva, during metamorphosis, early juvenile and late juvenile sample, Even though these samples were collected from different cohorts of individuals the life history separation was replicated across experiments.
Randomization	N/A
Blinding	N/A

Reporting for specific materials, systems and methods

We require information from authors about some types of materials, experimental systems and methods used in many studies. Here, indicate whether each material, system or method listed is relevant to your study. If you are not sure if a list item applies to your research, read the appropriate section before selecting a response.

Materials & experimental systems

n/a	Involved in the study	
☒	☐	Antibodies
☒	☐	Eukaryotic cell lines
☒	☐	Palaeontology and archaeology
☐	☒	Animals and other organisms
☒	☐	Clinical data
☒	☐	Dual use research of concern
☒	☐	Plants

Methods

n/a	Involved in the study	
☒	☐	ChIP-seq
☒	☐	Flow cytometry
☒	☐	MRI-based neuroimaging

Animals and other research organisms

Policy information about studies involving animals; ARRIVE guidelines recommended for reporting animal research, and Sex and Gender in Research

Laboratory animals	Schizocardium californicum larvae and juveniles were grown from fertilized zygotes.
Wild animals	Adult Schizocardium californicum were collected in Morro Bay State Park, California, in a mudflat located at 35°20′56.7"N 120°50′ 35.6"W with appropriate state permitting from California Department of Fish and Wildlife. Per CDFW adults were euthanized after spawning and not returned to the field site.
Reporting on sex	Sex and gender were not collected in these studies. While hemichordates do have separate male and female sexes as adults, these differences were not present in the life history stages we studied.
Field-collected samples	Animals were spawned as described in Gonzalez et al. 2018 with individual females transferred in bowls of filtered seawater (FSW) and placed in an illuminated incubator at 24–26°C.
Ethics oversight	Ethics approval was not required, however adult animals were euthanized according to CDFW permitting guidelines.

Note that full information on the approval of the study protocol must also be provided in the manuscript.

Plants

Seed stocks	N/A
Novel plant genotypes	N/A
Authentication	N/A
